# Sugar-Based Ionic Liquids: Multifaceted Challenges and Intriguing Potential

**DOI:** 10.3390/molecules26072052

**Published:** 2021-04-03

**Authors:** Valerio Zullo, Anna Iuliano, Lorenzo Guazzelli

**Affiliations:** 1 Dipartimento di Chimica e Chimica Industriale, Università di Pisa, via Moruzzi 13, 56124 Pisa, Italy; valerio.zullo@phd.unipi.it (V.Z.); anna.iuliano@unipi.it (A.I.); 2 Dipartimento di Farmacia, Università di Pisa, via Bonanno 33, 56126 Pisa, Italy

**Keywords:** sugar-based ionic liquids, bio-based ionic liquids, sustainable ionic liquids, green solvents

## Abstract

Carbohydrates represent a promising option in transitioning from oil-based chemical resources to renewable ones, with the goal of developing chemistries for a sustainable future. Cellulose, hemicellulose, and largely available monosaccharides already provide useful chemical building blocks, so-called platform chemicals, such as levulinic acid and hydroxymethyl furfural, as well as solvents like cyrene or gamma-valerolactone. Therefore, there is great anticipation for novel applications involving materials and chemicals derived from sugars. In the field of ionic liquids (ILs), sugar-based ILs have been overlooked for a long time, mainly on account of their multistep demanding preparation. However, exploring new strategies for accessing sugar-based ILs, their study, and their exploitation, are attracting increasing interest. This is due to the growing concerns about the negative (eco)toxicity profile of most ILs in conjunction with their non-sustainable nature. In the present review, a literature survey concerning the development of sugar-based ILs since 2011 is presented. Their preparation strategies and thermal behavior analyses, sorted by sugar type, make up the first two sections with the intention to provide the reader with a useful guide. A final overview of the potential applications of sugar-based ILs and their future perspectives complement the present analysis.

## 1. Introduction

Ionic liquids (ILs) are organic salts that melt below an arbitrary limit set at 100 °C [1]. The cation is generally unsymmetrical, rather bulky, and of organic nature, while the anion can be either of organic or inorganic nature. The main attractive feature of ILs is the possibility to finely tune their physical and chemical properties, such as melting point, viscosity, density, and hydrophilicity, through judicious anion-cation pairing or by the modification of their chemical structure. The possible combinations of constituting ions are so vast that a certain set of desired properties can, in principle, always be achieved by targeted selection of a specific structural motif. These unique features made ILs the prototypical “designer solvents” [2] or “designable” molecules. The potential applications of ILs have been studied in a number of constantly growing and diverse areas of research, which include, but are not limited to, solvent systems or catalysts for organic reactions [3], electrolytes for batteries [4], biomasses dissolution, fractionation and modification [5], new IL-active pharmaceutical ingredients and drug delivery systems [6].

The green character of these neoteric solvents has been praised since the beginning of the ILs era. This aspect is strictly related to their negligible vapor pressure and low flammability, which makes them a safe alternative to traditional volatile organic solvents. However, during the years, studies related to the (eco)toxicity and biodegradability of ILs [7,8,9] raised some concern about the real green profile of these solvents. These reports also showed the need for an in-depth, multilevel investigation aimed to determine the key features of each IL system and to prevent baseless generalizations.

Two further aspects need to be considered when dealing with the whole life cycle of an IL, namely, the energetic and environmental impact of the synthetic pathways to prepare them and the fossil-derived nature of most of the traditional constituent ions.

One of the most prominent approaches commonly used to address the issues stated above is the use of building blocks obtainable from natural, renewable sources for preparing the so-called bio-based ILs. In this frame, various naturally occurring compounds have been employed as starting material, such as amino acids, fatty acids, and carbohydrates [10,11,12,13]. It is also worth mentioning that platform chemicals such as levulinic acid and 5-(Hydroxymethyl)furfural (HMF), which can also be obtained from cellulose and chitin, have also been exploited for the preparation of ILs [14,15,16].

Looking to carbohydrates as a whole, they are probably the sole possible carbon source that could truly revolutionize the chemistry field and replace fossil-derived chemicals. Indeed, together with the already mentioned platform chemicals, solvents obtained from sugars such as gamma-valerolactone and dihydrolevoglucosenone (Cyrene) are nowadays available on the market, and flourishing research is performed to increase the portfolio of renewable chemicals of low toxicity prepared from sugars. Moreover, it is important to stress that carbohydrates are intrinsically chiral, representing an additional key feature for the preparation of benign, advanced functional materials.

However, sugars’ high density of functional groups represents a challenging counterpart to the aforementioned advantages. Indeed, even the manipulation of the simplest monosaccharides requires careful planning and often complex protection-deprotection strategies. This issue is particularly demanding when preparing sugar-based ILs and especially when their use as solvents is envisaged. Therefore, the ease of synthesis of carbohydrate-based ILs is a critical part, to the point that it still limits the full exploitation of these innovative materials.

Finally, it is worth mentioning a recent impressive result reported by D’Anna et al. [17], who showed how attaching a sugar pendant to an imidazolium cation resulted in an IL with reduced toxicity towards Zebrafish embryos. The modification of established ILs with carbohydrate portions, with the aim of reducing their toxicity issues, deserves further investigation and highlights the potential of this approach for the development of better ILs.

Overall, carbohydrates hold great promise as components of the next generation’s ILs, characterized by reduced environmental impact in their synthesis and in their applications. The present review covers the papers on carbohydrate-derived ILs published since 2011, while previous relevant literature on this topic can be found in a review by Chiappe et al. [18].

All those salts that possess a sugar core and fit into the IL definition have been included in the present review, although they were not clearly considered ILs in the original work; this refers, in particular, to sugar-containing ionic surfactants. Conversely, ionic liquid tagged (IL-TAG) carbohydrates, employed, for instance, in the development of catch-and-release oligosaccharide protocols [19], which are often used as synthetic intermediates and poorly characterized from the IL perspective, will not be discussed in this literature survey.

The review is organized in three sections where the critical aspects and the intriguing potential of sugar-based ILs are analyzed. In the first section, recent advances in the synthetic approaches to sugar-based ILs will be presented. In turn, each subsection will focus on a single stereochemical configuration when dealing with monosaccharides or a single class of sugar derivatives (i.e., uronic acids, isohexides).

Following a similar classification, the second section will report on the thermal properties of this family of ILs. Indeed, liquid range and thermal stability are important properties to define the scope of use of ILs. Finally, the last section will provide an overview of the field of application of sugar-based ILs.

## 2. Synthesis

The synthesis of ionic liquids (ILs) starting from carbohydrates can be performed following two main approaches: In the first one, the ionization is obtained by an acid-base reaction of sugar bearing an acidic (or basic) functional group with a suitable basic (or acidic) compound; the second one is based on the selective derivatization of a functional group present on the saccharide moiety with an ionic appendage.

The first strategy, which is often referred to as neutralization strategy, is generally simpler than the second approach, while the derivatization strategy takes advantage of carbohydrate chemistry. Given that different sugars are characterized by different functionalization and/or stereochemistry, the synthetic strategy of choice mainly depends on the nature of starting material.

For example, the neutralization approach is very common when the synthesis begins with carbohydrates possessing an acidic (i.e., uronic acids) or basic (i.e., glucosamine) group in their structure, while the derivatization approach is more common in most of the other cases. Some carbohydrate reactions have broad application and utility over different sugar-type compounds, whereas some specific solutions have been developed which take advantage of the structure or the stereochemistry of the starting material saccharide of interest. This is particularly true in the case of ionic liquids prepared through the derivatization approach.

On the basis of the previous considerations, the synthesis of ionic liquids from carbohydrates is divided into subsections covering different sugars. A critical evaluation of the reported protocols is presented.

The physical state of every ionic liquid cited in the following discussion is collected in Appendix A.

### 2.1. d-galactose

d-galactose, a very common natural monosaccharide found in glycoproteins, glycolipids, and proteoglycans, is a *C-4* epimer of glucose and can be industrially obtained from the enzymatic hydrolysis of lactose, a byproduct of the dairy industry, which is a disaccharide characterized by the β 1 → 4 linkages between d-galactose and d-glucose [20].

The synthesis of ILs from galactose is not common. From 2011 until now, only a few papers reporting the preparation of ILs from this building block have been published.

Two synthetic strategies have been developed to this aim, both based on the selective derivatization of a hydroxyl group to introduce a cationic appendage.

**Scheme 1:**
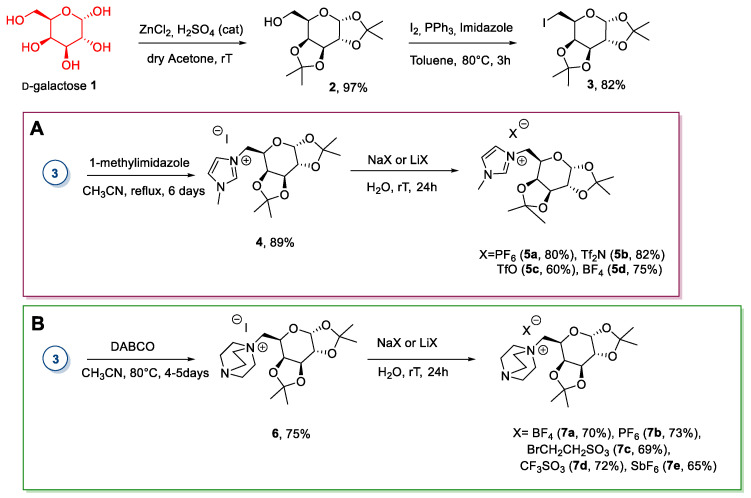
Synthesis of ionic liquids (ILs) from d-galactose, as reported by Jayachandra et al. [21,22,23], (**A**) and the one, as reported by Kaur and Chopra [24] (**B**).

The first approach, developed by Jayachandra et al. [21,22,23], and Kaur and Chopra [24], involved the selective protection of the secondary alcohols as acetals, the derivatization of the free primary hydroxyl functionality to obtain a good leaving group, and a final S_N_2 reaction to give an ionic compound (Scheme 1).

The first step of the synthesis was the selective protection of the secondary hydroxyl groups as acetals through the reaction of d-galactose with dry acetone in the presence of ZnCl_2_ and catalytic amounts of H_2_SO_4_ [25]. The free primary hydroxyl group was then derivatized through an Appel reaction to give the corresponding iodide **3** [26,27]. Compound **3** was then reacted with a good nitrogen nucleophile (1-methylimidazole [21,22,23] or DABCO [24]) in an S_N_2 fashion to give the desired ionic compound. This reaction is the weak point of the synthesis: It is very slow, and some days of heating under reflux were needed to obtain complete conversion and a good yield. A final anion metathesis with suitable potassium or lithium salts gave ILs a more hydrophobic character. This reaction was carried out in the water, from which the pure products **7b** and **7e** precipitated once they formed (Scheme 1).

It is important to note that, even if the authors defined compounds **4**, **5**, **6**, and **7** as ILs, only **5b** had a melting point below 100 °C; compounds **6** and **7** were obtained as room temperature solids, and no information on their melting point was reported, while all the other derivatives melted over 100 °C (see Appendix A). Furthermore, in these works, the authors reported l-galactose as a sugar unit even if they said that the starting material was d-galactose. However, despite these aspects, these compounds are included in our review.

**Scheme 2:**
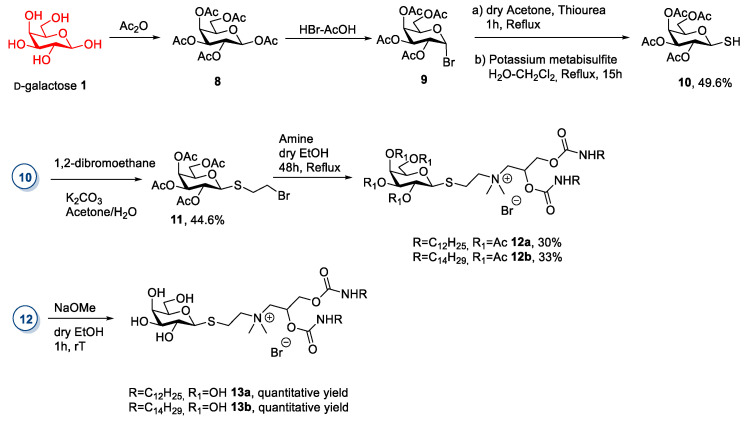
The synthesis of ILs from d-galactose, as reported by Qiao et al. [28].

A slightly different approach was applied by Qiao et al., in 2014 [28]. (Scheme 2) In this study, d-galactose was subjected to a peracetylation reaction [29] followed by treatment with HBr to afford the α-anomer bromide **9** [29]. (Scheme 2) Compound **9** was then reacted with a sulfur nucleophile in an S_N_2 reaction to yield thio-galactoside **10**, which had a β-anomeric configuration [30]. To obtain the final ionic compounds, the authors derivatized compound **10** with 1,2-dibromoethane so that the product contained a good leaving group, which was displaced with a tertiary amine, thus leading to a quaternary salt. A final deacetylation gave the ionic compound with free hydroxyl groups (Scheme 2). Only compound **12a** is an IL, whereas for the other compounds, no melting point detail was reported.

This synthetic route is very time-consuming: The derivatization of the sugar moiety was very long, and the purification of compounds **10**, **11**, **12** was made through silica gel chromatography. Moreover, the tertiary amines [31] employed for the quaternization step are not commercially available, but they have to be synthesized *ex-novo* and purified through column chromatography.

**Scheme 3:**
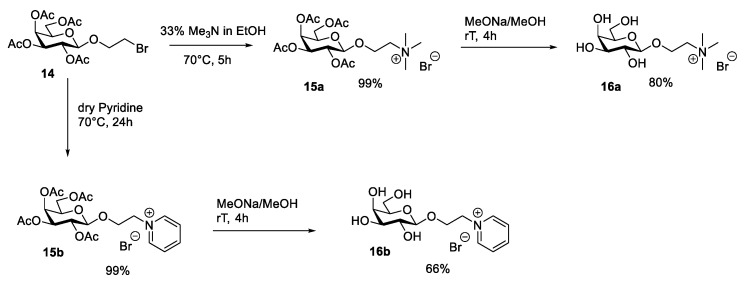
The synthesis of ILs, as reported by Dmochowska et al. [32].

A similar, but shorter, synthetic route was followed by Dmochowska et al. in 2011 (Scheme 3) [32]. The authors started from the previously reported 2-bromoethyl-tetra-*O*-acetyl-β-galactopyranoside **14**, obtained through a selective introduction of the 2-bromoethoxy group at the anomeric carbon of peracetilated galactose (as in the case of d-glucose (see Section 2.6 for further discussion)). Then, the displacement of the bromide by tertiary amines gave the ionic products **15** that were deprotected, affording the final desired compounds **16** (Scheme 3). This route is very simple, consisting of two high-yield, simple steps. The relatively demanding task is represented by synthesizing starting material **14**, as discussed more in detail in Section 2.6 for d-glucose.

**Scheme 4:**
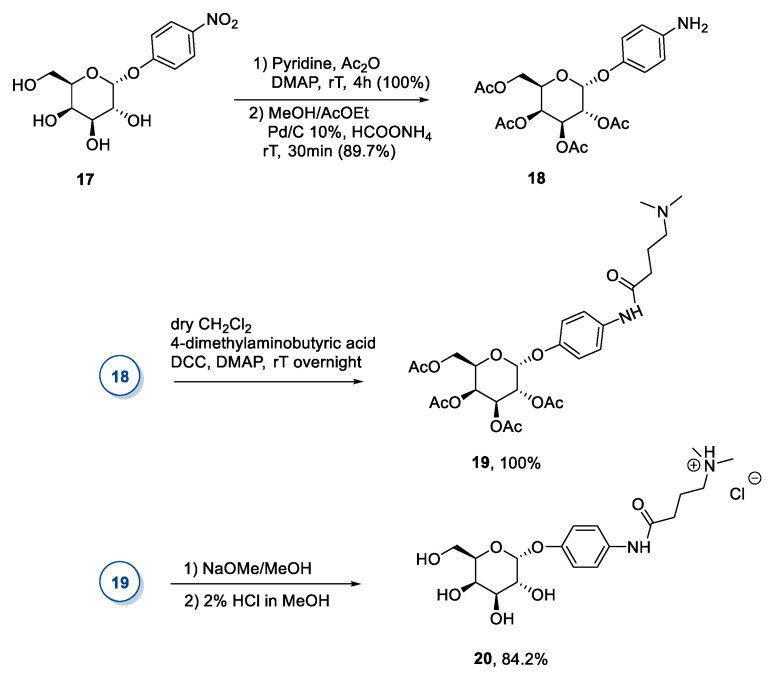
The synthesis of a new IL from d-galactose, as reported by Ahmad et al. [33].

A derivative of d-galactose was used by Ahmad et al. in 2015 (Scheme 4) [33]. The authors started from *p*-nitrophenyl-α-d-galactopyranoside **17** to obtain the desired compound **20** after peracetylation with Ac_2_O in pyridine, reduction with Pd/C of the nitro group, coupling of the resulting aniline **18** with 4-dimethylamino butyric acid to give amide **19** followed by deacetylation and protonation (Scheme 4). This synthetic route distinguishes itself for the starting material employed, but it still presents the drawback of laborious chromatographic purifications.

### 2.2. d-xylose

d-xylose is a pentose sugar found in great amounts in plants: Indeed, hemicellulose is the main source of this sugar. The synthesis of ILs from xylose is a recent development, with the first report published in 2013 [34]. As for d-galactose (see Section 2.1), the main approach for synthesizing ILs from xylose starts from the selective derivatization of one (or two) of its hydroxyl groups.

**Scheme 5:**
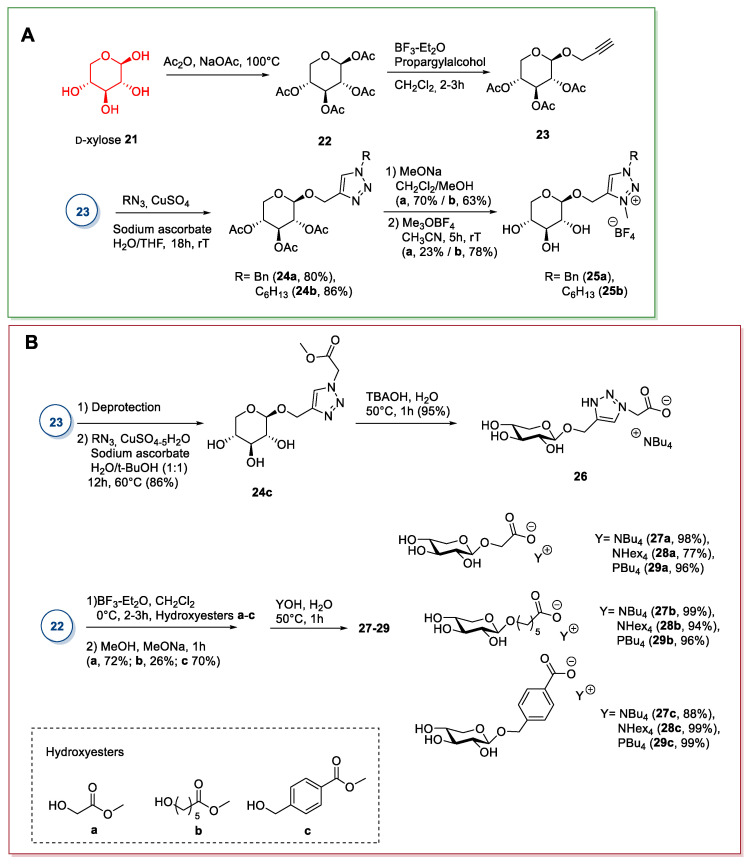
The synthesis of d-xylose ILs, as reported by Ferlin [34] (**A**) and Gatard [38] (**B**).

A first strategy involves the peracetylation of d-xylose with NaOAc in Ac_2_O at 100 °C under kinetic control [35], followed by selective displacement of the anomeric acetate in the presence of BF_3_·Et_2_O [35,36] to give the pure β-glycoside. This way, a functionalized pendant can be inserted, which can be further derivatized to give the desired ILs (Scheme 5). The β-anomer of peracetylated xylose is preferred to the α-anomer for the glycosylation reaction because it is more reactive in the presence of a Lewis acid, due to the participation of neighboring *trans* acetate group at the C2-position. The displacement reaction proceeds through an S_N_1 mechanism and is based on the selective formation of a bridged oxacarbenium cation at the anomeric position, which selectively leads to β-xylopyranosides under kinetic conditions [37].

This approach was employed by Ferlin et al. [34] in 2013 (Scheme 5A); the peracetylated compound **22** was reacted with propargyl alcohol to obtain the alkynyl compound **23**, which was then used in a Huisgen 1,3-dipolar cycloaddition reaction. The triazole xylosides **24a,b** were deacetylated and quaternized with trimethyloxonium tetrafluoroborate to give the desired ILs (Scheme 5A). The weak point of this route is the Huisgen 1,3-dipolar cycloaddition, given that to obtain a good yield of compound **24**, a large excess of the copper salt is needed.

The same approach was used by Gatard et al., in 2017 [38], to obtain compound **26** (Scheme 5B). In this case, the sugar moiety was used to obtain the anionic part of the chiral ionic liquid (CIL) by reacting **23** with azido methylacetate, followed by the hydrolysis of the ester group with tetrabutylammonium hydroxide (TBAOH) (Scheme 5B). In this work, the authors also developed a slightly different strategy to obtain anionic xylose-derived ILs from intermediate **22** through selective anomeric derivatization with different hydroxy esters, followed by hydrolysis with tetra-alkylammonium or tetra-alkyl phosphonium hydroxides (Scheme 5B). In general, no chromatographic purifications were needed. An interesting point regarding this route is the possibility of obtaining anionic sugar-derived CILs.

**Scheme 6:**
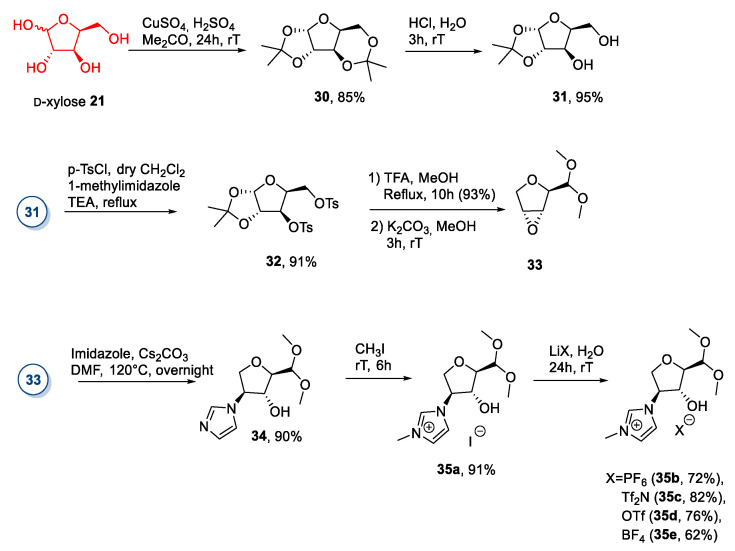
The synthesis of ILs from d-xylose, as reported by Jayachandra et al. [39,40].

A less straightforward approach was employed by Jayachandra et al. in 2016 (Scheme 6) [39,40]. Furthermore, in this work, the authors started with selective protection-deprotection steps of the xylose hydroxyl groups (as acetals) followed by activation of the free alcohols as *p*-toluensulfonic esters (Scheme 6). At this point, epoxide **33** was synthesized from **32** through an acid-promoted ring migration reaction, followed by treatment with potassium carbonate in methanol at room temperature. Epoxide **33** was opened through a nucleophilic attack of imidazole, and the ionic iodide **35a** was obtained after quaternization with MeI. An anion metathesis was applied to give different ILs containing the same cationic sugar moiety (Scheme 6). This synthesis is interesting, given the unusual and elegant reaction sequence employed. However, a major drawback of this protocol is its length and the need to purify some compounds via column chromatography. 

### 2.3. d-gluconic Acid

**Figure 1:**
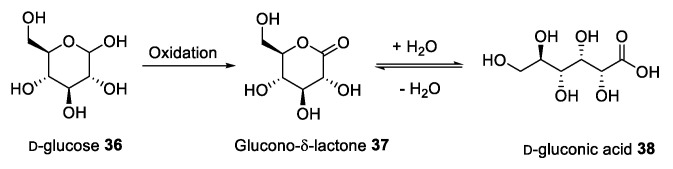
d-gluconic acid production and its two forms.

d-gluconic acid is a natural organic acid deriving from the oxidation of d-glucose. This compound, obtained from food waste [17,41,42], can be found in two forms: The cyclic glucono-δ-lactone **37**, and the linear d-gluconic acid **38** (Figure 1).

Gluconic acid and its derivatives are produced in nature from the oxidation of glucose by bacteria and fungi [17], and can be found in mammalian organisms as intermediates in carbohydrate metabolism. These compounds are generally non-toxic and biodegradable both under aerobic and anaerobic conditions [43].

Gluconic acid has been extensively used for the synthesis of new ILs. Its characteristic structure makes it a very interesting building block: The chemoselective derivatization of the carboxylic group can be easily achieved, which avoids tedious protection and deprotection steps. Moreover, in its free acidic form, gluconic acid can be used directly and without any derivatization for the synthesis of ILs, through a simple neutralization reaction with basic compounds.

**Scheme 7:**
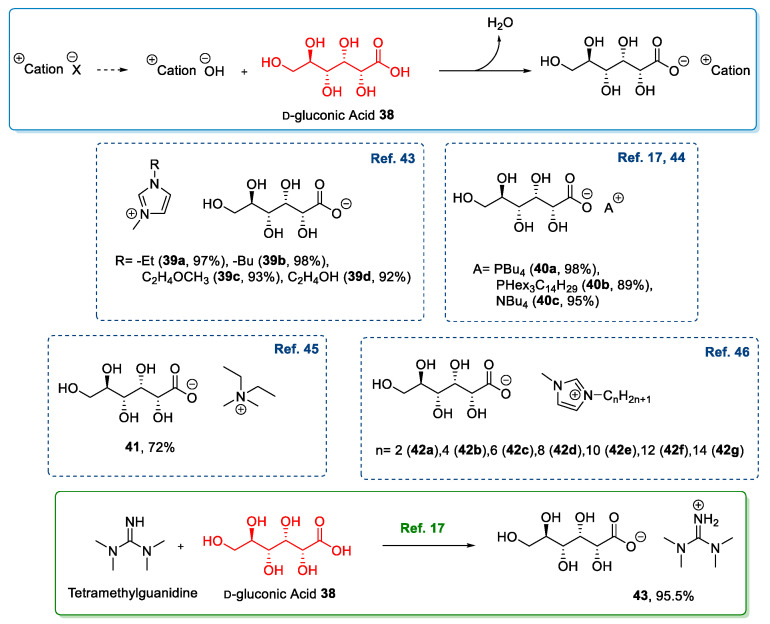
Synthesis of ILs from d-gluconic acid through the neutralization reaction and some examples of final ILs [17,43,44,45,46].

The above-mentioned strategy, based on the neutralization-metathesis reaction of d-gluconic acid in its linear form, has been widely used [17,43,44,45,46]. This approach uses as a reagent the hydroxide of a suitable organic cation, in turn, obtained by simple anion exchange from a neutral salt, and produces the desired IL and an easy-to-remove byproduct as water [17,43,44,45,46]. Not only could these compounds be used as a bases, but even guanidine was used to obtain a direct acid-base reaction without the formation of any byproduct (Scheme 7) [17].

**Scheme 8:**
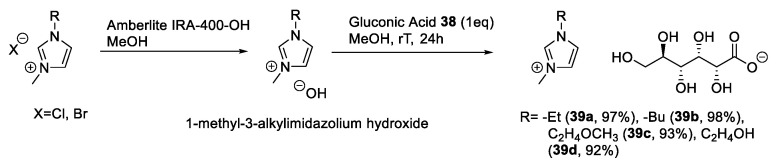
The synthesis of ILs from d-gluconic acid, as reported by Costa et al. [43].

As a representative example, we can consider the work published by Costa et al. in 2015 (Scheme 8) [43]. In this work, the authors started from commercially available 1-methyl-3-alkyl imidazolium chloride or bromide ILs. To obtain the desired hydroxide, these building blocks were dissolved in methanol and passed through an anion exchange column. The resulting solutions were added to gluconic acid dissolved in methanol, and the mixture was stirred for 1–2 h. The pure product was obtained after removing the solvent under reduced pressure (Scheme 8).

This approach is fast, simple, and prepares different CILs in quantitative yield. A drawback is the limited accessible chemical space that can be explored through this reaction sequence on the anion side. This is due to the particular chemistry of sugars, and to the need for protection/deprotection reactions to achieve selective hydroxyl group manipulations.

**Scheme 9:**
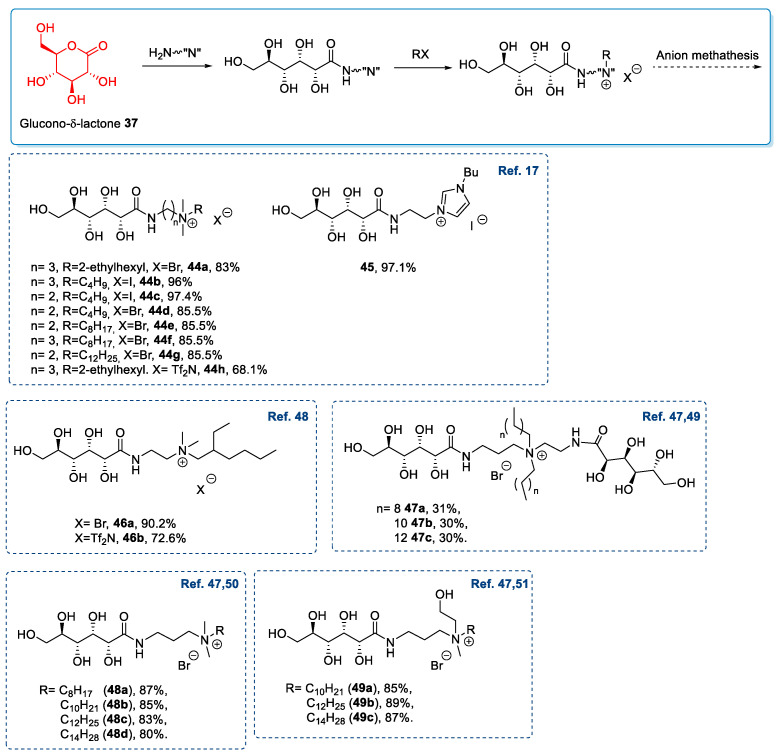
Synthesis of ILs from d-gluconic acid through the amide formation. Only the yield of the final quaternization and/or metathesis reactions are reported [17,47,48,49,50,51].

A complementary strategy is based on the derivatization of the carboxylic group of glucono-δ-lactone **37** to obtain an amide possessing lateral nucleophilic nitrogen in the side chain. A final quaternization step of the nucleophilic nitrogen gives the desired ionic compound (Scheme 9) [17,47,48,49,50,51]. Generally, the amide-formation reaction is a simple procedure: A high yield (85–95%) of chemically pure product is obtained after a simple purification step.

**Scheme 10:**
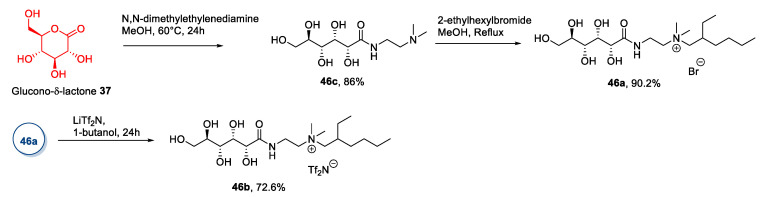
The synthesis of ILs from gluconic acid, as reported by Billeci et al. [48].

In a representative procedure, Billeci et al. [48] in 2019 synthesized compounds **46** from glucono-δ-lactone (Scheme 10). Compound **37** was reacted with *N*,*N*-dimethylethylenediamine for 24 h in methanol at 60 °C. Then the product was quaternized by reacting with 2-ethylhexyl bromide in methanol under reflux to yield the desired product **46a**. This approach is very simple and efficient, also due to the lack of long, demanding purification steps.

To conclude, d-gluconic acid is a very interesting building block for synthesizing novel ILs, and the main approaches used for this aim better comply with the Green Chemistry principles.

### 2.4. d-glucuronic Acid, d-galacturonic Acid, Lactobionic Acid and Glucosamine

**Figure 2:**
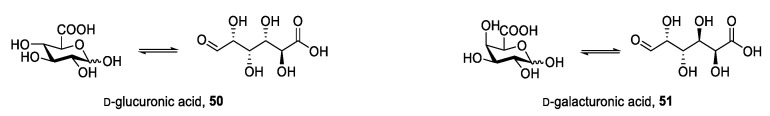
Chemical structure of d-glucuronic acid and d-galacturonic acid.

**Scheme 11:**
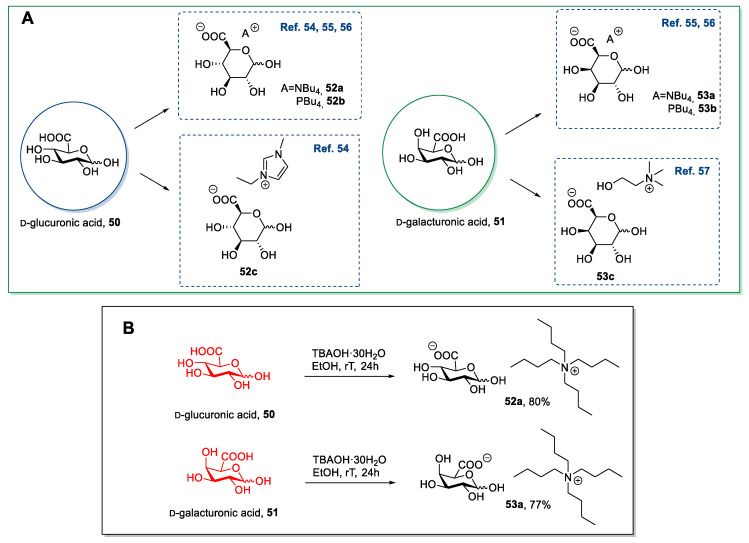
Synthesis of ILs from d-glucuronic acid and d-galacturonic acid (**A**) [54,55,56,57], and the example, as reported by Ferlin et al. (**B**) [55].

Uronic acids, firstly isolated from urine, are a class of monosaccharides whose primary hydroxyl group has been oxidized to carboxylic acid. Two of the principal uronic acids, d-glucuronic, and d-galacturonic acid, are found in various plant biomasses, mainly as a constituent of polysaccharides. In particular, d-glucuronic acid is predominantly found in natural gums [52], while d-galacturonic acid is the main component of pectin (Figure 2) [53].

d-glucuronic acid and d-galacturonic acid have been used in synthesizing new ILs (Scheme 11A) mainly through a neutralization pathway [54,55,56,57]: The uronic acid is typically combined with an alkaline ionic compound (hydroxide or bicarbonate anion). The strategy is the same as for the neutralization of d-gluconic acid (see Section 2.3).

As a representative example, in 2013, Ferlin et al. synthesized a few new ILs starting from d-glucuronic and d-galacturonic acid through a simple neutralization step (Scheme 11B) [55]. In this work, the desired products were obtained in good yields starting from commercially available uronic acid and TBAOH. This approach, as said before for d-gluconic acid (see Section 2.3), is well-suited for synthesizing ILs given the low-cost, the absence of long and demanding purifications, and the good yield. Moreover, only ethanol and acetone, which are sufficiently “green” solvents, are used as reaction media. However, the only path to obtain different structures is represented by the nature of the cationic moiety. Addressing diversity through modification on the sugar part is, in principle, a viable way to expand the portfolio, but only at the expense of challenging chemical manipulations.

**Scheme 12:**
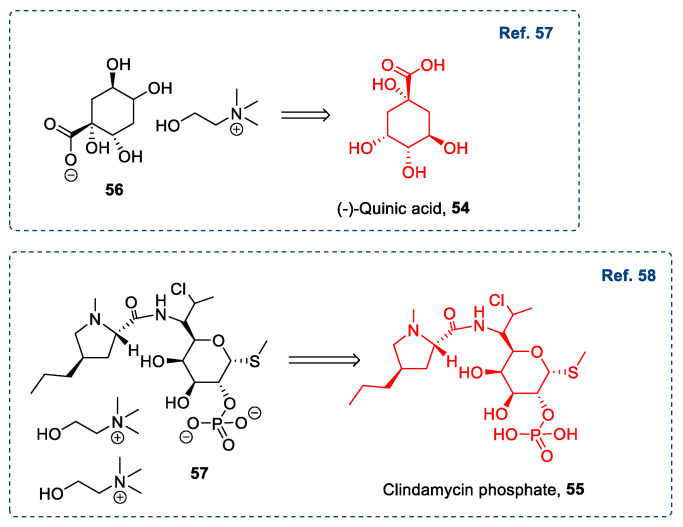
Synthesis of ILs from Clindamycin phosphate [58], and (−)-quinic acid [57].

A similar strategy was also used for synthesizing less common ILs, where the anion was derived from a sugar-containing compound, such as (−)-quinic acid [57] **54** or Clindamycin **55** (Scheme 12) [58].

Lactobionic acid is a disaccharide, derived from the oxidation of lactose, composed of a unit of d-gluconic acid and a unit of β- d-galactose [59]. Lactobionic acid, like other acidic sugars, represents a valuable platform for synthesizing new ionic liquids through easy, low-cost protocols. Despite these features, lactobionic acid has been used for this aim only in two studies from 2011 to now [50,60].

**Scheme 13:**
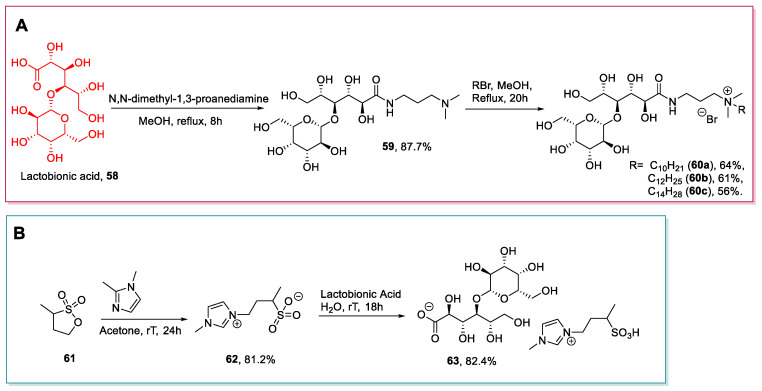
The synthesis of ILs from lactobionic acid, as reported by Zhi et al. (**A**) [50] and by Xu et al. (**B**) [60].

The two main strategies adopted for synthesizing ILs from lactobionic acid are similar to those employed for d-gluconic acid (see Section 2.3): The first one, reported by Zhi et al. in 2013 [50], is based on the chemoselective reaction of the carboxylic group with a diamine to give amide **59**, which is subsequently quaternized (Scheme 13A); the second one, reported by Xu et al. in 2020 [60], is based on the neutralization of the starting material with a basic ionic compound (**62**) (Scheme 13B) [50].

**Scheme 14:**
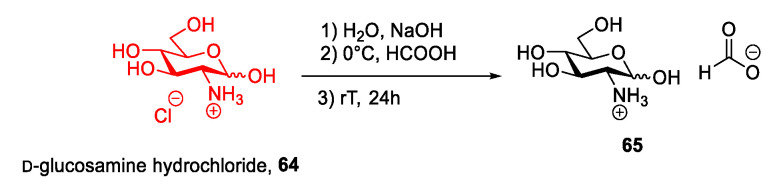
Synthesis of a new IL from d-glucosamine, as reported by Iranpour et al. [61].

To conclude this section, it is of interest to note that the neutralization reaction for synthesizing ILs has been used with a basic sugar and an acidic reagent by Iranpour et al. in 2018 [61] to obtain the glucosamine-based ionic liquid **65** (Scheme 14). Glucosamine, an amino sugar, is the main constituent of the polysaccharides chitin and chitosan. This monosaccharide is mainly obtained through chemical hydrolysis of chitin by extraction with hydrochloridic acid of shrimp and crab shells [62,63].

In their work, Iranpour et al. started from commercially available d-glucosamine hydrochloride **64** that, after neutralization with NaOH to obtain the free amine, was reacted with formic acid in a neutralization reaction to obtain the desired ionic liquid **65**. (Scheme 14) This approach has the same advantages and drawbacks as all the previously discussed neutralization protocols.

### 2.5. Isohexides

**Figure 3:**
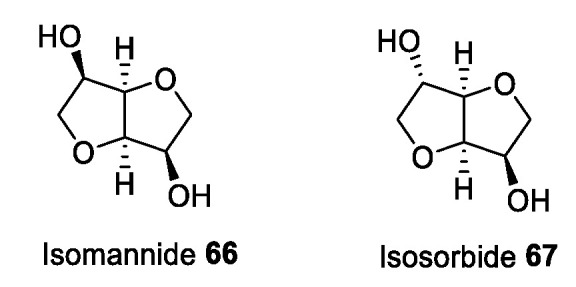
Isomannide and isosorbide.

Isomannide and isosorbide (Figure 3) are two renewable, inexpensive, and commercially available chiral compounds derived from the dehydration of d-mannitol and d-sorbitol, respectively, obtained as waste products during the processing of corn oil [64]. These building blocks are characterized by a *cis*-fused bicyclic rigid structure and by two hydroxyl groups at *C3* and *C6*. These functional groups differ in their stereochemistry, being *endo* in isomannide **66**, while isosorbide **67** has a C3-*exo*/C6-*endo* configuration.

The two most common approaches developed for the derivatization of these compounds are based on: (a) A double derivatization of the hydroxyl groups or (b) a more challenging selective derivatization of a single hydroxy group.

Taking into account this second possibility, isomannide has a C_2_ symmetry, so selective derivatization can be possible only following a statistical approach, while the two hydroxyl groups of isosorbide are not chemically equivalent, and in principle, can be regioselectively manipulated.

From 2011 to now, only a few examples of ionic liquids starting from these compounds have been published. These works are mainly based on direct derivatization of at least one hydroxyl functionality to introduce a good leaving group (tosylate or triflate) followed by a displacement reaction with a nitrogen nucleophile to yield an ionic compound. An anion metathesis reaction can be used to obtain different ILs containing the same cation [65,66,67].

**Scheme 15:**
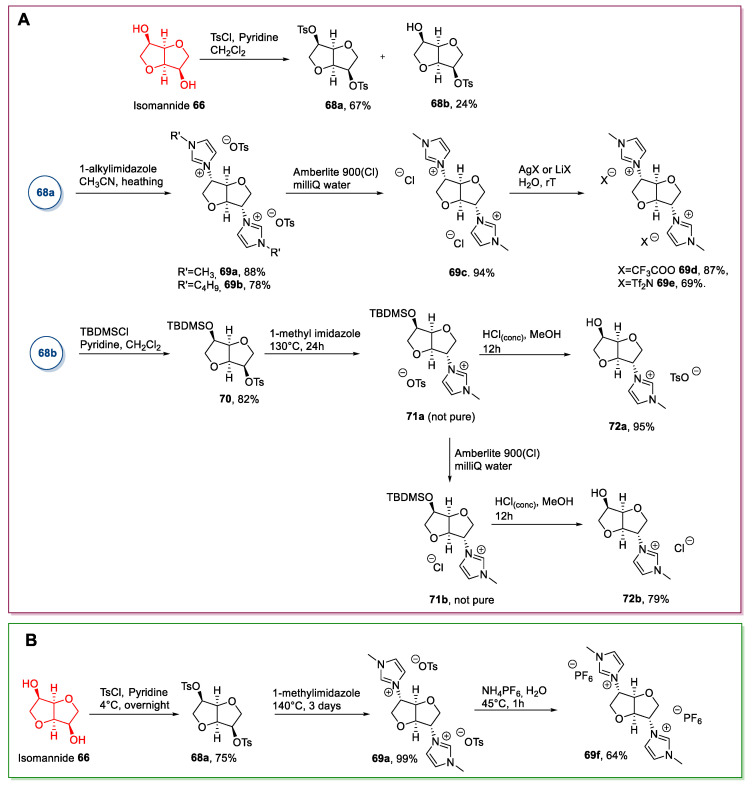
The synthesis of isomannide-derived ILs, as reported by Da Silva and Pereira (**A**) [65], and the one, as reported by Carcedo et al. (**B**) [67].

In a representative procedure, Da Silva and Pereira in 2011 [65] synthesized some new ILs starting from isomannide (Scheme 15A). The key step was the initial derivatization of the two hydroxyl groups as tosylates, by reacting isomannide with an excess (2.5 eq) of tosyl chloride in pyridine as the solvent. The reaction afforded a mixture of two products: The ditosylated derivative **68a** was obtained as the major product, while monotosylated **68b** was obtained in smaller amounts. The two derivatives were purified by column chromatography, and then compound **68a** was subjected to a nucleophilic substitution with a 1-alkyl imidazole to yield **69a,b**. At this point, an anion metathesis was carried out to expand the number of ionic liquids obtained (Scheme 15A).

Compound **68b** was subjected to a similar synthetic route, which featured introducing a protecting group on the free alcohol prior to nucleophilic displacement of the tosylate. A partial undesired deprotection took place during the purification of compound **71a**, and therefore, only the deprotected compounds were obtained as chemically pure (Scheme 15A).

The main drawback of this strategy is the initial derivatization: It was not possible to solely obtain the double tosylation even using an excess of TsCl, and the yield of the process was not high. Furthermore, the need to purify this product by column chromatography made the process unsuitable for scaling-up.

In a similar way, Carcedo et al. synthesized compound **69f** (Scheme 15B) [67]. The authors prepared compound **68a** reacting isomannide with a lower excess of TsCl (1.2 eq) in pyridine as the solvent, and the chemically pure product was obtained after a simple recrystallization in higher yield. Then, after a quaternization reaction with 1-methylimidazole and a final metathesis reaction, compound **69f** was obtained (Scheme 15B).

Since no column chromatography was needed to obtain the desired compound, this strategy does have some advantages over the previous one; scaling-up is possible, and this approach fits better the principles of Green Chemistry commonly associated with ionic liquids.

**Scheme 16:**
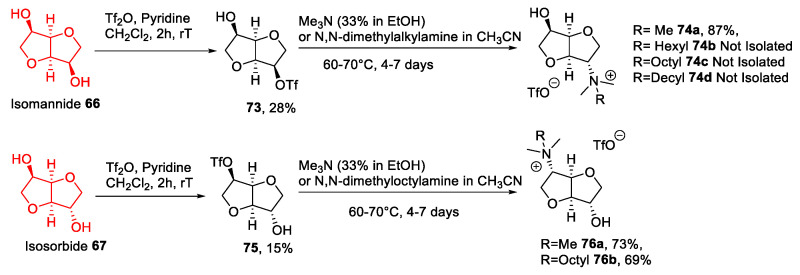
The synthesis of ILs from isomannide and isosorbide, as reported by Sikora et al. [66].

The same problems of Pereira’s procedure affected the synthesis, reported by Sikora in 2020 [66]. Starting from isomannide, the statistical monoderivatization with triflic anhydride gave a poor yield, and the product had to be purified by column chromatography (Scheme 16). Surprisingly, even starting from isosorbide, no selective derivatization was achieved; the two monotriflates were obtained in a 1:1 ratio, highlighting a very similar reactivity of the *endo* and *exo* positions in this reaction. (Scheme 16) Next, the displacement of the triflate group with a tertiary amine gave the ionic derivatives.

**Scheme 17:**
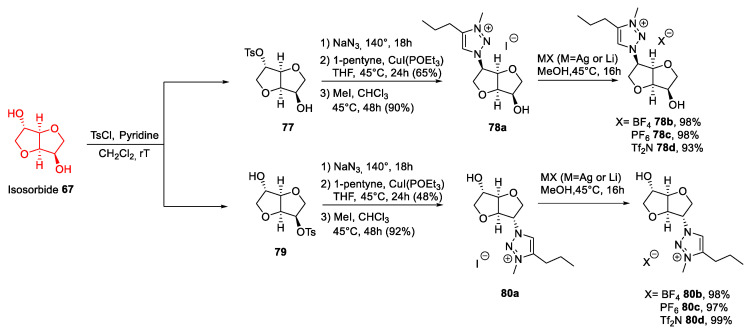
The synthesis of ILs from isosorbide, as reported by M’Sahel et al. [68].

A slightly different approach, employed by M’Sahel et al. [68], was based on introducing an azido group to build a heterocyclic compound through a Huisgen 1,3-dipolar cycloaddition (Scheme 17). A subsequent quaternization step with an alkyl iodide led to an ionic compound, which after an anion metathesis reaction gave a range of ILs. This strategy is still affected by the above-described problems: As for the reaction with triflic anhydride, no selective derivatization of isosorbide with tosyl chloride was possible. As reported previously [69], the tosylation of isosorbide led to a mixture of the *endo*-tosyl derivative **79** (45%), the *exo* derivative **77** (11%), and the ditosylated compound. As a result, the yield was modest, and a purification step was needed, making the process for obtaining these ILs scarcely green.

To summarize, the main problem encountered in the synthesis of ionic liquids from isohexides is the selective derivatization of the starting material. To expand the utility of this kind of compounds in ionic liquid synthesis, new synthetic strategies and more selective protocols need to be developed.

**Scheme 18:**
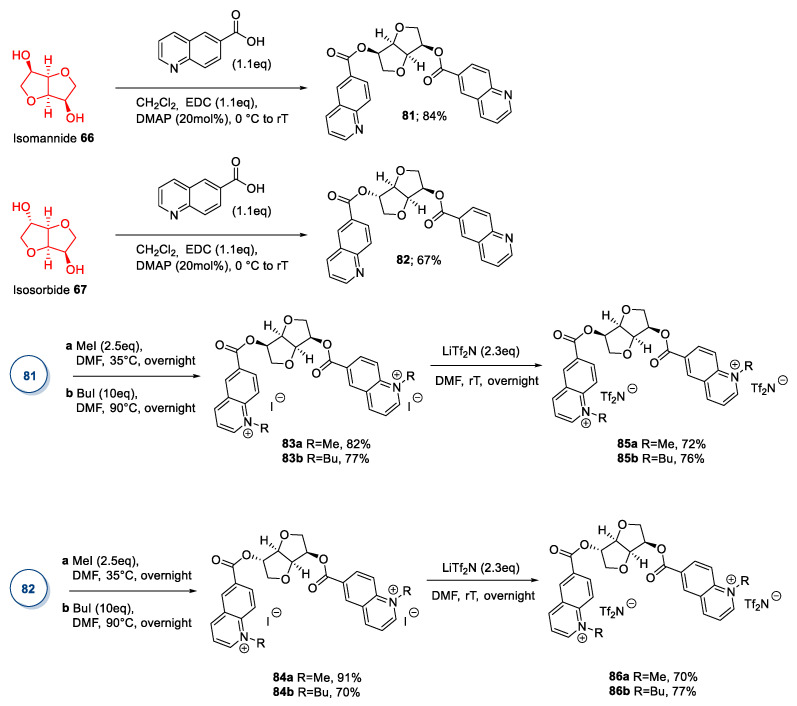
Synthesis of new ILs from isomannide and isosorbide, as reported by Zullo et al. [70].

Following this idea, in 2020, Zullo et al. [70] developed a different strategy for synthesizing new ILs starting from isomannide and isosorbide. (Scheme 18) In their work, the authors preserved the native stereochemistry of the parent sugar derivative: In particular, isomannide and isosorbide were converted into the 6-quinolinic carboxylic diesters **81** and **82** with a simple double esterification reaction in the presence of *N-*(3-dimethylaminopropyl)-*N*′-ethylcarbodiimide (EDC) and 4-dimethylamino pyridine (DMAP) (Scheme 18). The pure products were obtained in good yields after crystallization from the reaction mixture or recrystallization of the crude. Compounds **81** and **82** were then alkylated with methyl iodide or butyl iodide to yield ionic derivatives **83** and **84,** which were eventually transformed in their corresponding bis[(trifluoromethyl)sulfonyl]imide salts **85** and **86** by anion exchange (Scheme 18).

This strategy requires a lower number of synthetic steps than the other strategies reported, and no chromatographic purification is needed, leading to a ‘*greener*’ synthesis of new ILs starting from isohexides. An expansion of the explored chemical space could be easily reached by a simple change of the carboxylic acid employed and/or a change in the alkylating agent, leading to a wider number of ionic products.

### 2.6. d-glucose and *N*-methyl-glucamine

d-glucose, the most abundant monosaccharide in nature, is industrially obtained from starch through enzymatic or acidic hydrolysis [71].

**Scheme 19:**
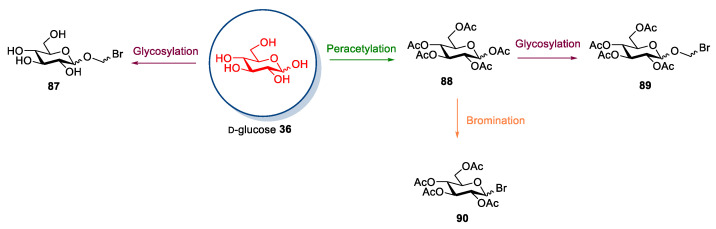
The selective derivatization of anomeric carbon in d-glucose.

Glucose is by far the most employed sugar in the synthesis of ionic liquids. To this aim, the derivatization of glucose is mainly based on a selective reaction at the anomeric position, which can be made directly on the unprotected sugar **36** or after its peracetylation (Scheme 19). The two main strategies involve: (a) The introduction of halogenated alkyl chain [32,54,72,73,74,75,76,77,78,79,80,81], and (b) the substitution of the hydroxyl group with bromine (Scheme 19) [82,83,84,85,86,87,88].

**Scheme 20:**
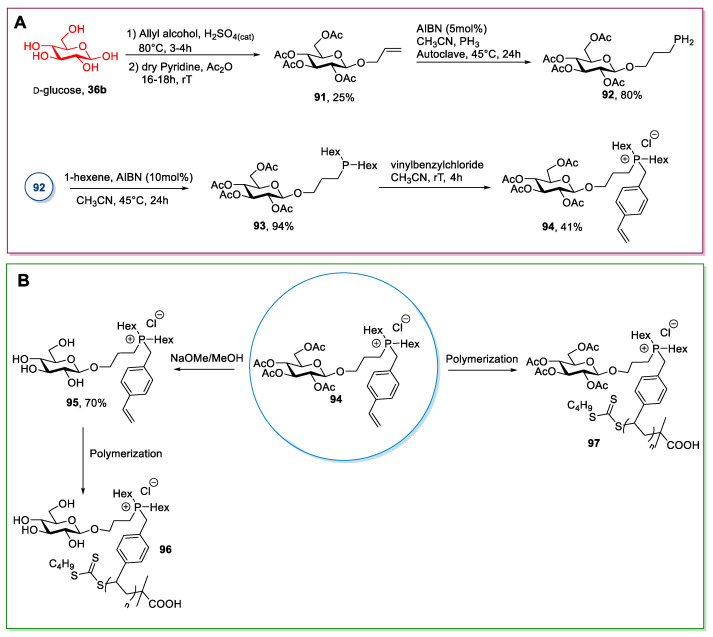
The synthesis of polymeric ILs, as reported by Cuthberth et al. [80]; the scheme reports the synthesis of the ionic monomer **94** (A) and the synthesis of polymeric ILs (B).

A slightly different approach, albeit conceptually similar, entails the introduction of an allyl substituent. This procedure was used by Cuthberth et al. in 2018 [80] for synthesizing polymeric ionic compounds (Scheme 20). In this specific case, the synthesis started with preparing peracetylated α-allyl glucoside **91** through a Fisher glycosylation of glucose followed by a peracetylation of the sugar [89,90]. After the purification of the desired anomer by column chromatography, the terminal double bond was used for further derivatizations to introduce the cationic moiety. This goal was reached through the addition of PH_3_ and subsequent double alkylation of the phosphorous atom. (Scheme 20A) The addition of PH_3_ is quite demanding: Fine-tuning of the reaction conditions was needed to have almost only primary phosphines and only traces of secondary and tertiary phosphines. Moreover, the purification step was a column chromatography under N_2_ to avoid the oxidation of the products. The ionic compounds were obtained by quaternization of the phosphorus atom with 4-vinylbenzyl chloride, which introduces the polymerizable styryl moiety. Both peracetylated **94** and deprotected **95** ionic compounds were used to prepare the corresponding polymeric ionic derivatives. (Scheme 20B) This protocol is synthetically demanding, due to the length of the synthetic route and the great number of purification steps needed.

A selective glycosylation reaction [89,91] similar to that described for d-galactose and d-xylose (see Section 2.1 and 2.2) was used to introduce a variety of mono- [32,54,72,73,74,75,76,78,81] and dihalogenated [77] alkyl chains at the anomeric position. The nucleophilic substitution of the halogen group with a tertiary amine gave the desired ionic derivative. When the starting material was the peracetylated sugar, a final deacetylation reaction to give the unprotected corresponding ionic liquid was also required.

**Figure 4:**
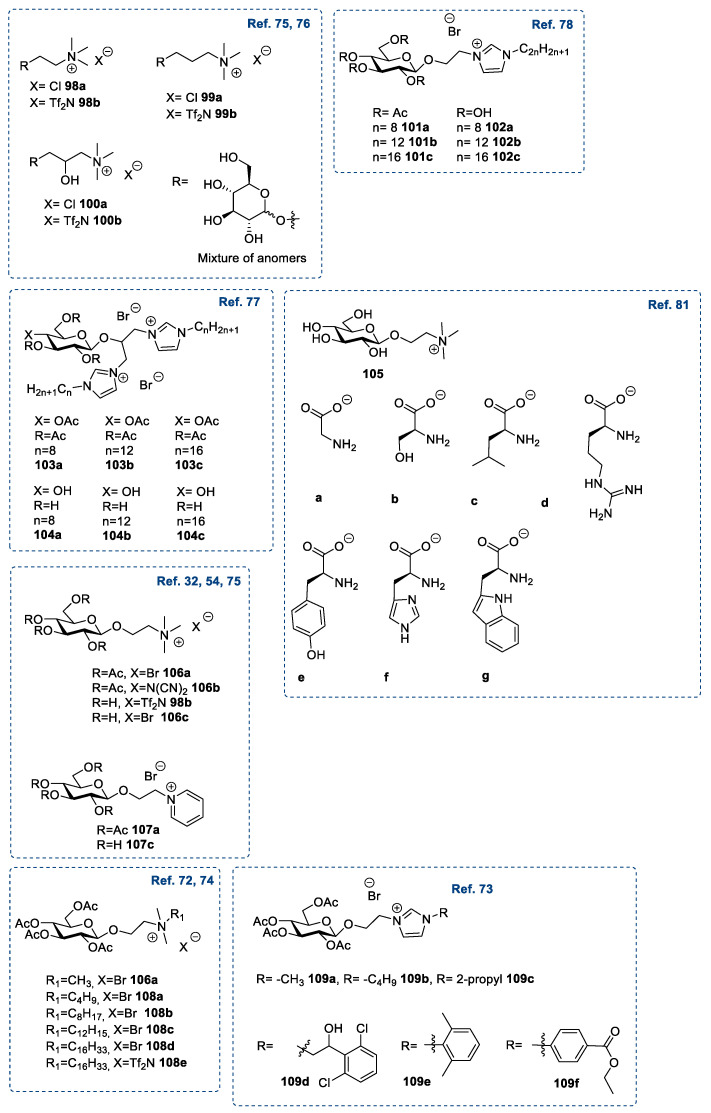
Structures of d-glucose-derived ILs [54,73,74,75,76,77,78,81].

A salt metathesis reaction was also used to expand the library of the final ILs; not only classical metathesis reactions with inorganic salts were used, but also more peculiar reactions to introduce exotic anions (herbicides [74] or amino acids [81]). Following this approach, a variety of different ILs have been synthesized starting from d-glucose (Figure 4).

**Scheme 21:**
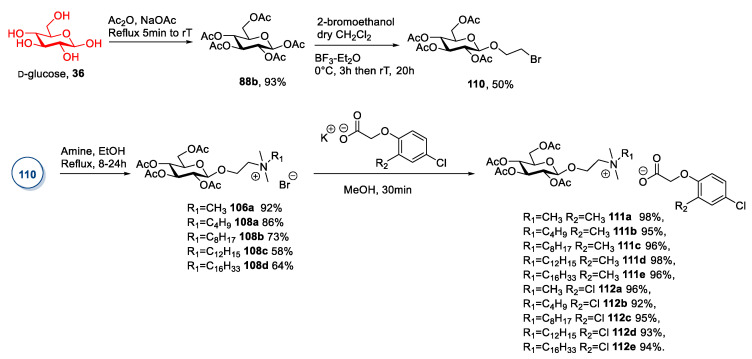
Synthesis of herbicidal ILs, as reported by Pernak et al. [74].

In a representative procedure, Pernak et al. [74] synthesized some glucose-derived ILs containing an herbicidal anion (Scheme 21). As seen before for d-galactose and d-xylose (see Section 2.1 and 2.2), the synthesis started with the peracetylation of β-d-glucose to selectively yield the β-anomeric acetate and a subsequent glycosylation with 2-bromoethanol [92]. In this case, due to the use of halogenated alcohol, trace amounts of disaccharide and trisaccharide were observed; column chromatography was needed to obtain the pure desired compound (Scheme 21). The yield of this step was moderate, given the incomplete conversion of the starting material. The 2-bromoethyl-2,3,4,6-tetra-*O*-acetyl-β-d-glucopyranoside **110** was reacted with various tertiary amines to give ionic bromides **106a** and **108**, which were purified by column chromatography. The last step was an anion metathesis with potassium salt of the herbicidal anion of interest (Scheme 21).

**Scheme 22:**
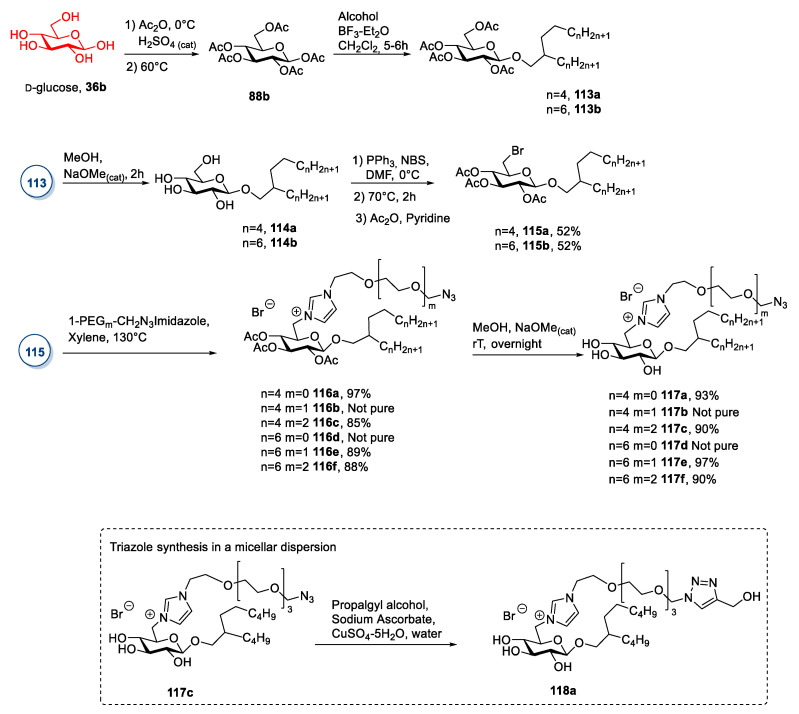
Synthesis of PEGylated ILs, as reported by Goh et al. [79].

This approach was also used by Goh et al. in 2019 [79] (Scheme 22) to introduce a branched long-chain at the anomeric position, starting from peracetylated glucose [93,94]. Then, after a deacetylation reaction, the authors took advantage of the higher reactivity of primary over secondary hydroxyl groups to perform a selective bromination at glucose’s C6, using triphenylphosphine and *N*-bromosuccinimide [95]. A peracetylation was carried out to enable easy chromatographic purification by reducing the polarity of the crude mixture. Then, an S_N_2 reaction with a nitrogen nucleophile led to the desired ionic product **117** after a final deprotection step (Scheme 22). The chemistry proposed by Goh et al. is interesting given that different selective derivatizations of the starting material can afford different ILs, either in a protected or unprotected form. However, this strategy is not optimal for the preparation of ILs, as again, different protection-deprotection steps are needed, low yielding steps are an issue, the nitrogen nucleophile has to be synthesized, and some chromatographic purifications need to be performed.

The second approach is based on the selective introduction of a bromine atom at the anomeric position of peracetylated glucose [96,97], as seen for d-galactose (see Section 2.1).

The obtained halo-sugar is used for further derivatizations through S_N_2 reactions that directly introduce the final nitrogen-containing cationic group (imidazole or pyridine) through a quaternization reaction, or a functionalized side-chain, which will be further derivatized to obtain the final ionic derivative [80,82,83,84,85,87,88].

**Scheme 23:**
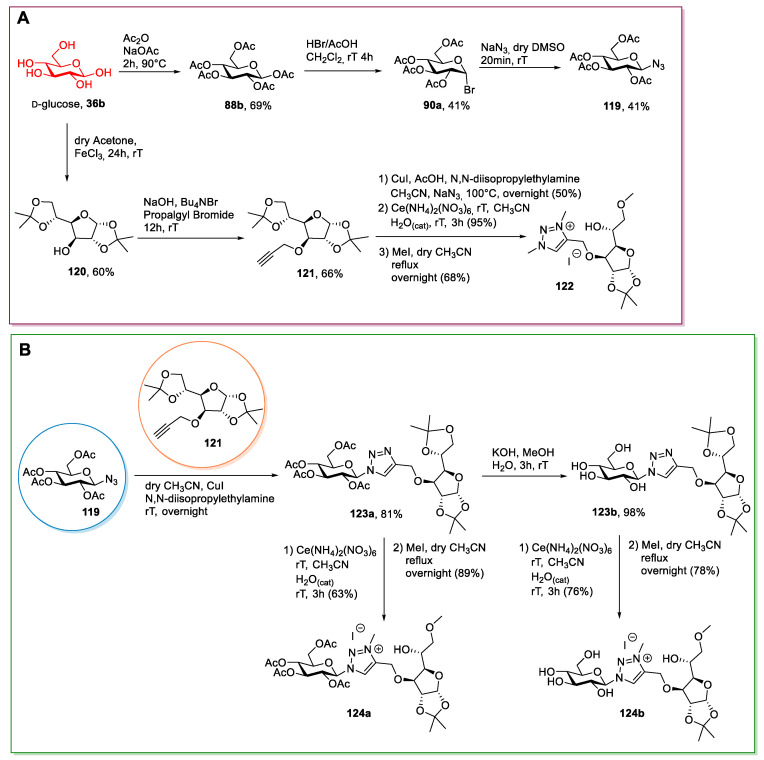
The synthesis of triazolium ILs, as reported by Jha et al. [82]. The scheme reports the synthesis of azido-glucose **119** and IL **122** (A) and the synthesis of ILs **124** starting from **119** (B).

This strategy was used by Jha et al. in 2013 [82] for synthesizing some 1,2,3-triazolinium ionic liquids from glucose (Scheme 23). After the initial bromination of the per-*O*-acetyl glucose **88b** and the S_N_2 reaction in DMSO to introduce the azido group, the first reaction was a Huisgen 1,3-dipolar cycloaddition. The authors used a d-glucose derivative in its furanosidic form as the alkyne bearing partner. The latter was obtained through a selective protection of glucose to give 1,2-5,6-di-*O*-isopropylidene-α-d-glucofuranose **120** followed by the derivatization of the free alcohol with propargyl bromide (Scheme 23A). The final ionic compound was obtained after the selective hydrolysis of one of the dioxolane rings (through a cerium catalyzed reaction) and quaternization of the triazole nitrogen with methyl iodide, with concomitant methylation of one of the free hydroxyl groups (Scheme 23B).

Alkyne **121** was also used to directly obtain a new IL through a Huisgen 1,3-dipolar cycloaddition with NaN_3_, a cerium-mediated partial deprotection of the furanosidic sugar, and a final methylation of the heterocycle with MeI. In this case, double methylation of the 1,2,3-triazole was observed (Scheme 23A). This protocol is very long and synthetically demanding: Various chromatographic purifications are needed to obtain the final desired product, and some low-yielding steps are present. Another issue is represented by the Huisgen 1,3-dipolar cycloaddition, given that an excess of the copper salt is needed to obtain good conversions.

Other works followed a similar strategy, where per-*O*-acetyl glycosyl bromide **90a** was reacted directly with pyridine [83], or with imidazole and an alkylating agent to achieve the desired ionic derivative [84,85,86,87].

**Scheme 24:**
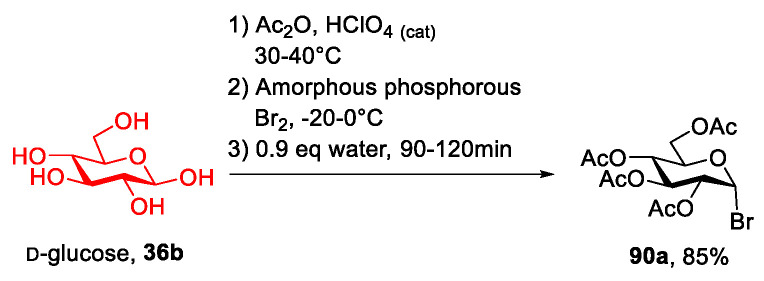
The synthesis of acetobromo d-glucose, as reported by Barczay-Martos in 1950 [98].

The key points of these strategies are the bromination reaction and the quaternization reaction. The efficiency of this latter reaction very much depends on the reagents used. Regarding the preparation of the bromo sugar, often the protocol developed by Barczay-Martos in 1950 [98] is followed. This involves first preparing the peracetylated glucose (or another sugar) with acetic anhydride and a catalytic amount of perchloric acid, then the reaction with hydrogen bromide generated in situ (Scheme 24). It worth mentioning that although the reaction gave a good yield (85%) after a simple recrystallization of the crude product, easier and especially safer protocols have been developed (for instance, see Scheme 24).

**Scheme 25:**
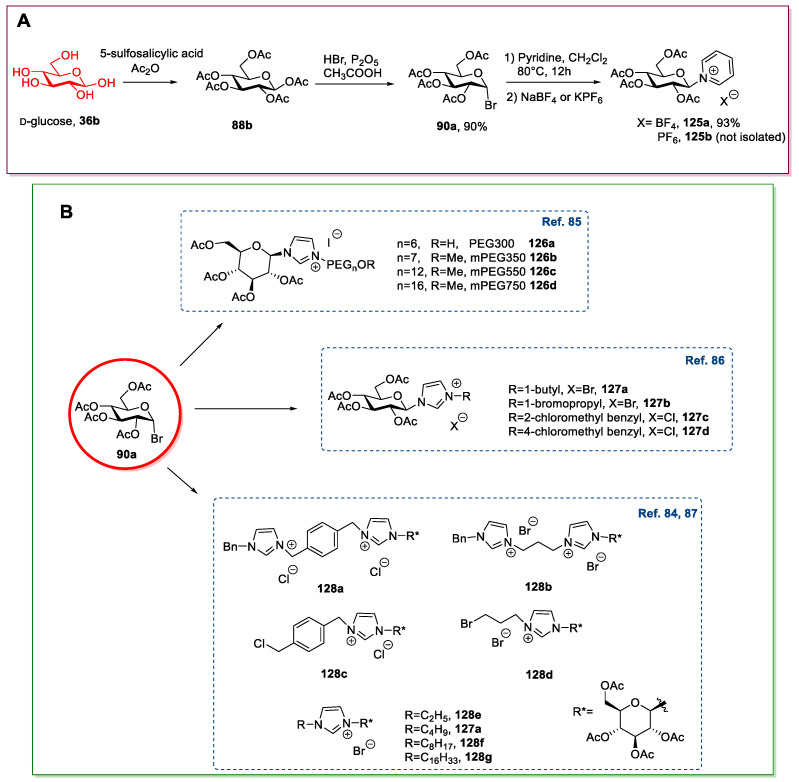
The synthesis of ILs from d-glucose, as reported by Yuan et al. [83] (**A**), and other ILs obtained from per-O-acetyl glycosylbromide **90a** (**B**) [84,85,86,87].

A very interesting protocol was developed by Yuan et al. [83] in 2017; they were able to obtain pyridine containing ILs **125** in high yield without the need of chromatographic purifications (Scheme 25A).

Other examples of ILs synthesized following a similar approach are summarized here below (Scheme 25B).

**Scheme 26:**
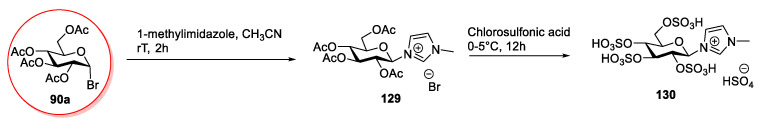
The synthesis of the Bronsted acidic IL, as reported by Zhuo et al. [88].

The Bronsted acidic ionic liquid **130** was obtained by Zhuo et al. in 2015 [88] by reacting bromide **90a** with 1-methylimidazole, followed by a reaction with chlorosulfonic acid (Scheme 26). The synthesis was very simple, and no purification was needed, despite the limited scope, due to the use of a strongly acidic reagent.

**Scheme 27:**
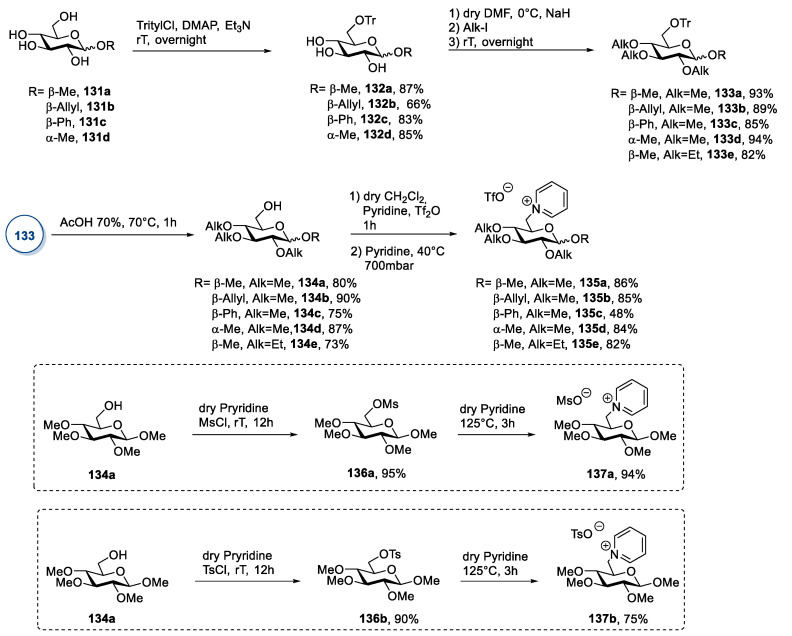
The synthesis of ILs from d-glucose, as reported by Reiβ et al. [99].

A slightly different approach, less common than the two described above, relies on the selective derivatization of the primary hydroxyl group of an alkyl glucoside [33,54]. This strategy was used by Reiβ et al. [99] in 2020 to synthesize a series of new pyridinium-containing ionic liquids, starting from commercial alkyl-β-glucopyranosides **131a–c** and methyl-α-glucopyranoside **131d** (Scheme 27).

The first step of the synthesis was the selective protection of the primary hydroxyl group, followed by methylation (or ethylation) of the remaining alcoholic groups. Deprotection of the primary position, conversion into a triflate, mesylate, or tosylate group, followed by nucleophilic displacement with pyridine, gave the ionic compounds **135** (Scheme 27).

This approach led to new ILs through a simple protocol that could be modified to introduce different alkyl chains and/or functional groups and to access a wide library of diverse compounds. The main drawback of this protocol is represented by the high number of synthetic steps (including protections and deprotections) and chromatographic purifications needed.

**Scheme 28:**
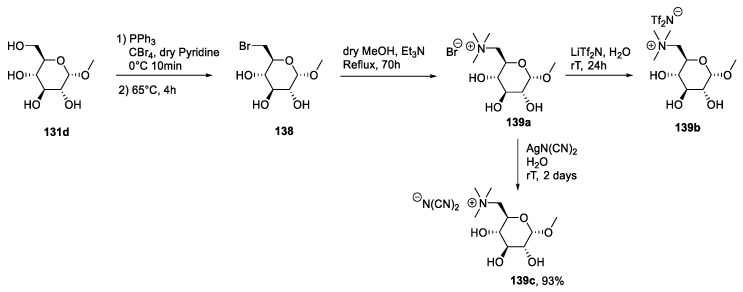
The synthesis of ILs from methyl-α-glucopyranoside **131d**, as reported by Brzeczek-Szafran et al. [54].

A simpler protocol was devised by Brzeczek-Szafran et al. in 2019 (Scheme 28) [54]. In their work, the authors synthesized a range of novel glucose-derived ILs from commercially available methyl-α-glucopyranoside **131d**. Crucially, in this case, only one intermediate (**138**) needed column chromatography purification (Scheme 28). This approach better fits in the concept of Green Chemistry connected to ionic liquids.

**Scheme 29:**
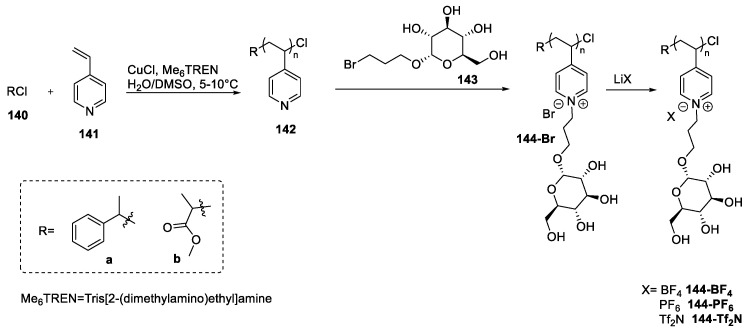
The synthesis of polyionic liquids, as reported by Chen et al. [100].

An additional approach is found in the paper of Chen et al. [100]. In their study, the authors synthesized a polyionic liquid through a post-functionalization of a pyridine-containing polymer: An S_N_2 reaction of the polymer **142** with 3-bromopropyl-α-d-glucose **143** gave the poly-IL **144-Br** that could be used to obtain more hydrophobic compounds through a salt metathesis reaction (Scheme 29).

*N*-Methylglucamine **145**, also called meglumine, has been used for preparing ionic liquids. Meglumine can be obtained directly from d-glucose through reductive amination [101] with methylamine and H_2_ at high temperature and pressure, and it is commercially available and cheap.

**Figure 5:**
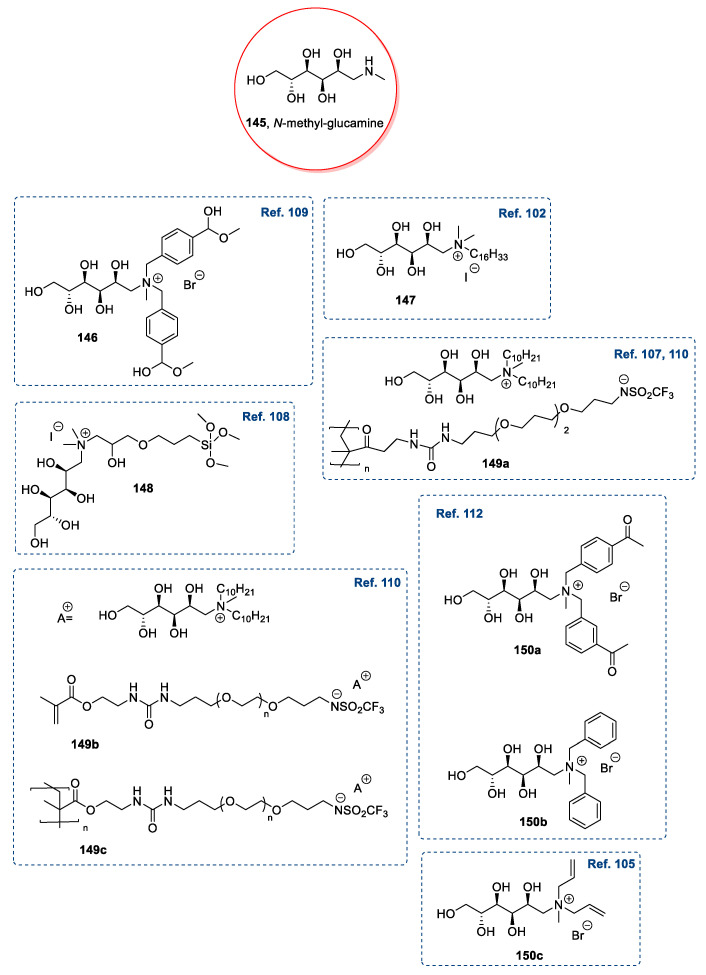
Examples of ILs obtained starting from *N*-methylglucamine **145** [102,105,107,108,109,110,112].

*N*-Methylglucamine has been widely used for synthesizing new ionic liquids [102,103,104,105,106,107,108,109,110,111,112], always through a direct quaternization of the starting material. This reaction could be performed through a one-pot double alkylation [105,109,112], a two steps double alkylation [102,103,104,106,107,108,110], or a mono alkylation followed by a neutralization reaction [106,111]. The latter approach is very simple, does not require prior protection of the free hydroxyl groups, and therefore, has been used for preparing a variety of ILs. In some cases, a final anion metathesis was performed to introduce a specific anion, and in particular, a polymeric triflimide-derived anion (Figure 5) [107,110].

**Scheme 30:**
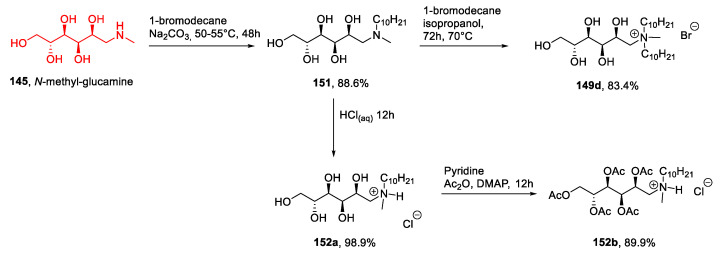
The synthesis of ILs from meglumine, as reported by Joshi et al. [103].

In a representative procedure, Joshi et al. [103] synthesized compounds **149d** and **152** starting from commercial *N*-methylglucamine (Scheme 30). The first step of the synthesis was the alkylation with 1-bromodecane; an excess of the amine was reacted in MeOH at 50–55 °C for 48 h, and the pure product **151** was obtained after recrystallization. This compound was then used to prepare compound **149d** through another reaction with 1-bromodecane and to obtain compound **152a** through a neutralization reaction with HCl_(aq)_. A final peracetylation was also carried out to give compound **152b** (Scheme 30). This synthesis was characterized by easy and fast reactions and led to the desired products without the need for long and demanding purification steps. These features are very desirable in the synthesis of ILs and also explain why meglumine **145** has been widely used in this field.

### 2.7. d-mannose

d-mannose, a C-2 epimer of d-glucose, is an important monosaccharide for human metabolism, and it is primarily found in mannan, cellulose, and hemicellulose [113]. d-mannose has not been widely used as a starting material for synthesizing ILs: From 2011 to now, only a handful of reports can be found in the literature [32,33,80,100].

**Scheme 31:**
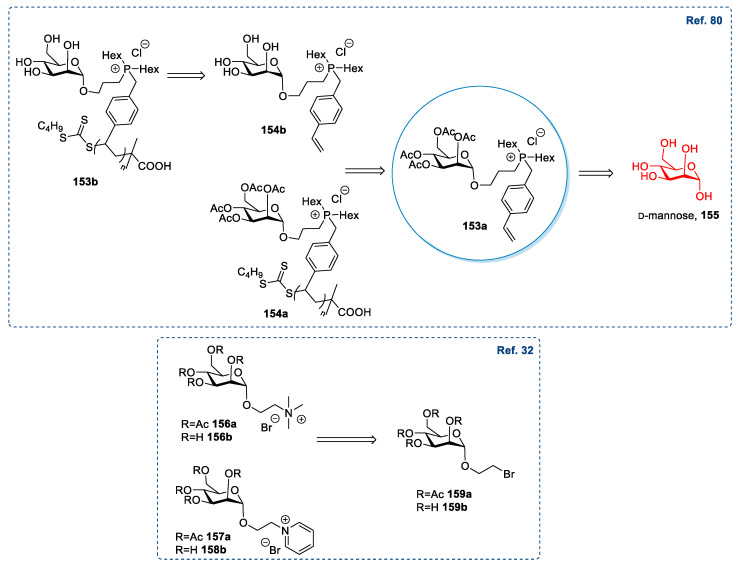
ILs derived from mannose [32,80].

The main strategy to develop new ILs from mannose relies on the glycosylation reaction, either starting from the unprotected sugar or employing its peracetylated form. This approach is the same seen for d-glucose (see Section 2.6); the reaction employs BF_3_·Et_2_O and has been used to introduce an allyl [80] or haloalkyl group [32], which, after further derivatization, gave the desired ionic compounds. These two strategies are the same described in Section 2.6 for d-glucose and present similar yields, advantages, and drawbacks (Scheme 31).

**Scheme 32:**
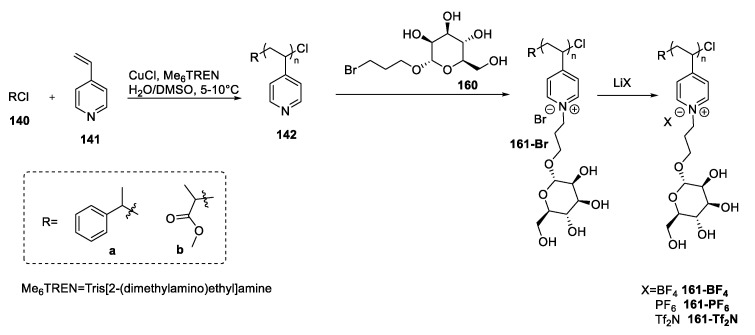
The synthesis of polyionic liquids, as reported by Chen et al. [100].

A post-functionalization of a polymer has also been carried out by Chen et al. in 2020 [100] to obtain new polyionic liquids **161** starting from a pyridine containing polymer **142** and a halo-containing mannoside **160**, with the same protocol used starting from d-glucose (see Section 2.6) (Scheme 32).

**Scheme 33:**
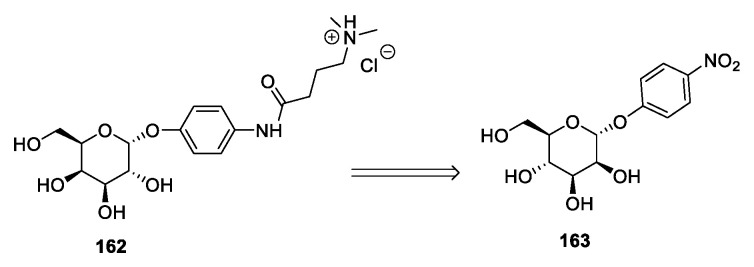
The synthesis of a new IL from d-mannose, as reported by Ahmad et al. [33].

A different approach was employed by Ahmad et al. [33] in 2015 (Scheme 33): Starting from 4-nitrophenyl-α-d-mannopyranoside **163**, the authors synthesized a new IL in a similar way to that described in Section 2.1 for d-galactose (Scheme 33). This strategy presents the same features described for d-galactose, and even the yields of the single steps are very similar.

### 2.8. Other Monosaccharides

To conclude this first part dedicated to the synthesis of ILs from carbohydrates, we have to consider some less-employed monosaccharides.

Among them, d-ribose, one of the main components of RNA that can be obtained on a large-scale by fermentation [114] of d-glucose, is the most commonly employed building block. Its use for synthesizing ILs was reported in two articles from 2011 to now [21,115]. The same strategy was followed in both papers: The initial selective protection of the secondary hydroxyl groups was followed by transforming the free primary alcohol into a leaving group. Displacement with a nitrogen-containing nucleophile gave the desired ionic compound after an S_N_2 reaction [116].

**Scheme 34:**
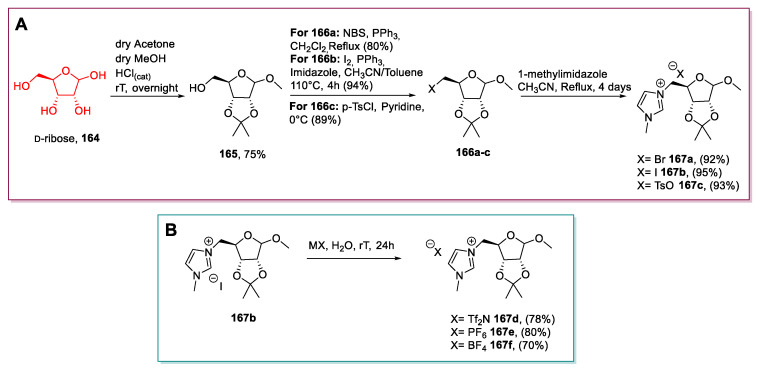
The synthesis of ribose-derived ILs (**A**) and the metatesis reaction (**B**), as reported by Jayachandra and Reddy [21].

Jayachandra and Reddy in 2015 [21] followed this approach to synthesize compounds **167**; following the initial protection, different derivatizations of the free hydroxyl group were carried out to install several leaving groups that, after reaction with 1-methylimidazole, yielded three ionic liquids with the same cationic core and different anions (Scheme 34). Compounds **167a,b** could not be classified as ILs, and the authors reported l-ribose as the sugar even if they stated that the starting material was d-ribose.

This strategy is very practical as intermediates and final products are obtained in a chemically pure form after a simple work-up or a crystallization. Further development of this strategy would be certainly welcomed as it would prepare new libraries for novel ILs.

**Scheme 35:**
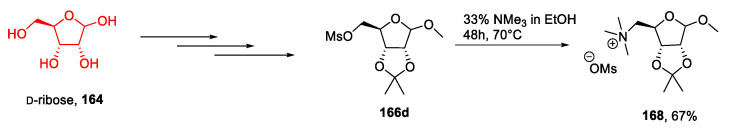
Synthesis of a d-ribose-derived IL, as reported by Dmochowska et al. [115].

A very similar strategy was used by Dmochowska et al. in 2013 [115]: Once the protected sugar **165** was obtained [116], the free hydroxyl group was converted into a mesylate group, and the final product **168** was obtained through an S_N_2 reaction with Nme_3_ (Scheme 35). This approach presents the above-discussed advantages and limitations.

**Figure 6:**
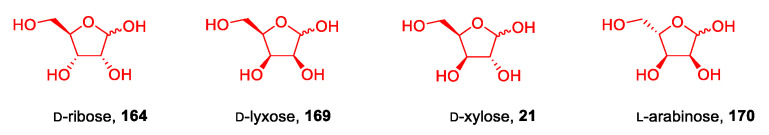
Structures of pentoses employed by Reiβ et al. in their work [99].

Following a different approach, Reiβ et al. in 2020 [99] synthesized a small library of new ionic liquids starting from peracetylated d-ribose, d-xylose, d-lyxose, and l-arabinose (Figure 6).

**Scheme 36:**
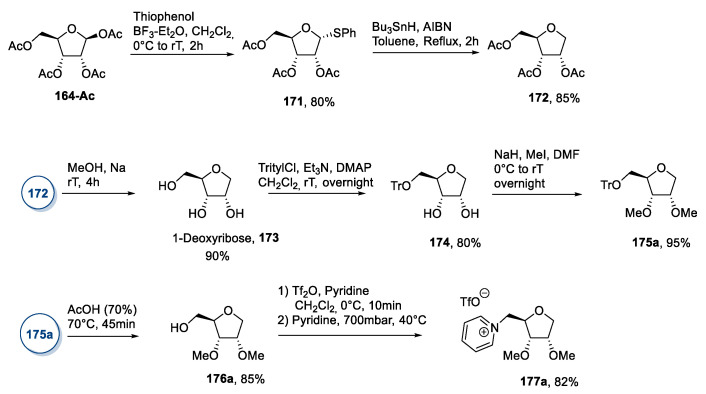
Synthesis of a new IL from d-ribose, as reported by Reiβ et al. [99].

Starting from d-ribose **164**, the authors synthesized its deoxy analog **173** through introducing a thiophenyl group at the anomeric position and a subsequent reduction with Bu_3_SnH (Scheme 36). Once the desired deoxyribose **173** was obtained, the attention moved to the selective methylation of the secondary hydroxyl groups and the installation of a leaving group at position C5. This was achieved through total deacetylation, followed by tritylation of the primary hydroxyl group to give **174**. Methylation of the free hydroxyl groups with NaH/MeI in DMF gave **175a**, which was deprotected to afford the primary alcohol **176a**. The latter was subjected to a one-pot reaction with trifluoromethanesulfonyl anhydride and pyridine to give the final desired compound **177a** (Scheme 36).

**Scheme 37:**
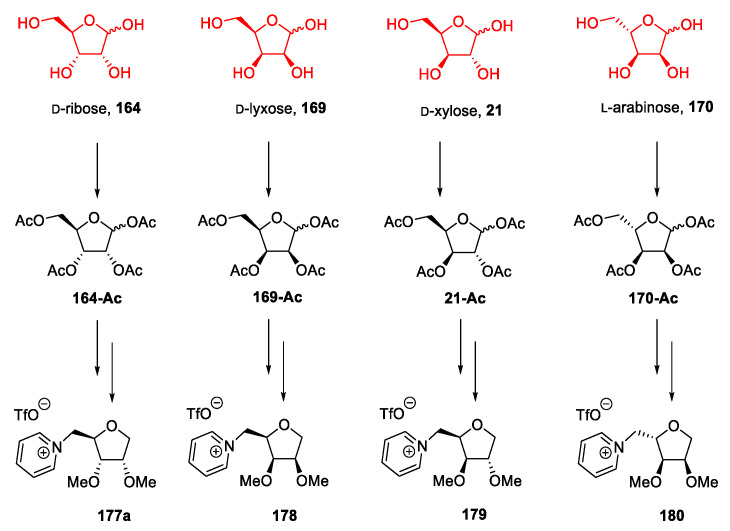
Structures of ILs obtained from pentoses and their parent sugar, as reported by Reiβ et al. [99].

The same strategy was also pursued starting from peracetylated d-xylose, d-lyxose, and l-arabinose to obtain the corresponding ionic liquids **178**, **179**, **180** (Scheme 37).

**Scheme 38:**
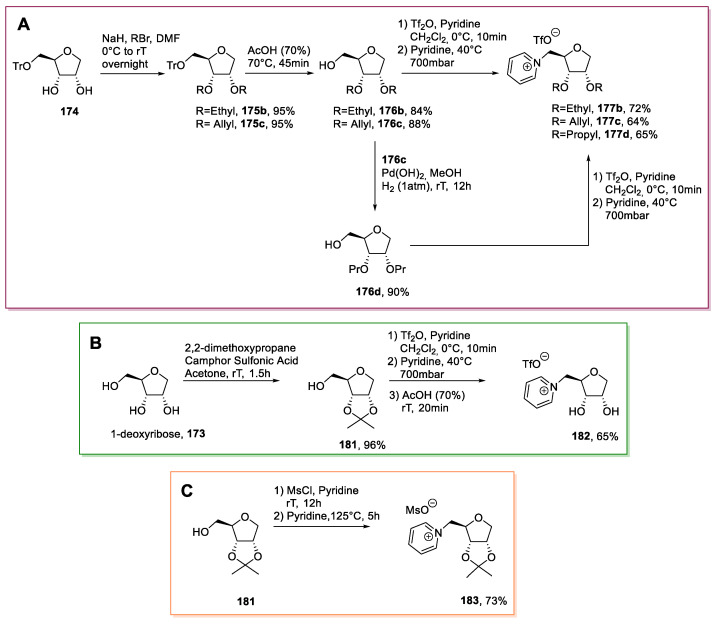
Synthesis of ILs from d-deoxyribose (**A** and **B**) and protected d-deoxyribose (**C**), as reported by Reiβ et al. [99].

A slightly different approach was followed, starting again from d-ribose **164** to obtain different deoxyribose-derived ILs (Scheme 38).

In particular, the use of different alkylation reactions at the free secondary hydroxyl groups, or their protection with 2,2-dimetoxypropane, were carried out to obtain, after the final deprotection, ILs with a diverse range of substituents at the sugar’s C2/C3 positions. In the first case, protected deoxyribose **174** was reacted with an alkyl bromide, and the primary hydroxyl group was deprotected to give compounds **176**. These were reacted with Tf_2_O to yield the correspondent triflates, which were transformed into ILs **177**, after reaction with pyridine (Scheme 38A). These transformations were also performed to obtain compound **182**, which was prepared from triol **173** via protection with 2,2-dimethoxypropane, activation of the primary position, and pyridine displacement to give the desired pyridinium salt **182** (Scheme 38B). A similar protocol was also employed to obtain the protected IL **183** with a methanesulfonyl anion (Scheme 38C).

This work is relevant as it illustrates a general procedure for obtaining a diverse range of ILs, starting from pentose cores, characterized by different stereochemistry. Furthermore, with minimal modifications, this approach could be employed to introduce a variety of functional groups in the final compound. As a result, it is possible to study the influence of different structural variations on the physicochemical properties of the final ILs. This is indeed a very much sought-after goal in ionic liquids research, as the possibility of tuning ILs’s features to their final applications is indeed very intriguing. To this aim, the work of Reiβ is promising, since a wide range of compounds can be obtained following the same synthetic route. The main drawbacks of this approach are the length of the synthetic route, as well as the need to purify all the intermediates via column chromatography, so that the *greenness* linked to the concept of ionic liquids is missing.

### 2.9. Disaccharides

As just described in the previous sections, a great number of different ionic liquids have been synthesized in the last few years, starting from monosaccharides, after the development of different new synthetic routes. Beside chromatographic purifications and the length of the synthetic routes, one of the main issues associated with these strategies is the selective derivatization of one functional group over the others.

When considering disaccharides, sugars obtained from the condensation of two different molecules of monosaccharides, only very few reports have been published in the time frame considered in this literature survey. This can be mainly ascribed to the aggravated problem of chemo- and regioselectivity in the manipulation of more hydroxyl groups.

**Scheme 39:**
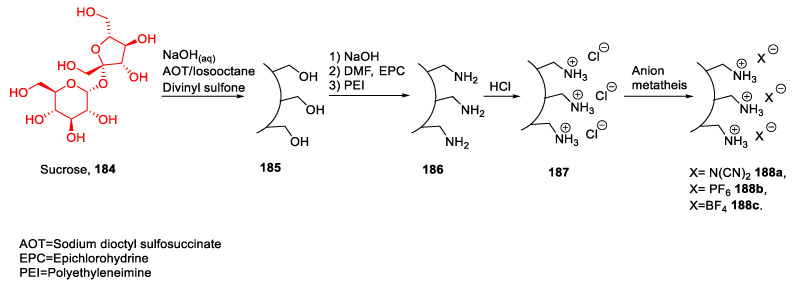
The synthesis of ionic liquid colloidal microgels, as reported by Sahiner et al. [117].

Sucrose, the common sugar used in foodstuff, is a disaccharide containing a glycosyl bond between C_1_ of a molecule of α-d-glucopyranose and C_2_ of a molecule of β-d-fructofuranose. This sugar has been used by Sahiner et al. in 2018 [117] to synthesize ionic liquid colloidal microgels (Scheme 39). The authors started their work with the synthesis of sucrose microgel **185** through a cross-linking reaction of sucrose molecules; then, they functionalized this microgel transforming alcohols into amino groups. A final neutralization reaction with hydrochloric acid led to an ionic microgel **187,** which was employed as starting material to obtain other more hydrophobic ionic gels through a metathesis reaction. (Scheme 39).

**Scheme 40:**
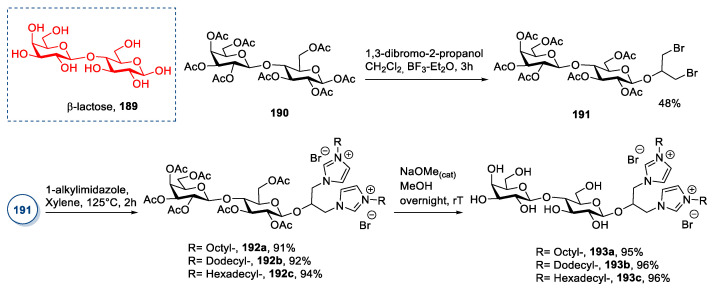
The synthesis of new ILs from lactose, as reported by Salman et al. [77].

A more “classical” synthesis was reported by Salman et al. in 2016 (Scheme 40) [77], employing lactose as starting material, a disaccharide characterized by a glycosyl linkage between C_4_ of d-glucopyranose (α or β) and C_1_ of β-d-galactopyranose. In this study, lactose, which is sourced industrially as a byproduct of dairy manufacture [20], was derivatized at the reducing end. Initially, β-lactose octaacetate **190** was reacted with 1,3-dibromo-2-propanol in the presence of BF_3_·Et_2_O to obtain the dihaloalkyl glycoside. The latter was then reacted in an S_N_2 reaction with an alkylimidazole to obtain the ionic compounds **192a–c**. These compounds were finally deacetylated to yield the more hydrophilic free sugar ionic liquids **193a–c** (Scheme 40).

This strategy is simple and relies on the glycosylation of the disaccharide, which allows for the selective functionalization of this complex starting material. This reaction sequence can also be applied to other oligosaccharides characterized by a single reactive anomeric position. In principle, this approach is suited for synthesizing a wide range of ILs and can afford final products where the central sugar core is either protected or in its natural, deprotected form. The main disadvantage of this synthesis, which can limit the application to other oligosaccharides, is the need to purify the haloalkyl glycoside by column chromatography.

Another interesting disaccharide is represented by xyloside **194**. As previously stated, (Section 2.2), xylans are the main constituents of plants hemicellulose and xylose can be derived from them [35].

**Scheme 41:**
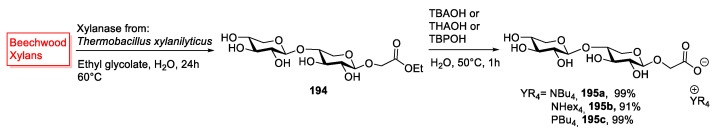
The synthesis of ILs from xylanes, as reported by Gatard et al. [38].

In 2017, Gatard et al. [38] synthesized a small collection of novel ILs starting from a xyloside containing a glycoside linkage between C_1_ of β-xylopyranose and C_4_ of another unit of β-xylopyranose (Scheme 41). The authors started from beechwood xylans, and through an enzymatic transglycosylation, they obtained a mixture of two xylosides esters, with the ester group selectively linked to the anomeric carbon of one unit of β-xylopyranose. The product of interest was then purified and hydrolyzed, using a quaternary ammonium or phosphonium salt, to yield new disaccharidic ILs **195** (Scheme 41). This strategy is particularly interesting because it uses a chemo-enzymatic approach to prepare new ionic liquids starting from biomasses. This approach combines a few important key features of Green Chemistry, such as the synthesis of ionic liquids (as new, alternative solvents), the use of enzymes as catalysts, and the valorization of biomasses.

## 3. Thermal Analysis

In the next sections, the thermal properties reported in the literature for some of the previously cited ILs will be discussed. When dealing with ILs, liquid range and thermal stability are fundamental properties to determine their potential scope of application.

The two main techniques employed were thermogravimetric analysis (TGA) and differential scanning calorimetry (DSC).

TGA is often used to assess the short-term thermal stability of an IL; the compound is placed in a crucible (made of Pt or Al_2_O_3_), and its temperature increased at a constant rate under a gas flow (inert gases such as Ar, N_2_, He, or air). The decomposition temperature can be extrapolated in different ways: The onset temperature (T_onset_), the temperature of 5% (T_d5%_), 10% (T_d10%_) or 50% (T_d50%_) weight loss and the temperature of maximal decomposition (T_max_). Variation of the instrumental parameters affects the final results: For example, higher thermal stability can be expected using an inert gas (Ar, He, or N_2_) instead of air.

DSC is employed to get insights into the melting, crystallization, and glass-transition of a solid chemical species. The compound is located in a pan (e.g., aluminum pan) and subjected to heating-cooling cycles in a temperature range in which it does not decompose. The thermogram registers the heat flow as a function of the temperature, and it can be used to determine exothermic or endothermic phenomena. As for TGA, differences in the analysis set-up influence the final results: The mass of the sample, the heating and cooling rates, and the type of pan (e.g., hermetically sealed, with a lid on the cap). The temperatures of interest are often the onset of the endo-exothermic peak (for melting and crystallization) or the inflection point of a sigmoidal transition for T_g_.

This section on the thermal analysis of sugar-derived ILs, which appeared in the literature since 2011, is divided into subsections relating to different sugar cores, followed by a final general discussion.

The different instrumental parameters employed for each measurement are reported as described in the original paper.

### 3.1. d-galactose

The thermal data on d-galactose-derived ILs are reported in Table 1.

The thermal analysis of compounds **4** and **5a** was carried out by Jayachandra et al. [23]. The melting point, measured through a digital melting point apparatus, revealed that these compounds cannot be considered ILs.

The thermogram of compound 4 showed two endothermic peaks; the first one (Tonset = 202.9 °C) is attributable to the fusion of the salt, and the second one (T_max_ = 261.0 °C) to its decomposition. In a similar way, compound **5a** displayed two endothermic peaks, which again could be ascribed to fusion (Tonset = 136.5 °C) and decomposition (T_max_ = 266.7 °C). In the original paper, the authors reported a different interpretation of the thermograms (please refer to the Appendix A of the original paper).

Kaur et al. [24] reported the thermal analysis of compounds **6** and **7a–e**. In their work, the authors employed a digital melting point apparatus to measure the decomposition temperature of each product.

From the data reported in Table 1, we can conclude that the use of 1-methylimidazole for the quaternization reaction affords more stable ILs than when DABCO is used.

For the DABCO series, varying the anion caused the thermal stability to increase in the order CF_3_SO_3_^−^ < I^−^ <BrCH_2_CH_2_SO_3_^−^ < SbF_6_^−^ < BF_4_^−^ < PF_6_^−^, with the hexafluorophosphate derivative **7b** being the most stable.

### 3.2. d-xylose and Xylosides

The thermal data and structures of d-xylose and xyloside-derived ILs, are listed in Table 2.

The thermal analysis of compounds **25a,b** was performed by Ferlin et al. in 2013 [34]. In their TGA and DSC analysis work, the authors measured T_d_ and T_g_, respectively, for each compound. The values reported were the onset temperature of the mass loss for T_d_ and the onset temperature of the glass transition for T_g_.

The products were characterized by similar glass transition temperature (4 and 2.7 °C) and a rather low decomposition temperature (120 and 150 °C), an unusual behavior that could be ascribed to the presence of the sugar moiety in the IL [55].

The decomposition of each IL was studied using a TGA-MS instrument. Each salt presented an initial constant loss of water between room temperature and 110 °C (1.33% for **25a** and 0.76% for **25b**) followed by a further step of loss of water (3.20% for **25a**, 3.01% for **25b**), due to the elimination of hydroxyl groups. The thermal degradation occurring during the second step was related to the decomposition of the BF_4_ anion with the loss of F (*m*/*z* = 19), as revealed by MS analysis.

The thermal analysis of compounds **26**, **27a–c**, **28a–c**, **29a–c**, and **195a–c** was published by Gatard et al. [38]. All the phosphonium salts were liquid at room temperature, while various ammonium ILs were solids at ambient conditions. The lowering of the melting point (by replacing a nitrogen atom with a phosphorus one) was observed in works by other groups [56,118,119]. An explanation for this phenomenon can be found in the increase of the atomic radius moving from nitrogen to phosphorus, which causes a lowering in the lattice energy and gives more flexibility to the structure. When keeping the linker between the sugar frame and the carboxylate group unvaried, increasing the length of the alkyl chain on the ammonium cation led to slightly higher melting temperatures [120]. Another feature that influences the properties of the products at room temperature was the hydrophilic-lipophilic balance of the anion; compounds containing a triazole or a pentyl group as the linker were very viscous liquids, while ionic products containing methylene or benzyl residues as the linker were solids. Moving from methyl to benzyl as the linker caused an increase of the melting point; this could be ascribed to π-π stacking interactions between the phenyl rings that led to higher lattice energy. Increasing the number of sugar units from one to two did not cause any dramatic change, and only for compounds containing the tetrahexylammonium cation, the introduction of a second carbohydrate ring increased the melting point. The glass transition temperatures, which indicate the lower limit of the interval in which the IL could be used as a liquid, are in the −35 °C to 2 °C range. No clear trend was observed for the T_g_.

The TGA analysis showed that for all the compounds obtained in this paper, the T_d_ varied between 140 and 260 °C. No clear definition of the T_d_ was given by the authors, and only an interval of temperatures was reported.

The authors found that the thermal stability was limited by the strength of heteroatom-carbon and heteroatom-hydrogen bonds [121], that phosphonium ILs were more stable than ammonium salts [122], that increasing the number of sugar units determined lower T_d_, and finally, that no particular trend was observed when the linker or the length of the alkyl chain was altered within the ammonium cation.

The two sets of data were obtained employing different instrumental parameters, and as a consequence, no meaningful comparison can be made. However, even if there is a significant difference in the structures of compounds **25**, which contains a sugar unit in the cation, and compounds **26**–**28**, which bears the saccharide in the anion, the decomposition temperatures are quite similar, with compounds **25** having a T_d_ near the lower limit registered for **26**–**29**. It is reasonable to assume that the main structural feature influencing the thermal properties of each compound is the sugar unit. Furthermore, the presence of free hydroxyl groups could be the main structural feature that lowers T_d_, due to the easy elimination of water upon heating.

T_g_ are still similar, with compounds **25** having higher T_g_.

### 3.3. d-gluconic Acid

**Figure 7:**
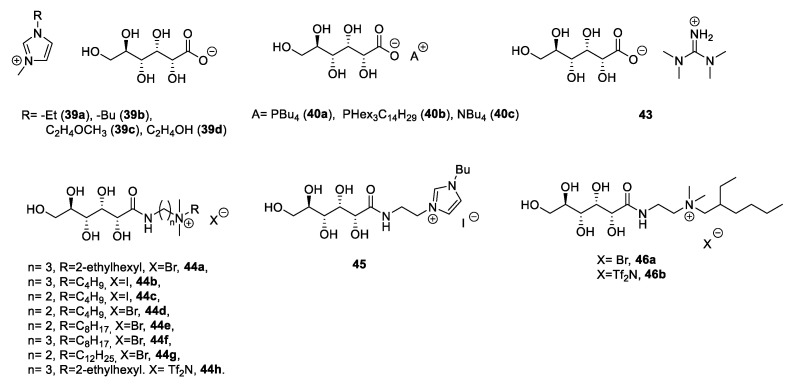
Structures of d-gluconic acid derived ILs.

The thermal data regarding ILs based on d-gluconic acid, are summarized in Table 3, and the structure of the studied compounds are depicted in Figure 7.

Costa et al. described the thermal analysis of compounds **39** [43]. In their work, they measured the T_d_ as the inflection point of the corresponding peak in DSC. All the analyzed compounds were liquids at room temperature. The authors did not report a precise T_d_ for each compound, but they only verified that the gluconate salts containing an organic cation had a lower melting point and higher thermal stability with respect to gluconic acid (T_m_ = 131 °C; T_d_ = 200 °C) and sodium gluconate (T_m_ = 200 °C, T_d_ = 200 °C).

A more detailed analysis was reported by Billeci et al. [17], in which a series of ILs derived from gluconic acid, including compounds containing gluconate salts and gluconamides, were analyzed and compared with previous data published by the same research group [44,48].

As the same protocol was followed for the thermal analysis of ILs, a comparison of their thermal stability was possible in this case. In the case of gluconamide-based ILs, the decomposition temperature was observed between 127.8 and 210.3 °C. Assessing the factors that influence the decomposition temperature, the authors reported that different trends were observed depending on the alkyl spacer, the nature of the anion, and the length of the alkyl chain on the ammonium head. For 2-amino derivatives **44a–c**, **44h**, and **46a,b**, T_d_ increased with the length of the alkyl spacer when the anion was bromide, while it decreased in other cases. The major changes in T_d_ were observed moving from a halide anion to bistriflimide anion; this effect has already been reported in the literature [123,124]. Furthermore, a comparison between the T_d_ of **44c** and **44d** showed an increase in the thermal stability on decreasing the nucleophilicity of the anion [125]. The introduction of a branched alkyl chain led to a decrease in the thermal stability, regardless of the spacer length (compare **44a** with **44e** and **44a** with **44f**). On the other hand, when varying the alkyl chain on the ammonium head, the best results were obtained in the order butyl<branched octyl<linear octyl (**44d** < **46a** < **44e**). Finally, the data showed that the thermal stability increased when moving from ammonium to imidazolium (compare **45** and **44b**), as already reported in the literature [126]. The comparison between T_d5%_ of [N_112GlyA8_]Br (130.7 °C), [N_112HexA_]Br (153.5 °C), respectively containing glycolic acid (GlyA) and hexanoic unit (HexA) in the cation, and **46a** showed that introducing the saccharide’s highly polar chain enhanced the thermal stability.

As for the gluconate-based ILs, their T_d_ were between 103.1 °C and 172.5 °C. The data collected showed that the most stable IL was the system containing a trihexyl-tetradecylphosphonium cation (**40b**), while the least stable was the IL featuring a tetramethylguanidinium portion (**43**). The decomposition temperature depended on the cation, with the ammonium-containing compounds generally more stable than the phosphonium containing ones. For the phosphonium ILs, the thermal stability increased with the length of the alkyl chain, as noted by others [123].

The DSC analysis showed a glass transition in the heating cycle, suggesting the low tendency for these ILs to crystallize [127,128]. Almost all the synthesized compounds were characterized by T_g_ < 100 °C and could be classified as ILs, with the only exception of **40c**. For gluconamide-based ILs, T_g_ ranged from −55.5 °C to −0.1 °C, and it was influenced by the anion nature and the length of the alkyl chain (i.e., for butyl derivatives **44c** and **44d** T_g_ increases from Br^−^ to I^−^, while for iodide salts, T_g_ decreases with the lengthening of the alkyl spacer). Considering the same spacer and varying the length of the alkyl chain on the ammonium head, the authors observed an initial decrease of T_g_ followed by an increase of glass-transition temperature (compounds **44d**, **44e**, **44g**), as a result of the balance between conformational freedom of the chain and van der Waals interactions [129]. The branching of the alkyl chain led to an increase of T_g_, independently of the alkyl chain length. A more unusual and complex trend was observed when varying the length of the spacer; for bromide salts, T_g_ increased with the length of the alkyl chain on the ammonium head, for Tf_2_N^−^ salts, no significant change was detected, and for iodide salts, lower values were recorded. Comparing the results obtained for [N_112GlyA8_]Br (−25.5 °C), [N_112HexA8_]Br (−27.7 °C) and **46a**, lower T_g_ were observed with a decreasing number of hydroxyl groups in the amide chain. This effect could be explained by the increase in flexibility, resulting from a reduced number of hydrogen bonds. T_g_ values decreased when the cation was changed from imidazolium to ammonium.

For gluconate-based ILs, generally higher T_g_ were observed (from −24.1 °C to 120 °C). For these compounds, the nature of the cation strongly influenced T_g_ (moving from ammonium to phosphonium), and for phosphonium salts, T_g_ increased with the length of the alkyl chain. Compound **40a** showed two glass transitions (−21.8 °C and 80.2 °C).

When giving a closer look at the thermal analysis of compounds **40**, a previous work of Billeci et al. should also be highlighted [44]. For all these compounds, a melting event was observed in the heating cycle, while a crystallization was observed in the cooling cycle. These phase transitions were completely reproducible over different cycles. The introduction of a gluconate anion led to a large increase in the T_m_, as a consequence of the increase in the ability to form hydrogen bonds, if compared with their respective chloride salts [130,131]. The melting temperature decreased moving from ammonium to phosphonium salts, and only phosphonium salts could be defined as ILs [132]. The analysis of the thermodynamic parameters showed that this change in behavior could be ascribed to the strength of anion-cation interaction rather than on the crystal lattice arrangement (greater variations in ΔH_m_ rather than in ΔS_m_ were observed). Salts containing a shorter and more symmetrical alkyl chain had higher T_m_, due to a lower grade of organization in the solid structure. This phenomenon is, in turn, a consequence of weaker cation-anion interactions in the presence of longer alkyl chains. The phase change during the melting event was also studied using a Polarized Optical Microscope (POM). An isotropic melting was always obtained, but unexpected textures were observed during the heating and cooling cycles. For example, **40c** was obtained as a crystalline solid but, upon cooling, the isotropic liquid gave rise to spherulitic structures from 115.8 °C to 36 °C. Similar behavior was observed for compound **40a**. These particular phase transitions were reported previously for *N*-alkylcaprolactame-containing ILs [133]. When the length of the alkyl chains was increased, the phenomenon was reduced as in the case of IL **40b**.

A final comparison of all the data in Table 3 shows that compounds **39** are by far the most stable ILs derived from d-gluconic acid. These compounds have a T_d_ at least 60 °C higher than the corresponding ammonium or phosphonium derivatives **40**. However, it is worth stressing that a different heating rate was used: 10 °C/min for **39** vs. 5° C/min for **40**. This can lead to an overestimation of the decomposition temperature in the case of **39**. Overall, the available data suggest that the cation plays a fundamental role for this series of ILs in determining their thermal stability.

### 3.4. d-glucuronic-, d-galacturonic- and d-quinic Acid

The thermal data regarding d-glucuronic-, d-galacturonic- and d-quinic acid-derived ILs are reported in Table 4.

Brzeczek-Safran et al. performed a thermal analysis of some ILs in 2019 [54]. In this work, compounds **52a** and **52c** were the main focus of their studies. The conversion of the parent sugars in ILs and salts enhanced their thermal stability [134]. While the imidazolium containing **52c** was an RTIL (room temperature ionic liquid), the ammonium salt **52a** was obtained as a solid with a melting point of 142–143 °C, and therefore, cannot be classified as an IL. The authors analyzed the salts by TGA, focusing their attention to the weight loss of the sample, without considering the decomposition temperature. Indeed, the aim of their work was to employ new biomass-derived ILs to obtain nitrogen-doped porous carbon materials. Their intent was to identify which IL gave the highest weight of carbonized material, regardless of its stability. Comparing compounds **52** with other glucose-derived ILs containing a sugar moiety in the cation part, they noted that the latter were more stable, possibly due to the decarboxylation of glucuronate salts [135]. Focusing on the weight loss, the authors saw that this was lower for **52c** than for **52a**, a finding that can be rationalized considering the completely aliphatic structure of the ammonium cation and the presence of more alkyl chains [136]. From the reported thermograms, it can be seen that the T_d_ is near to 200 °C and the imidazolium salt **52c** seems more stable than the ammonium derivative **52a**. Compound **52a** shows two degradation steps between 100 °C and 200 °C; the same behavior is also observed for **52c,** which showed a higher temperature for the second step and a lower temperature for the first one.

In another work by Ferlin et al. [55], compounds **52a** and **53a** were studied. The results showed that the stereochemistry of the sugar had a significant impact on the thermal properties, with the glucuronate derivative **52a** having a T_g_ higher than the galacturonate derivative **53a**; the change in the absolute configuration of a single hydroxyl group led to a ΔT of 38.8 °C. It is to note that the two uronic acids used for synthesizing **52a** and **53a** were a mixture of two anomers, and no information on the anomeric composition, which could affect the thermal properties, was given. Interestingly, the data collected by Brzeczek-Safran and by Ferlin regarding compound **52a** do not match. For this compound, a very different T_m_ was reported (Table 4, ΔT = 47−48 °C), and only in one case, **52a** could be defined as an IL.

In Ferlin’s work, the decomposition temperature was reported for compound **52a** (T_d_ = 136 °C), while for **53a**, the authors only said that T_d_ was low. The low thermal stability of these ILs was ascribed to the strength of heteroatom-heteroatom associations and carbon-hydrogen bonds [121].

In 2016, Hayouni et al. published the thermal analysis of phosphonium containing glucurononate- and galacturonate-derived ILs [56]. In their work, the authors synthesized the phosphonium analogs of ammonium ILs **52a** and **53a**. They noticed that, even if in the literature phosphonium ILs are generally described as more stable than the corresponding ammonium or imidazolium salts [122], for compounds **52b** and **53b**, the reverse was true. The low thermal stability of compounds **52b** and **53b** was attributed, as for **52a** and **53a**, to the strength of heteroatom-heteroatom associations and carbon-hydrogen bonds [121]. The T_d_ was not clearly shown, but it can be assumed that it has been measured as reported before [55]. Furthermore, the synthesized salts were both RTILs, and they also displayed a T_g_ < 0 °C and lower than that of the corresponding ammonium derivatives. The stereochemistry still had its importance, with galacturonate IL being more stable and again showing a higher T_g_ (ΔT = 13.8 °C).

Another paper reporting the thermal analysis of sugar-based ILs was published by Mondal et al. [57]. In their work, the authors analyzed some bio-based ILs, among which cholinium galacturonate **53c** and cholinium quinate **56**. Cholinium quinate showed a lower T_g_ and T_m_. Compound **53c** could be classified as an IL, while **56** was an RTIL. Compound **56** also had higher thermal stability (ΔT = 85 °C), the highest of the ILs reported in the paper.

To conclude, a comparison of the collected data clearly shows that the use of a carba-analog of a saccharide leads to higher thermal stability, as in the case of compound **56**. Furthermore, the cholinium ion seems to confer a greater stability to ILs when used as the cation and performs better than tetraalkylammonium, phosphonium, and imidazolium, particularly in the case of glucuronate salts. This constitutes a very important finding and warrants more investigation on the decomposition mechanism of the cited ILs to further understand the origin of these phenomena. Galacturonate derivatives seem to be slightly more stable than glucuronate, with the stereochemistry of C-4 being probably the cause. For the compounds analyzed in this section, the order of stability seems to be phosphonium < ammonium for galacturonate derivatives and phosphonium < ammonium < imidazolium < cholinium for glucuronate ILs.

### 3.5. Isohexides

Since 2011, the thermal analysis of isohexide-derived ILs was reported only in two works [68,70]. In the first one [68], M’Sahel et al. analyzed some triazolium containing RTILs possessing the stereochemistry of isomannide or isoidide.

**Figure 8:**
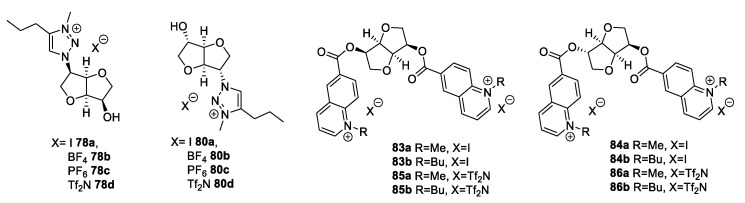
Structures of isohexide-derived ILs, as reported by M’Sahel et al. [68] and Zullo et al. [70].

For compounds **80** (Figure 8), with isoidide stereochemistry, T_g_ increased in the order I^−^ < Tf_2_N^−^ < PF_6_^−^ < BF_4_^−^, while for compounds **78** (Figure 8), possessing isomannide stereochemistry, T_g_ were generally lower, and they increased in the order I^−^ < BF_4_^−^ < Tf_2_N^−^ < PF_6_^−^. The differences between the two stereoisomers and the effect of the anionic portion were rationalized based on the different nature of hydrogen bonds in the two setereoisomers. While for compounds **80**, only intermolecular hydrogen bonds could be formed, isomannide stereochemistry forms strong intramolecular hydrogen bonds between the 1,2,3-triazolium ring and the C_3_-O-C_6_ ether group, thus leading to lower T_g_ for compounds **78**. In the case of Tf_2_N^−^ as a counter anion, no difference was observed when the stereochemistry was switched, probably due to steric bulk and the weak coordinating effect of this anion that reduced the possibility or the effect of hydrogen bonding. Moreover, the thermal stabilities were influenced by the stereochemistry of the central core: For isoidide stereochemistry, T_d10%_ varied in the order I^−^ < BF_4_^−^ ≈ PF_6_^−^ < Tf_2_N^−^, while for isomannide stereochemistry it varied in the order I^−^ ≈ BF_4_^−^ ≈ PF_6_^−^ < Tf_2_N^−^. The highest stabilities were found for isoidide stereochemistry, and Tf_2_N^−^ salts were the most stable ILs of each series. To conclude, the authors noticed that these ILs were more stable than their neutral triazolium precursors, with a higher increase in thermal stability observed for isoidide derivatives.

In the second work [70], Zullo et al. analyzed new ILs possessing isomannide and isosorbide stereochemistry. (Table 5) Compounds **83a,b** and **84a** (Figure 8), containing iodide as the anion, where all solids at room temperature and did not show a melting temperature in the explored range, since they all degraded before melting. These compounds could not be classified as ILs: By contrast, iodide **84b** (Figure 8) showed a glass transition below 100 °C. The corresponding bis-triflimide salts **85a,b** and **86a,b** (Figure 8) possessed all a glass transitions, in particular, compounds **85a** and **86a** containing an *N*-methyl side chain were characterized by a glass transition about 20 °C higher than that of the corresponding *N*-butyl compounds **85b** and **86b**. These results were considered as a clear indication of the importance of the length of the side chain, while no apparent role could be ascribed to the scaffold stereochemistry. Moreover, the authors stated that the higher glass transitions observed for their Tf_2_N^−^ CILs when compared to those reported in the literature [68] could be ascribed either to their dicationic nature or to the different substituents present on isohexide scaffold or to a combination of these effects.

TGA analysis showed that all the prepared ionic compounds were characterized by several degradation events, with iodide containing compounds **83a,b** and **84a,b** possessing a much wider decomposition temperature range than their bis-triflimide analogs. Moreover, within the iodide salts, the ones containing a methyl chain showed a significant temperature shift for the last degradation event. A high increase in compound stability was observed on moving from I^−^ to Tf_2_N^−^ containing compounds, with the maximum increase being ≈150 °C.

To conclude, a comparison between the two works clearly shows that the dicationic compounds, reported by Zullo et al. [70] are by far more stable than the ones reported by M’Sahel et al. [68]. This result could be ascribed to different aspects; in particular, compounds **83** and **84** possess no free hydroxyl group and a positive charge far from the sugar core. These aspects should contribute to the higher stability of sugar derivatives **83** and **84**. Moreover, while for compounds **78** and **80**, a clear influence of the sugar stereochemistry on the thermal stability is observed, for compounds **83** and **84**, no influence is observed.

### 3.6. d-glucose

d-glucose-derived ILs are by far the most studied ILs from 2011 to date. As a consequence, a survey of the literature reveals many papers dealing with the thermal analysis of these ILs.

**Figure 9:**
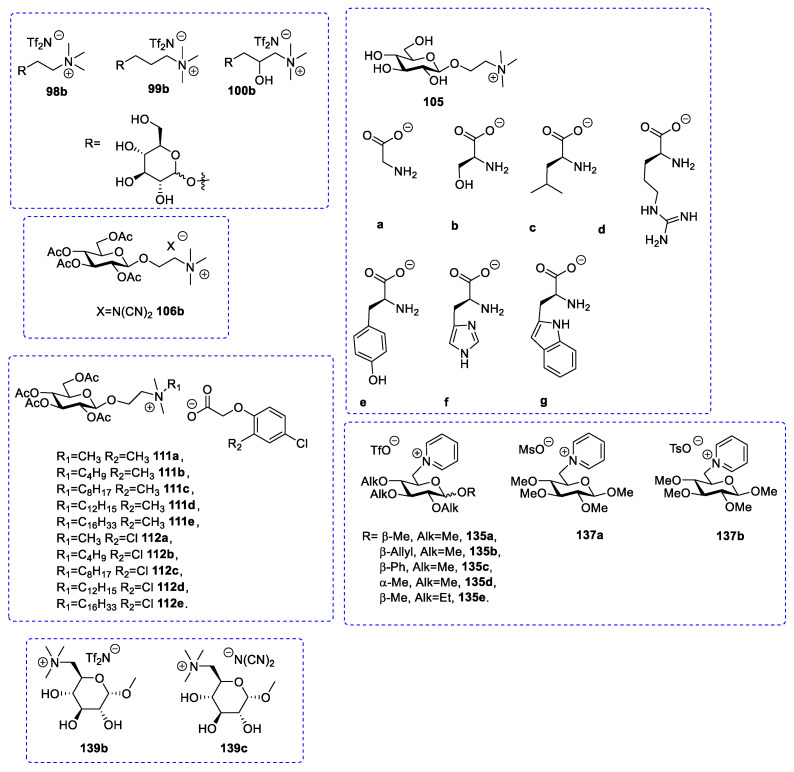
Structures of d-glucose-derived ILs analyzed.

A summary of the data is presented in Table 6, and the studied ILs are depicted in Figure 9.

Pernak et al. reported the thermal analysis of herbicidal-containing ILs in 2016 [74]. In their work, the authors studied compounds **111** and **112** through TGA and DSC analysis.

The results showed that all the compounds could be classified as RTILs, with the exception of **112a** and **112b**. The data showed good thermal stability, with T_d50%_ ranging from 235 °C to 340 °C. In contrast with other reported results [137], the thermal stability of ILs containing an herbicidal anion increased with the length of the alkyl chain, as previously noted by the authors [138].

DSC analysis showed no crystallization upon heating to 150 °C and cooling to −100 °C. Compounds **112** were all amorphous ILs characterized by a high T_g_, ranging from 8.3 °C to 19.5 °C. The thermal properties of the salts were also influenced by the nature of anion; moving from compounds **111** to dichlorinated **112**, an increase of T_g_ was observed. Compound **112b** was the only material that did not show a glass transition in the DSC analysis. No simple relationship T_g_-alkyl chain length could be inferred. For compounds **111**, the T_g_ is higher for compounds containing an alkyl chain –(CH_2_)_n_CH_3_ with *n* = 3.11. With the increase of n, a decrease of T_g_ was observed, with the lowest value recorded for compound **111e**. For compounds **112** no significant variation was observed moving from n = 0 to n = 7, whereas a decrease of T_g_ was recorded when moving to n = 11, and finally, a lower T_g_ was observed for n = 15. For these compounds, the lowest T_g_ was recorded in the case of **112d**.

The research group of Chrobok reported the thermal analysis of compounds **98b**, **99b**, **100b** [75]. All the salts were obtained as a mixture of two anomers, with a 0.7:0.3 α:β ratio for compounds **98b** and **99b** and 1:2 for **100b**. The different anomeric ratio is a variable that must be considered when assessing the results. All the synthesized compounds were RTILs, and DSC analysis showed a T_g_ ranging from −14 °C to −20 °C. When the length of the alkyl spacer was increased, moving from **98b** to **99b**, a rise in the T_g_ was observed; a similar trend was noted, going from **99b** to **100b**. This behavior could be ascribed to the stronger presence of hydrogen bonds, due to the hydroxyl group functionalities on the propyl chain and/or to the differences in the anomeric stereochemistry of the two compounds. The ILs were characterized by good thermal stability, with T_d50%_ ranging from 433 °C to 436 °C and increasing in the order **98b** < **99b** < **100b**. Comparing the thermal stability of these ILs with other synthesized ammonium ILs, the authors found that the presence of hydroxyl groups or a sugar moiety in the ammonium cation had no particular effect on the thermal stability.

In another work, the same group reported the thermal properties of ILs containing an amino acid portion, as well as a glucose core [81]. Compounds **105** were all RTILs and were characterized by a T_g_ ranging from −19 °C to 6 °C. They had a decomposition temperature comprised between 198 °C and 209 °C, with the only exception of the less stable **105d** (T_d_ = 107 °C). The decomposition temperature was not clearly defined, but a closer look at the reported thermograms suggests that it was interpreted as the onset temperature of the first decomposition step or as T_d5%_. As already reported in the literature [139,140], the characteristics of amino acid-containing ILs seemed to depend mostly on the side-chain structure of the amino acid. The elongation of the alkyl side-chain seemed to give higher T_g_ and decomposition temperatures, with the only exception being **99d**, which had the lowest thermal stability. These new ILs showed lower thermal stability than the corresponding imidazolium-based amino acid ILs [139], but they had higher stability than cholinium-containing amino acid ILs [140,141].

The same research group reported the thermal analysis of compounds **98b**, **106b**, and **139b**,**c** [54].

As for compounds **52a** and **52c** (see Section 3.4), the conversion of the parent sugars into ILs and salts enhanced the thermal stability of the products [134]. The authors were primarily interested in the weight of the carbonized residual after thermal decomposition, so the focus was on the final weight obtained after TGA analysis and not on the thermal stability. As mentioned before (see Section 3.4), ILs and salts containing the sugar moiety in the cation were more thermally stable than compounds with the saccharide unit in the anion, mostly due to the decarboxylation of the latter. While compounds **98b** and **139b** could be classified as RTILs and compound **106b** could be classified as IL; compound **139c** had a melting point >100 °C. As expected, the introduction of bis-triflimide anion led to a significant reduction in the melting point [142], with a shift from room temperature solids to RTILs. Moving from compound **106b** to **139c**, an increase in the melting point was observed, which could not be easily rationalized, given the various structural differences between the two products. However, the presence of unprotected hydroxyl groups could account for the formation of a network of hydrogen bonds in **139c** and explain the observed T_m_ increase. Compounds containing Tf_2_N^−^ as the anion were characterized by higher thermal stability, while **139c**, containing ^−^N(CN)_2_ as the anion, was characterized by a lower weight loss [136,143]. Compound **106b**, possessing the same anion, gave a higher weight loss, possibly due to the presence of an ethyl chain spacer that could undergo elimination, resulting in the loss of the ionic character and lower thermal stability. According to the reported thermograms, **106b** shows only one degradation event with onset at ≈300 °C, **98b** displays an early weight loss under 100 °C and other two degradative steps (≈200 °C and ≈400 °C), **139c** shows only one degradative step with an onset comprised between 200 °C and 300 °C, while **139b** presents an initial weight loss from T < 100 °C to ≈300 °C and two other degradative steps at higher temperatures (≈300 °C, ≈400 °C).

Another paper reporting thermal analysis of glucose-derived ILs was published by Reiβ et al. [99]. In their work, the authors analyzed the synthesized ionic compounds using TGA and DSC. From the data reported, **135a** could be defined as an RTIL, **135b**, **135d**, **137a** could be defined as ILs, while the other compounds were only ionic salts. The type and configuration of the group attached to the anomeric position had a large influence on their thermal behavior. The melting point increased in the order β-OMe < β-Oall < β-Oph (**135a** < **b** < **c**), due to the presence of lipophilic groups that generated strong hydrophobic interactions between the cations. Moreover, for compounds **135a** and **135d**, a correlation between the T_m_ and the anomeric configuration could be seen, with β-OMe **135a** being an RTIL, while α-OMe an IL with a melting point of 95–100 °C. The alkyl group attached to the non-anomeric hydroxyl groups also played a role in changing the thermal properties; moving from methyl (**135a**) to ethyl (**135e**), the melting point increased substantially: **135a** was an RTIL, while for **135e** a T_m_ = 118–120 °C was recorded. When focusing on the anion’s role, the melting temperature increased in the order TfO^−^ < MsO^−^ < TsO^−^.

The decomposition temperatures were comprised between 205 °C and 250 °C, but they were not defined. The group present at the anomeric carbon had an effect on the behavior of these molecules: The allyl-containing IL **135b** was the least stable, while **135a** and **135c** had exactly the same decomposition temperature. The configuration at the anomeric carbon seems to, again, have an influence, with the α anomer **135d** being slightly more stable than the β anomer **135a**. At the same time, introducing an ethyl chain on the other hydroxyl groups in place of a methyl group led to a decrease in thermal stability. When the role of the anion was investigated, a trend similar to that seen before was observed: The stability increased in the order TfO^−^ < TsO^−^ < MsO^−^.

### 3.7. Other Monosaccharides: Pentoses

The only examples of thermal analysis on pentoses since 2011 were reported by Reiβ et al. in 2020 [99]. In their work, the authors synthesized several 1-deoxy pentoses, and they analyzed their thermal properties using TGA and DSC. The compounds of interest and the data reported in the paper are collected in Table 7.

All the synthesized compounds could be defined ILs and **177b**-**d**, **178**, **180**, and **182** as RTILs.

The decomposition temperature of these ILs containing a 1-deoxy-pentose fragment ranges from 297 °C to 345 °C, higher than those of the related glucoside-derived compounds **135** and **137** presented in the same paper (see Section 3.6). The different stereochemistry of the sugar core had only a small effect on the thermal stability: The T_d_ (generally defined as “decomposition point”) for compounds **177a**, **178**, **179**, **180** was similar and varied in the order **178** (1-deoxy d-lyxose) < **180** (1-deoxy l-arabinose) < **177a** (1-deoxy d-ribose) = **179** (1-deoxy d-xylose). The functionalization of the hydroxyl groups in the structures seemed to have a larger effect: The thermal stability increased in the order **177d** (-Opropyl) < **177c** (-Oallyl) < **177b** (-Oethyl) < **177a** (-Omethyl), possibly on account of elimination pathways, which are more likely for longer alkyl chains [144]. Surprisingly, the highest ΔT is obtained when going from an allyloxy-substituted system to a propoxy one (ΔT = 66 °C), even if they possess the same number of carbon atoms. This behavior suggests greater stability for ILs bearing a sugar with unsaturated substituents with respect to saturated ones. Compound **182**, featuring free hydroxyl groups, has intermediate thermal stability, with a decomposition temperature comprised between the ones of propyl-substituted **177d** and allyl-substituted **177c**.

The glass transition temperature still depended on the stereochemistry of the sugar core, increasing in the order **178** (1-deoxy d-lyxose) < **180** (1-deoxy l-arabinose) < **179** (1-deoxy d-xylose) < **177a** (1-deoxy d-ribose). For compounds **177** and **182**, which belong to the same stereochemical series, the influence of the alkoxy groups was limited, with the lower T_g_ measured for compound **182** and the highest measured for **177a**.

### 3.8. Disaccharides

The only disaccharide-derived ILs, whose thermal properties were measured from 2011 to date, are xyloside-derived ILs (see Section 3.2) and sucrose-derived IL microgels **187** and **188**.

In particular, Sahiner and Sagbas [117] focused their attention on ILs made up of colloidal microgels of sucrose. The authors found an onset temperature for compounds **188a–c** at about 200 °C. The decompositions ended from 638 to 840 °C, and the thermal stability increased moving from sucrose microgels to modified microgels **188a–c**. (Table 8).

### 3.9. Concluding Remarks

Two main aspects hamper the in-depth analysis and comparison of the thermal properties of sugar-derived ILs reported in the literature, to date: The heterogeneity of the structures prepared, and the heterogeneity of the protocols followed for TGA and DSC. As a result, the identification of possible structural correlations and trends is not trivial.

The first issue stems from the wide chemical space covered by the published sugar-based ILs, which has been explored, though in a somewhat incoherent fashion. The compounds covered in the literature include ILs where the sugar is present as the anion or in the cation and comprise systems characterized by a variety of cations (i.e., ammonium, phosphonium, and imidazolium), by different sugar-cation linkers, by various sugar types, and protecting group patterns.

The second aspect raises even more concerns in drawing any firm conclusion from these properties. Indeed, as mentioned in the first part of this section, outcomes of both TGA and DSC are affected by several operational parameters (i.e., heating rate, mass sample, gas type, and flow) and the lack of a univocally recognized protocol for running these analyses for ILs makes any comparison complicated.

Nevertheless, some information of interest can be extrapolated from the available data. Sugar-based ILs are not intrinsically thermally unstable, as it could be assumed from the general knowledge of sugar reactivity. Depending on the structure and on the protecting group pattern, stability as high as 370.7 °C (IL **85a** [70], based on the onset temperature) has been observed. On the other hand, attention should be paid to the melting point of these structures to possibly classify them as IL with full rights, as the threshold of 100 °C has been exceeded in several cases. Even for this class of ILs the anion metathesis to produce the bis-triflimide salt had a dual positive effect: Increasing the thermal stability and decreasing the melting point. It is also worth stressing that there is no clear evidence that the attaching point of the cation on the sugar frame (usually either the primary hydroxyl group or the anomeric position) has a direct correlation with the thermal stability of the final IL.

Finally, focusing solely on the sugar part of the IL, it is of note that the stereochemistry of the carbohydrate core can play a role in the thermal properties of the whole IL. For example, the α/β anomeric configuration and the sugar stereochemical configuration can affect both the thermal behavior and the thermal stability (i.e., T_m_ and T_d_ of α-OMe-glucoside **135a** versus β-OMe-glucoside **135d**, and T_g_ of glucuronate **52a,b** versus galacturonate **53a,b**) in a homogeneous series of ILs.

## 4. Applications

In the last years, a great deal of research on the use of ionic liquids has been carried out, and the use of these peculiar materials in different areas has already been extensively documented [145]. Concerning sugar-based ILs, it has been shown that these media, endowed with a greener profile in terms of toxicity and biodegradability, can substitute or complement classic ILs in several applications, thus lowering the overall impact of the processes under investigation. Therefore, the following sections will provide an overview of the applications of carbohydrate-derived ionic liquids since 2011. A particular characteristic of ionic liquids, which also applies to sugar-derived ones, is represented by their capability to act at the same time as solvents and catalysts (both chiral and achiral) for organic reactions. Thus, in situations where the role of the IL could not be unambiguously established, the definition given in the original literature report was used to classify that specific IL. An in-depth analysis of all the published literature goes beyond the scope of this review, and therefore, only selected examples will be discussed in each section. For further details concerning the cited studies, please refer to the original papers.

### 4.1. Solvents

The use of ionic liquids as solvents has been widely studied. From the beginning, ILs have attracted a great deal of attention, due to their peculiar properties as “greener” alternative to common organic solvents. As mentioned in the introduction, the *green* concept linked to ILs is mainly related to their negligible vapor pressure and low flammability, while their toxicity and bioaccumulation is still an issue [145].

The dual nature of ionic liquids, with both strongly polar and non-polar groups, represents a great advantage in solute dissolution with respect to classical organic solvents: ILs can establish a great number of different intermolecular interactions, from ionic interactions to directional ones, such as hydrogen bonding, dipole-dipole and dipole-ion [146]. Furthermore, the presence of long non-polar alkyl chains can lead to the formation of three-dimensional dynamic apolar-polar micro-domains, which play a crucial role in the solvation process [146,147,148,149,150]. Changing the structure of the IL will lead to a modification of all these characteristics.

Ionic liquids are amongst the few solvents able to dissolve biomasses: Due to their nature, they can establish a great number of different intermolecular interactions that can facilitate the disruption of strong (hydrogen) bonds present in the pristine biopolymers [146].

For sugar-derived ionic liquids, only one paper concerning the application in biomass dissolution has been published [45].

**Figure 10:**
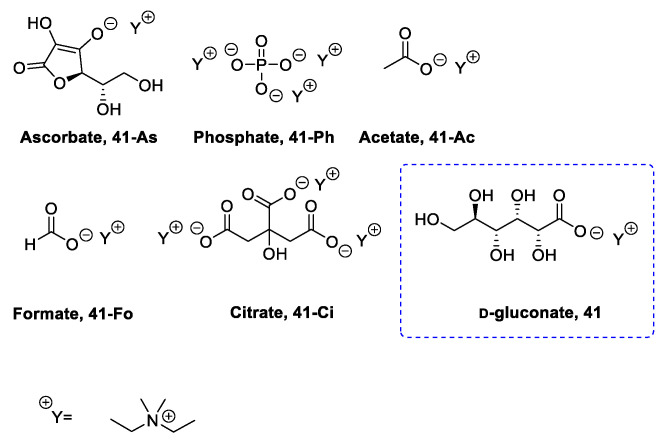
Structures of RTILs studied by Javed et al. in cellulose dissolution, with the d-gluconate-derived ionic liquid **41** highlighted [45].

In this work, Javed et al. synthesized a range of ionic liquids with the same tetra alkyl ammonium cation (Figure 10) and studied their application in cellulose dissolution. Among the obtained ILs, only one possessed a carbohydrate moiety, namely, a gluconate anion. After an initial study on the influence of pH and temperature on the ζ potential of the synthesized ILs, the authors studied their cellulose dissolving capability with real biomass. The initial investigations revealed lower stability and conductivity values at acidic conditions (pH ≈ 1.68), but a significant increase in conductivity and stability at a higher temperature (T = 65 °C) [151].

An oil palm biomass powder containing 25.5% cellulose, 40.0% hemicellulose, 22.8% lignin, and traces of moisture and waxes was used for the dissolution tests. The biomass was dissolved at room temperature in a mixture of RTIL containing 5% *v*/*v* of DMSO; the DMSO was added to facilitate the dissolution process [152,153], but it was also crucial in separating cellulose from biomass [154]. The inter- and intra-molecular hydrogen bonding of cellulose and lignocellulosic biomass were disrupted by the ionic liquid, resulting in the complete dissolution of cellulose, while lignin, wax, and fatty acids were retained in DMSO. The addition of a mixture of water/acetone as the antisolvent leads to complete precipitation of cellulose from the solution. The material was washed and characterized (Table 9). FT-IR spectra of the residue showed that it was pure cellulose, and no lignin was present [155].

This study is very interesting because it highlights the properties of biomass-derived carbohydrate ionic liquids, which perform very well in biomass dissolution. In particular, this approach can be applied in a circular process, starting from biomass to obtain a chemical product that could be used for further biomass valorization, an interesting concept within Green Chemistry.

Another interesting and popular application of ILs as solvents is their use as reaction media [150,156]: Several papers concerning this topic have been published in the last few years [21,22,23,44,55,56,57,61,103,104,106,107,110].

Sugar-derived ILs have been successfully employed as solvent and catalyst/reagent [21,55,56,61,75].

**Scheme 42:**
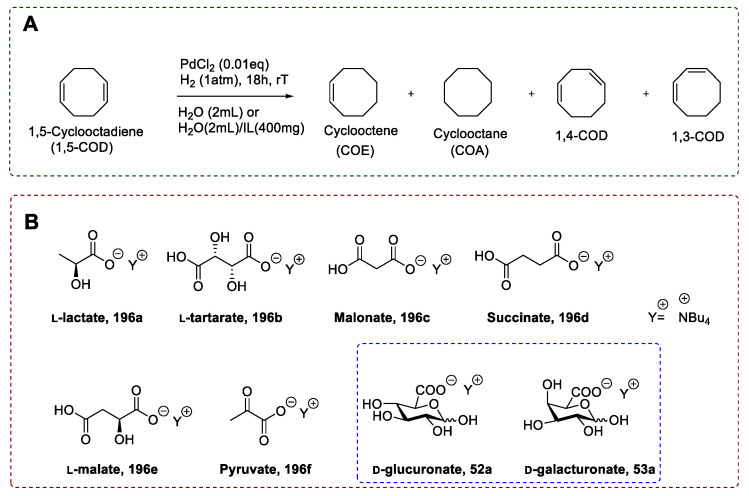
1,5-Cyclooctadiene hydrogenation with the possible products (**A**) and structures of biomass-derived ILs tested (**B**) by Ferlin et al. [55].

In 2013, Ferlin et al. studied the selective hydrogenation of 1,5-cyclooctadiene (1,5-COD) in biomass-derived ammonium ILs [55]. The selective hydrogenation of polyunsaturated compounds is still an issue [157], and many ILs have been employed to achieve better results [158,159,160]. As mentioned before, ILs are non-innocent solvents when toxicity and biodegradability are considered, and for example, many lipophilic ILs are toxic in aqueous media [161,162]. Following the idea that biomass-derived ionic liquids could be more benign, the authors synthesized some new ionic liquids with a tetraalkyl ammonium cation and a biomass-derived anion (Scheme 42).

Ferlin et al. tested the obtained ILs in the reduction of 1,5-COD with PdCl_2_ in the absence of any ligand, employing water as cosolvent, 1 atm of H_2_ for 18h, and running the reaction at room temperature. The addition of a cosolvent was necessary to lower the viscosity of the ILs (or to solubilize the solid ones); as a solvent, water proved to be better than isopropanol, a common cosolvent employed in ILs assisted hydrogenation processes [163], leading to more selective hydrogenation of 1,5-COD to the desired product cyclooctene (COE). The use of an IL as a cosolvent was crucial to obtaining COE already in the first catalytic cycle. Compounds **52a** and **53a**, respectively containing d-glucuronate and d-galacturonate as the anion, showed to be two of the best cosolvents, with **53a** leading to excellent conversions without appreciable undesired isomerization products, even after ten cycles. The selective monohydrogenation in the presence of an IL as cosolvent can be ascribed to partial inhibition of the catalyst, since the second reduction is expected to be more energetically demanding [164]. To conclude this study, the antimicrobial activity towards different bacteria and fungi was tested. Moreover, the biodegradability of these ILs was evaluated. Carbohydrate-containing ILs **52a** and **53a** were shown to be the least toxic but, even if no high antimicrobial toxicity was found, none of the tested ILs passed the Closed Bottle test and were classified as not readily biodegradable (Table 10).

**Scheme 43:**
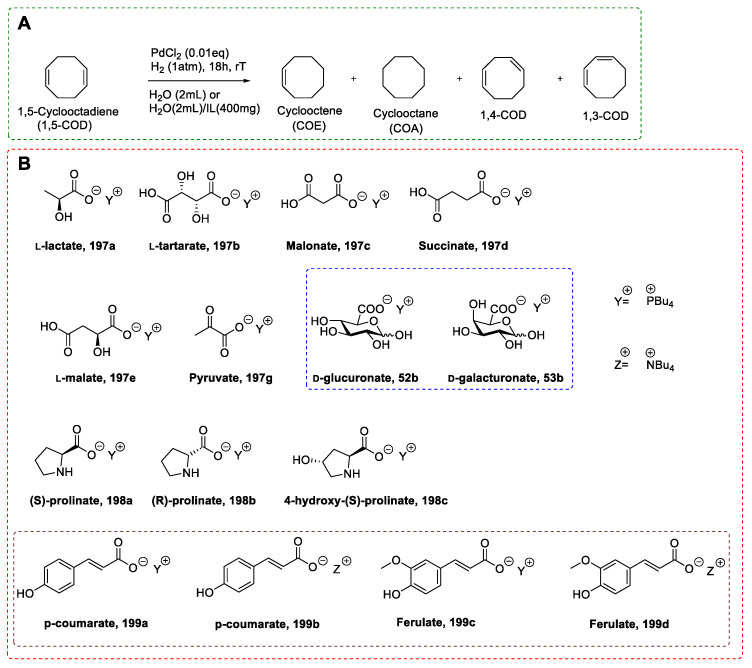
1,5-cyclooctadiene hydrogenation with possible products (**A**) and structures of biomass-derived ILs synthesized in this study (**B**) [56]. Sugar-derived ILs are highlighted in blue, while compounds in the dark red box were not employed in this study, due to their low thermal stability.

As a continuation of the previous study, in 2016, Hayouni et al. synthesized a range of phosphonium ionic liquids, as well as some other ILs from biomass-derived carboxylic acids. The authors employed them not only in the Pd-catalyzed hydrogenation of 1,5-COD, but also in the hydrogenation of vegetable oils and in the enantioselective hydrogenation of unsaturated ketones (Scheme 43). All the ILs employed gave comparable results in the reference reaction and using water as cosolvent: The product consisted of cyclooctane and/or a mixture of alkanes and alkenes without any traces of isomerization products. The presence of a phosphonium, instead of an ammonium cation, led to the loss of the desired selectivity (Scheme 43).

**Scheme 44:**
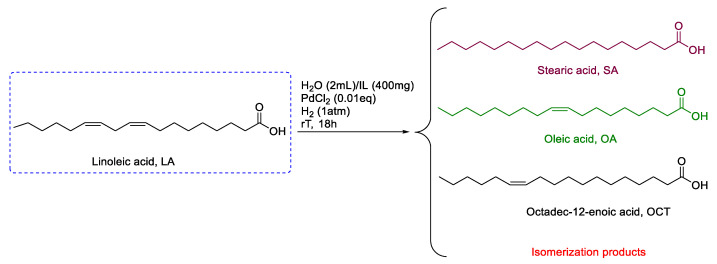
Linoleic acid reduction, as studied by Ferlin et al. and possible products [56].

The authors moved then to the study of the hydrogenation of linoleic acid (LA), a polyunsaturated fatty acid present in large amounts in vegetables oils. **(Scheme 44**) Its hydrogenation can lead to the complete saturated product, stearic acid (SA), or can produce monounsaturated *cis*-octadec-12-enoic acid (OCT), oleic acid (OA), and their isomerization products.

With the aim to obtain only a *cis* monounsaturated product without isomerization of the double bonds, the authors tested the synthesized ionic liquids under the same conditions employed in the model reaction. The analysis of the crude mixture showed that all the ILs led to selective hydrogenation, gave monounsaturated products without any isomerization of the double bonds, and generally showed high conversion of the starting material (Scheme 44). In contrast to the results obtained in the model reaction with 1,5-COD, no anion or viscosity effect was observed.

Another area of interest for ILs as solvents is represented by their use as extraction media, which has been well discussed and described by Aguilera-Herrador et al. [165]. This application constitutes by far the most common use for sugar-derived ionic liquids since 2011 [22,23,44,57,103,104,106,107,110].

**Figure 11:**
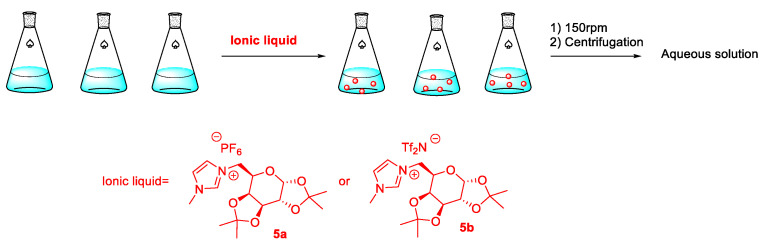
Schematic representation of the extraction protocol employed by Jayachandra et al. [22,23].

For instance, d-galactose-derived hydrophobic ionic liquids **5a,b** have been employed for the extraction of either Pb^2+^ [23] or Cd^2+^ [22] from aqueous solutions, as reported by Jayachandra et al. (Figure 11).

In the former work, the authors synthesized a water-insoluble PF_6_^—^containing ionic liquid (**5a**), which was solid at room temperature [23]. The state of matter and the hydrophilicity were considered two important features for the effective extraction of metal cations from water. The authors studied the effect of different parameters on the extraction efficiency, such as pH, temperature, salt concentration, and contact time. The experiments were performed simply stirring the dispersion of the solids in an aqueous solution containing the metal cation (Figure 11). The results obtained showed that the best conditions were acidic (pH = 5), 30 min of contact time for assuring saturation of the active sites of the IL, and low amounts of salts. A desorption study, carried out with 0.01M HCl as a desorbing agent, showed that up to 99% of the extracted Pb^2+^ could be eliminated from the ionic compound and that the absorption-desorption cycle could be repeated at least four times. Other work was carried out to fully characterize the process, such as the determination of absorption isotherms, the analysis of the kinetic and the thermodynamic of absorption, the study of the impact of other cations, and in general, investigations on the mechanism of absorption.

To evaluate the maximum metal ion loading capacity, the IL was tested with different initial concentrations (10–50 mg L^−1^) of Pb^2+^ ions at equilibrium. The metal ion loading capacity increased with the initial metal ion concentration, reaching a maximum of 374.9 mg g^−1^ and the salt **5a** was selective towards the extraction of Pb^2+^, possibly on account of the higher electronegativity and smaller ionic radius compared to other metal cations screened, such as Ni^2+^, Cd^2+^, Cu^2+^ (Figure 11).

With the aim of expanding on their previous findings, the authors synthesized bis-triflimide **5b** and fully characterized its ability to extract Cd^2+^ ions from real-life wastewaters (Figure 11) [22]. In this case, the synthesized compound could be classified as an ionic liquid. Unfortunately, no selectivity test was carried out for **5b** in the presence of Pb^2+^ cations. Hence, no conclusions can be drawn about the cation extraction selectivity.

Still related to extraction methodologies, carbohydrate-derived ionic liquids have been employed in dispersive liquid-liquid microextraction (DLLME) [103,104,106] or as coatings for solid-phase microextraction (SPME) [107,110]. These ILs applications were also exhaustively described by Aguilera-Herrador et al. [165].

**Figure 12:**
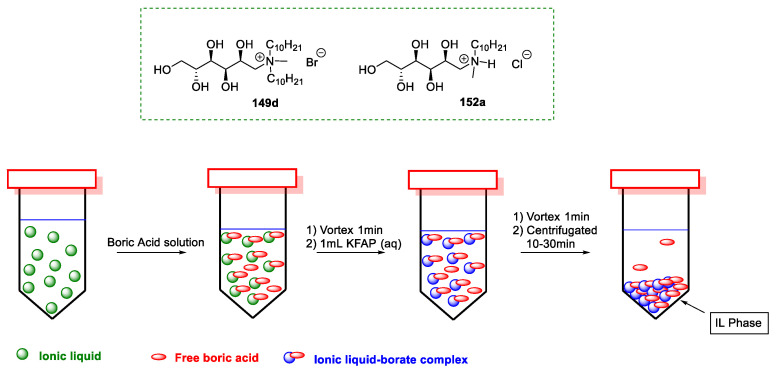
A schematic representation of the dispersive liquid-liquid microextraction (DLLME) procedure employed by Joshi et al. [103].

In 2012, Joshi et al. [103] studied the application of a glucaminium-based ionic liquids for the removal of boron derivatives from water (Figure 12). The most abundant boron species in water and soil are boric acid or borate salts, depending on pH: Boric acid is predominant at pH < 7, while borate anions are found at pH > 10 [166]. Although boron is an essential micronutrient for plant growth, high levels of boron in irrigation water can lead to plant death [167]. Moreover, high levels of boron in drinking water can have teratogenic effects in humans [168]. Due to these effects, the World Health Organization (WHO) has set a threshold of 0.5 ppm in drinking water and 0.3–0.5 ppm in irrigation water [169]. So far, very good results have been obtained in boron removal from water employing exchange resins functionalized with carbohydrates moieties such as *N*-methyl-glucamine [170,171], making the use of ILs derived from this starting material promising. To fulfill this goal, the design of the ionic liquid is crucial: The final product should possess diol functional groups (usually of *cis* configuration), able to bind the boron species, and its hydrophilicity should lead to a biphasic system with water.

*N*-Methyl-d-glucamine-containing ILs fulfills these requirements, since the alkylation of the amino group can be used to mitigate the polarity of the carbohydrate moiety and to shield the amino group (so that it cannot interfere with the boron complexation). Finally, a simple anion metathesis can afford a hydrophobic IL starting from a hydrophilic analog.

The authors started their work studying the complexation of boron species with the hydrophilic chlorine- and bromine-containing ILs **152a** and **149d**. The complexation mechanism and the effect of pH were studied by ^11^B NMR analysis (Figure 12).

The extraction performance of compounds **152a** and **149d** and their capability to easily form a hydrophobic phase and pre-concentrate the boron species were studied using in situ DLLME [171]. In situ DLLME was performed, dissolving a water-soluble ionic liquid in an aqueous solution of the analyte and generating a hydrophobic phase through a simple anion metathesis reaction with tris(pentafluoroethyl)trifluorophosphate (KFAP). As a consequence, the IL phase-separated from water, and the boron-containing species concentrated in the small volume of this phase (Figure 12). HPLC analysis of the residual aqueous solution showed the high efficiency of this protocol, while the importance of free *cis* hydroxyl groups was proven through ^11^B NMR when the use of the peracetylated analog of **152a** failed to show any boron complexation.

As a continuation of this study, Joshi et al. explored a DLLME employing LiTf_2_N in the anion metathesis step [106]. The authors expanded their scope by studying a different salt, as KFAP was not commercially available. Interestingly, they noticed that the use of LiTf_2_N in conjunction with 0.1M NaCl_(aq)_ solution, which was added to have a good phase-separation, led to better results. They proved that there was no influence of the nature of the anion on the extraction performance, but the effectiveness depended on the better phase-separation observed in this case. To conclude, these studies describe a collection of positive results regarding the use of sugar-derived ILs in the extraction of boron compounds. These were gathered through a model seawater assay and via recycle of the extraction media. Only compound **149d** was used in DLLME with Tf_2_N^−^ anion.

In the area of SPME, the attention was mainly directed towards the development of polymeric ionic liquids (PILs) that could be used as coatings [172].

**Figure 13:**
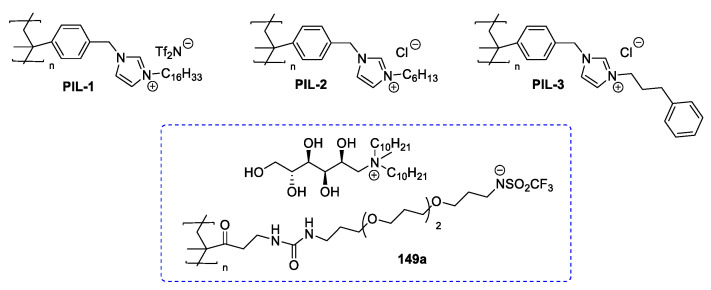
Polymeric ionic liquids (PILs) employed as coatings for SPME fibers by Ho et al. [110]. The sugar-containing PIL **149a** is highlighted in blue.

In 2012, Ho et al. studied the use of different PILs as coatings for SPME fibers [110]. In their work, the authors studied four different PILs, one of which was sugar-derived and contained an *N*-methyl-d-glucamine (**149a**) (Figure 13).

The structure of the polymeric coating was chosen to enhance the intermolecular interactions and the selectivity towards the analytes of interest, i.e., genotoxic alkyl and aryl aldehydes. In this study, a detailed account of the optimization and validation of SPME extractions with these new coatings, which reached high selectivity and low LODs, is presented. Furthermore, recovery studies showed that this protocol could be applied to the quantitative determination of the above-mentioned genotoxic impurities at ppb levels. The same results were obtained in the extraction of other genotoxic impurities, such as alkyl halides.

**Figure 14:**
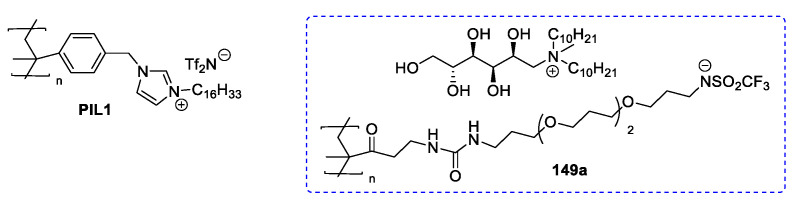
Polymeric ionic liquids employed as coatings for SPME fibers by Gionfriddo et al. [107]. The sugar-containing PIL **149a** is highlighted in blue.

Following this idea, in 2018, Gionfriddo et al. [107] employed two of the previous PILs for the extraction of polar analytes (Figure 14). Given their enhanced features with respect to commercially available SPME coatings [173,174], these materials were considered particularly useful in food analysis where there is a need for efficient extraction of polar and non-polar analytes. The authors compared these new coatings with commercial SPME fibers in the determination of fruit metabolites and organo-phosphorous pesticides. An initial study on the coating lifetimes showed that **PIL-1** was quite stable and durable, while **149a**, containing a sugar moiety, was more subject to deterioration.

### 4.2. Catalysts and Ligands

Another interesting application of ionic liquids is their use as catalysts. Due to their ability to engage in a variety of intermolecular interactions, as well as the possibility to easily alter their structure, it is possible to tune the ILs properties and to make them fit the requirements of a particular reaction.

Some papers concerning the use of carbohydrate-derived ionic liquids as catalysts have been published [72,81,109,175].

**Scheme 45:**
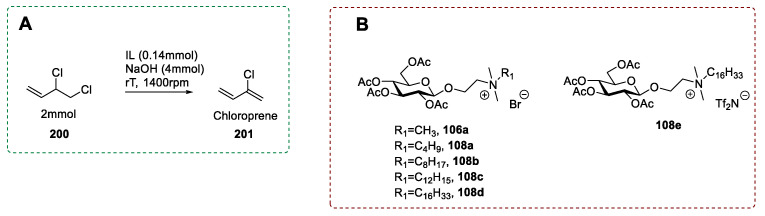
Dehydrohalogenation reaction of 3,4-dichlorobut-1-ene **200** to yield chloroprene **201**, as reported by Erfurt et al. (**A**) and the ILs employed in this study (**B**) [72]. The optimal reaction conditions were found employing **108d** as PTC.

Due to their peculiar nature, characterized by the presence of long non-polar chains and an ionic group, ILs can be successfully employed as phase transfer catalysts (PTC) [176,177,178,179,180,181]. In a 2020 representative work, Erfurt et al. studied the use of d-glucose-derived ionic liquids **106a** and **108** as benign catalysts for the industrial synthesis of 2-chloro-1,3-butadiene (chloroprene, **201**) (Scheme 45) [72], a compound employed in the synthesis of cross-linked rubbers, with applications spanning from adhesives to integral automotive components [182]. The study had the aim of valorizing the use of carbohydrate-derived ILs, with a particular emphasis on using less toxic and more biodegradable catalysts.

The influence of different parameters on the results of the model reaction, such as the structure of the PTC (i.e., the length of the alkyl chain), the stirring rate, as well as the concentration and amount of base, was studied. It was found that the use of an organic solvent was not required, representing a great advantage for industrial applications, meaning lower costs, and easier separation process, and smaller reactors. Moreover, no water was needed as the reaction gave the same result—both with an aqueous sodium hydroxide solution and with the same base in solid form. In both these cases, the ionic catalyst could be used as the reaction medium. The best results were obtained employing compound **108c**, which could be recycled five times without any loss of activity and led to the desired product in almost quantitative yield after just one hour (Scheme 45). Compound **108d** was not used in the optimization tests, due to the foaming of the crude mixture that made impossible to carry out the reaction.

The cytotoxicity and biodegradability tests on these ILs showed that **106a** had low cytotoxicity and was degraded to a high extent. Moreover, the cytotoxicity and biodegradability seemed to be related to both the nature of the anion and to the length of the alkyl chain, with **106a** having the lowest cytotoxicity and the higher biodegradability, while **108d-e** had the poorest behavior (Table 11).

Another appealing application of sugar-derived ILs, somewhat related to their use as catalysts, is their potential as ligands for metals. This area of research is well-established and relies on ionic liquids that can be used as ligand precursors or added to reaction mixtures to generate in situ *N*-heterocyclic carbene (NHC) ligands [150,184].

Apart from some very specific applications [48,82], the main interest has traditionally been in the use of these ILs as Pd ligands for Suzuki reaction, with Zou et al. being the main research group active in this field [73,84,85,86].

The use of carbohydrate-derived ILs could allow for a greener protocol of Pd-catalyzed cross-couplings, using more benign ligands and the improvement in both ligand and catalyst recycling.

**Figure 15:**
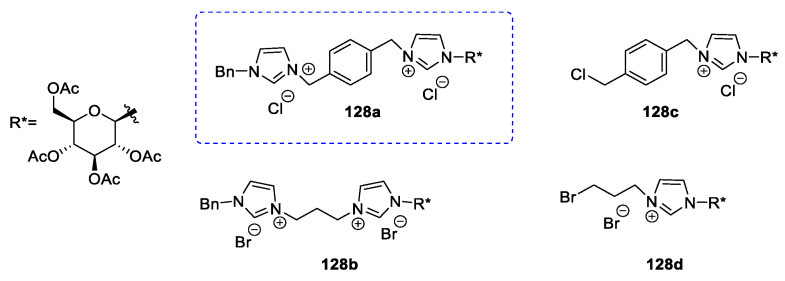
Sugar-derived ILs employed as ligands in Suzuki coupling by Zou et al. [84]. The best ligand (**128a**) is highlighted in blue.

For example, in 2018, Zhou et al. [84] developed an efficient Pd-catalyzed aryl-aryl Suzuki cross-coupling protocol for synthesizing organic functional materials (OFMs) using a mixture of water and ethanol as solvent. The authors started from an optimization of the conditions, studying the coupling between 2-bromofluorene **202** and phenyl boronic acid as model reactions. The results showed that the reaction could be performed under air atmosphere with a reduced amount of Pd catalyst (0.01mol%). The ligand employed had a strong influence on the yield, with bis-imidazolium **128a**, characterized by a more rigid linker between the cationic moieties, being the best of the series (Figure 15).

**Scheme 46:**
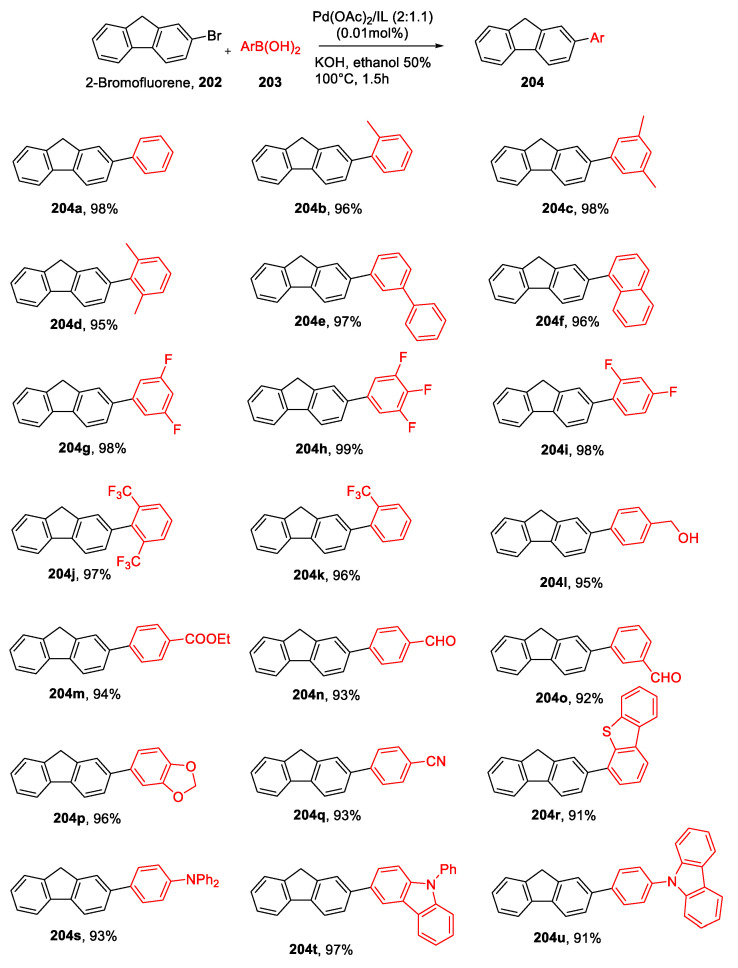
Examples of 2-arylfluorene derivatives **204** synthesized by Zhou et al. [84].

The optimized reaction conditions (Pd(OAc)_2_/**128a** (2:1.1) (Pd 0.01 mol%), 0.5mmol 2-bromofluorene **202**, 0.75 mmol arylboronic acid **203**, 1.0 mmol of KOH, 3.0 mL ethanol/water (1:1), 100 °C, 1.5 h, air) were then employed successfully for synthesizing a wide range of biaryl compounds (21 examples of 2-arylfluorene derivatives **204** and 12 examples of 2,7-diaryl-9,9′-dialkylfluorene derivatives) (Scheme 46). Finally, a recycling test showed good results even after five cycles.

The research group of Zhou also studied the use of glucose-derived ILs as starting materials for the in situ synthesis of ultra-small, water-soluble, and stable Pd nanoparticles. In this example, the metallic species were stabilized by the coordination of *N*-heterocyclic carbenes resulting from the sugar-containing IL [87].

In another example of notable research in the area encompassing carbenes and ILs, Carcedo et al. reported, in 2011 [67], that the synthesis of isomannide-derived *N*-heterocyclic carbenes and the characterization on metallamacrocycles containing these bridging ligands.

### 4.3. Asymmetric Synthesis and Enantiodiscrimination

Ionic liquids obtained from sugars preserve the chiral information of the parent compound, so they can be defined as chiral ionic liquids (CILs), and they can be employed in asymmetric synthesis and enantiorecognition processes.

In Section 4.1, mention was made of some work towards the enantioselective Michael addition of malonates to activated alkenes [21], where sugar-derived ionic liquids were employed both as cosolvents and catalysts.

The main applications of sugar-derived CILs, from 2011 to date, are in enantiorecognition processes [24,39,70] (e.g., the study of Mosher acid’s salt enantiodiscrimination) or in asymmetric synthesis [21,24,40,75,185].

As a continuation of a previous work [21], in 2016, Jayachandra et al. employed a selection of xylose-derived CILs **35** as organocatalysts for the enantioselective Michael addition reaction [40] between chalcone **205** and diethylmalonate **206** in chloroform, using potassium carbonate as a base at room temperature (Table 12).

Good yields of the addition product **207** were obtained, albeit with a very low enantiomeric excess (*ee*); gratifyingly, lowering the temperature to 0 °C led to a higher *ee* (33%). However, a further decrease of the temperature to −40 °C led to a worsening of the results, and even using the most active malonitrile, only 5% *ee* and 85% yield was obtained (Table 12).

In 2017, Yuan et al. reported the use of chiral glucose-containing pyridinium ILs in the asymmetric synthesis of Troger’s bases (TBs) [83], which are characterized by interesting structural features. They possess two different chiral elements: Two stereogenic bridgehead tertiary nitrogen atoms and a C_2_ axis. As a result, they can exist only as (−)-(R,R) or (+)-(S,S) enantiomers [186]. The two bridgehead nitrogen atoms are H-bond acceptors and act as the main catalytically active sites, while the aromatic planes are characterized by a dihedral angle [187] of 80–114°. This rather rigid structure, resembling a chiral cavity roughly 1 nm long [188,189], makes these compounds potential as ligands in asymmetric applications. Moreover, various parts of their skeleton can be structurally altered, which gives the potential for refining their properties. Finally, TBs possess a V-shaped structure characterized by a highly hydrophobic pocket that interacts with different substrates. Despite these interesting features, the availability of TB is very restricted [190,191,192], mainly due to two issues connected to its synthesis: The side products obtained during its preparation [193,194,195], and its tendency to slowly racemize in dilute acidic media, polar solvents or if exposed to high temperatures [186]. In their work, the authors decided to employ the glucose-derived CIL **125a** as a catalyst in the asymmetric synthesis of TBs, encouraged by a related, previously reported racemic preparation performed in IL [196].

The CIL of interest was simple to synthesize, and the developed protocol was inexpensive and scalable up to a 50 g scale. After an initial optimization of the reaction conditions, the authors prepared a series of chiral Troger’s bases **210** with good yields (up to 83%) and excellent *ee* (up to >99%) (Table 13). Following up on these successful applications, an in-depth study of the reaction mechanism, highlighting the main factors that influenced the enantioselectivity, was also reported [83].

### 4.4. Surfactants

Aiming to improve conventional products in terms of biodegradability and toxicity, sugar-based surfactants have been used as detergents, dishwashing agents, and personal care products [197]. Surfactants based on glycolipids represent the most studied group and are the most promising class for commercial production, for industrial use, as well as for biomedical applications [198]. Among the great number of different anionic, cationic, and non-ionic sugar-based surfactants described in the literature [199,200,201], all characterized by mild production conditions, lower toxicity, high biodegradability, and environmental compatibility, glucose-based amphiphiles are prominent [202,203,204].

Since 2011, a great number of papers concerning the synthesis of sugar-based surfactants have been published. It is interesting to note that a large proportion of these materials can qualify as IL. This is not surprising given that ionic surfactants and ionic liquids share similar structural features, and a melting point below 100 °C makes an ionic surfactant a *de facto* IL.

Different starting materials have been employed for synthesizing carbohydrate-based surfactants (d-galactose [28], d-gluconic acid [47,49,50,205], lactobionic acid [47,50], d-glucose [77,78,79], d-mannose [33], d-galactose [33], d-lactose [77], d-glucamine [102,111]), in all cases leading to final products containing a cationic nitrogen on the sugar moiety.

**Figure 16:**
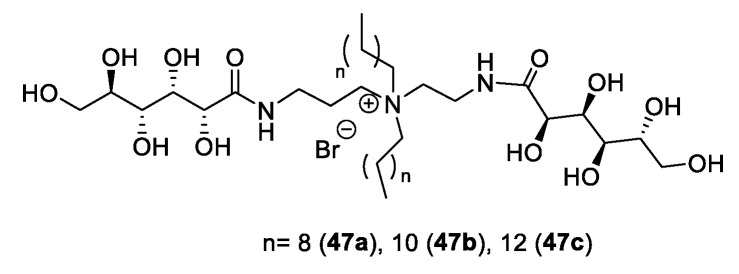
The structure of star-shaped ILs surfactants, as reported by Zhi et al. [49].

For example, in 2014, Zhi et al. reported the synthesis of three novel, star-shaped cationic surfactants **47**, starting from gluconic acid [49]. In their work, the authors synthesized amphiphiles **47a–c** containing two units of gluconamide, starting from glucono-δ-lactone and differing from each other for the length of alkyl chains. (Figure 16).

The products were fully characterized to determine their properties as surfactants in terms of surface tension (static and dynamic), critical micellar concentration, shape, and morphology of micellar aggregates (by electrical conductivity, transmission electric microscope (TEM), and dynamic light scattering (DLS)). These kinds of measurements are very common in the field of surfactants characterization, and they can also be found in all the other cited papers concerning this topic.

Glycolipid cationic surfactants can be used as drug-delivery systems, due to their ability to form micelles and vesicles; however, from 2011 to date, only two papers dealing with preliminary studies on this subject were published [33,79].

### 4.5. Chromatography and Electrophoresis

The coexistence of ionic groups together with organic, apolar moieties, as well as the possibility to introduce a wide range of functional groups, make ILs a very interesting class of compounds. This is particularly relevant for their use in chromatography and electrophoresis, in which the ability to establish a range of varied intermolecular interactions is required.

In the field of chromatographic separations, ILs are mainly used as mobile phases additives in high performance liquid chromatography (HPLC) [206,207,208,209,210], stationary phases for HPLC [209,210,211,212], and gas chromatography (GC) [209,213], as well as buffer electrolytes or additives in capillary electrophoresis (CE) [209,210]. Their particular characteristics make them able to retain a great number of different analytes through different mechanisms, such as ionic, electrostatic, dipole-dipole, and π-π interactions [211,212,214].

Since 2011, a few papers on these topics have been published, concerning the use of sugar-derived ILs as stationary phases in hydrophilic interaction chromatography (HILIC) [105,108], as additives in reversed-phase high performance liquid chromatography (HPLC) [112], and in protein absorption [117].

Hydrophilic interaction chromatography (HILIC) is a chromatographic technique particularly suited to the separation of polar and hydrophilic analytes. HILIC can effectively retain polar compounds by using polar stationary phases, and at the same time, it can resolve the insolubility of sample and incompatibility with MS encountered in normal-phase liquid chromatography, by adopting water and a water-soluble solvent composition as the mobile phase similar to that used in reverse-phase.

**Scheme 47:**
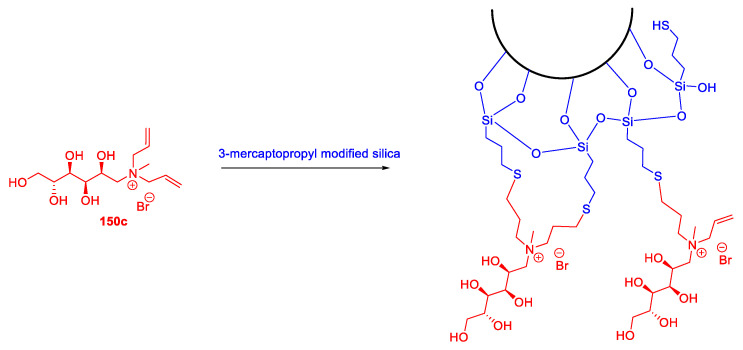
A schematic representation of stationary phase’s preparation starting from **150c**, as reported by Qiao et al. [105].

In 2014, Qiao et al. reported the preparation of a new stationary phase based on a glucaminium-derived ionic liquid (**150c**) and its application in HILIC [105]. In their work, the authors started from *N*-methyl-d-glucamine to obtain an IL containing two allyl chains, which were exploited for the successive immobilization step on 3-mercaptopropyl modified silica gel (Scheme 47). The immobilized stationary phase was characterized in terms of the influence of different parameters (i.e., salt and water concentration, as well as the acidity of the mobile phase) on the retention behavior. This new stationary phase was then employed in the separation of mixtures of flavonoids and nucleotides, obtaining good results in terms of separation efficiency in HILIC and mixed-mode HILIC/anion exchange chromatography.

Another interesting application of ILs in separation techniques is represented by their use in capillary electrophoresis.

Capillary electrophoresis (CE) can be considered a type of liquid phase-separation characterized by a high voltage direct current electric field as driving force and a capillary as separation channel. CE possesses the features of both liquid chromatography and electrophoresis, even if it is characterized by lower cost, higher simplicity, and efficiency [215,216,217,218,219].

The chiral version of CE can be realized following three different strategies: (a) Binding a chiral selector on the capillary column; (b) adding a chiral compound to the running buffer; (c) derivatizing the racemic analytes with a chiral enantiopure compound [220,221,222,223]. The preferred methodology is by far the second one, as the addition of a chiral compound to the running buffer is operationally simple and leads to good results.

From 2011 to date, Xu et al. reported two examples of carbohydrate-containing ILs as a chiral additive in CE [58,60].

**Figure 17:**
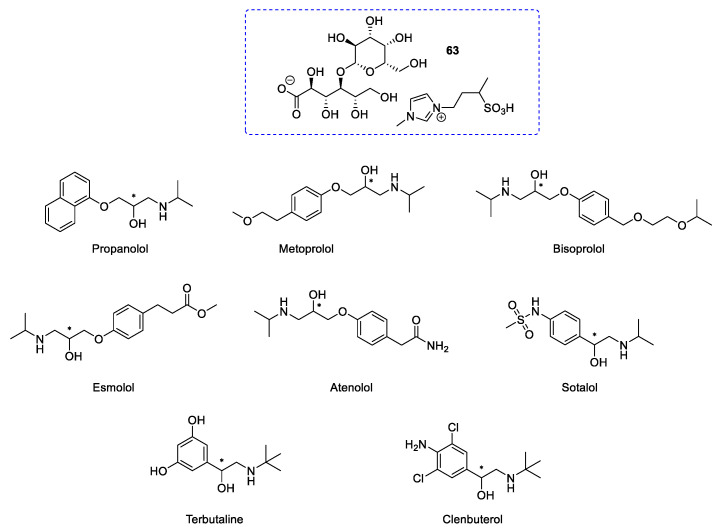
Lactobionic-derived IL **63** employed by Xu et al. in chiral capillary electrophoresis (CE) [58], and the analytes tested in their study.

In the first paper, published in 2019 [58], the authors presented the synthesis and the use of a lactobionic-derived ionic liquid **63** in CE, supporting their studies with a successful enantiomeric separation of a panel of drugs (Figure 17). Different aspects of the separation process were also discussed with the aid of molecular modeling.

### 4.6. Miscellaneous Applications

Ionic liquids are characterized by a great versatility that makes them useful in a wide range of areas.

Sugar-derived ILs make no exception, and many papers concerning very diverse applications have been published from 2011 to date. To conclude this section, a brief overview of those studies which could not be included in any of the previous categories is presented.

Sugar-based ILs have been used as dopants in the electrodeposition of poly(3,4-ethylenedioxythiophene) (PEDOT) for preparing highly corrugated, structured polymer films [76], as versatile precursors for preparing nitrogen-doped carbonaceous materials [54], as herbicides [74], and in the synthesis of new polymers with antibacterial properties [133,134].

Finally, a great number of papers deal with the synthesis and characterization of sugar-derived ionic liquids, like the study of their reduced toxicity, due to the introduction of a sugar unity [17], the determination of their mutagenic activity [32], the measurement of their cell viability and cytotoxicity [99], the investigation of their physicochemical properties [34,38,43,65,66,68,82,115], and their interactions with common solvents [46].

Although these aspects are of the highest interest in defining the potential and scope of sugar-based ILs, a detailed description goes beyond the aim of this work, and further details can be found in the literature.

## 5. Future Perspective

Sugar-based and sugar-derived ILs belong to the still limited subset of bio-based ILs. However, there is no doubt that this intriguing type of ILs will experience significant growth in the next future in parallel with, and as a consequence of, biomasses exploitation for the preparation of renewable chemicals. A recent paper by the group of Ananikov [224] is a clear example of this trend.

From the synthetic point of view, sugar-based ILs can be accessed nowadays by two main strategies: (a) Through a one-step neutralization reaction (for instance, gluconic acid and glucuronic acid as an acidic partner, glucosamine and glucamine as basic partners) or (b) through multistep, more synthetically demanding approaches. The former is the option of choice if large volumes are to be prepared, while the latter allows for the design of (chiral) bio-based catalysts, which have been proven efficient, for instance, in the industrially relevant synthesis of 2-choro-1,3-butadiene [72].

Two examples are particularly interesting, namely, the preparation of sugar-based ILs directly from the parent polysaccharide [38], and the use of sugar-based ILs for the dissolution of cellulose [45]. Indeed, these applications represent two steps of a potential closed-loop process, where biopolymers are the source for their own dissolution media, and will deserve further investigations.

It is also worth mentioning that the work by D’Anna et al. [17] showed for the first time the beneficial role of a sugar appendage on traditional imidazolium ILs in terms of mitigating their toxicity. This result is relevant and could well give new inputs for the development of sugar-classic ILs.

Furthermore, remarkable results have been obtained by combining sugar frames with the cholinium cation and/or with amino acids for the preparation of new bio-based ILs. These structures could represent the optimal approach for addressing toxicity and biodegradability issues, and it is clear that further work will be carried out in this exciting area in the near future.

This research area would certainly benefit from additional investigations targeting more systematic structural variations. These studies should focus both on the assessment of the ILs’ physico-chemical properties and on their potential applications. For what concerns the former aspect, univocal standard protocols should be defined, as outlined in the thermal behavior analysis section. In so doing, it will be possible to discover and assess trends and differences within the same sugar type or among different scaffolds. These studies will help to fill fundamental knowledge gaps around sugar-based ILs with a particular emphasis on the subtle effect of sugar stereochemistry, an aspect that has been seldom highlighted so far.

Although a great deal of work has been carried out in the last few years, which highlighted the suitability of sugar-based ILs in several applications, these ILs are without a doubt the bio-based ILs with the highest unexpressed potential.

## Figures and Tables

**Table 1:**
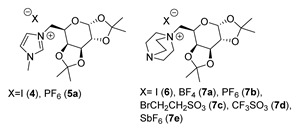
Structures and thermal properties of d-galactose-derived ILs.

	Physical State	T_m_(°C)	T_d_(°C)	Ref.
**4**	Colorless solid	196–198 ^1^ (202.9) ^2^	261.0 ^2,3,4^	[23]
**5a**	Colorless solid	142–145 ^1^ (136.5) ^2^	266.7 ^2,3,4^	[23]
**6**	White solid	not reported	189–192 ^1^	[23]
**7a**	Off-white solid	not reported	222–224 ^1^	[24]
**7b**	White solid	not reported	249–252 ^1^	[24]
**7c**	Light green solid	not reported	198–200 ^1^	[24]
**7d**	Off-white solid	not reported	178–182 ^1^	[24]
**7e**	White solid	not reported	202–206 ^1^	[24]

^1^ Measured on a digital melting point apparatus; ^2^ Measured as the onset temperature by DSC analysis on ≈2 mg of sample. ^3^ No information on the instrumental apparatus and the experimental procedure was reported; ^4^ T_max_ of the corresponding endothermic peak recorded by DSC analysis.

**Table 2:**
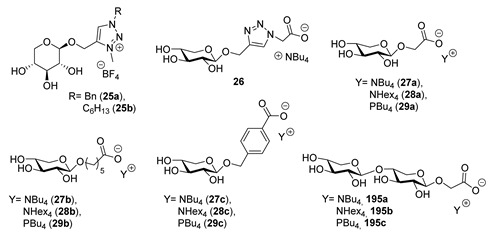
Thermal properties and structures of d-xylose and xyloside-derived ILs.

	Physical State	T_m_(°C)	T_g_(°C)	T_d_(°C)	Ref.
**25a**	White wax	---	4 ^1,2,3^	150 ^4^	[34]
**25b**	White wax	---	2.7 ^1,2,3^	120 ^4^	[34]
**26**	Oil	---	−26 ^5^	≈140–260 ^6,7^	[38]
**27a**	Solid	59 ^8^ (58) ^5^	---	≈140–260 ^6,7^	[38]
**27b**	Oil	---	−35 ^5^	≈140–260 ^6,7^	[38]
**27c**	Solid	98 ^8^ (77) ^5^	−32 ^5^	≈140–260 ^6,7^	[38]
**28a**	Solid	74 ^8,9^	−4 ^5^	≈140–260 ^6,7^	[38]
**28b**	Oil	---	−30 ^5^	≈140–260 ^6,7^	[38]
**28c**	Solid	112 ^5,9^	−4 ^5^	≈140–260 ^6,7^	[38]
**29a**	Oil	---	---	≈140–260 ^6,7^	[38]
**29b**	Oil	---	0 ^5^	≈140–260 ^6,7^	[38]
**29c**	Oil	---	−19 ^5^	≈140–260 ^6,7^	[38]
**195a**	Solid	^9^	−32 ^5^	≈140–260 ^6,7^	[38]
**195b**	Solid	93 ^5,9^	16 ^5^	≈140–260 ^6,7^	[38]
**195c**	Oil	---	2 ^5^	≈140–260 ^6,7^	[38]

^1^ Onset temperature; ^2^ Determined by DSC; ^3^ DSC conditions: 10–15 mg of compound, aluminum crucibles, 10 °C/min, dry Ar (200 mL/min) ^4^ TGA conditions: 30–300 °C, 10 °C/min, alumina crucibles, dry Ar (50 mL/min); ^5^ DSC conditions: ≈2 mg of compound, 5 °C/min, aluminum crucibles, dry N_2_ (50 mL/min); ^6^ TGA conditions: 10–20 mg of sample, aluminum capsule for liquid or viscous samples, dry N_2_ (60 mL/min), heating cycle: 5 min at 50 °C then 10 °C/min to 400 °C and finally 15 min at 400 °C; ^7^ The authors only reported this interval of decomposition temperature; no T_d_ is reported for each compound; ^8^ Determined on a Stuart™ melting point apparatus; ^9^ No melting point was observed in DSC analysis.

**Table 3:** Thermal properties of d-gluconic acid derived ILs.

	Physical State	T_m_ (°C)	Tc (°C)	T_g_ (°C)	T_d_ (°C)	Ref.
**39a**	Yellow viscous liquid	---	---	---	>250 °C ^1,2,3,4^	[43]
**39b**	Pale yellow gel	---	---	---	>250 °C ^1,2,3,4^	[43]
**39c**	Yellow gel	---	---	---	>230 °C ^1,2,3,4^	[43]
**39d**	Yellow viscous liquid	---	---	---	>230 °C ^1,2,3,4^	[43]
**40a**	White solid	81.5 ^5^	---	−21.8 ^6^; 80.2 ^6^	155.0 ^7,8^	[44]
**40b**	White solid	35.6 ^5^	73.4 ^5^	34.0 ^6^	161.8 ^7,8^	[44]
**40c**	White solid	120 ^5^	---	0.4 6	172.5 ^7,8^	[44]
**43**	Yellow viscous oil	---	---	−24.1 ^9^	103.1 ^7,8^	[17]
**44a**	Light-yellow oil	---	---	−0.1 ^9^	195.1 ^7,8^	[17]
**44b**	Light yellow oil	---	---	−19.4 ^9^	191.7 ^7,8^	[17]
**44c**	Light yellow oil	---	---	−7.9 ^9^	210.3 ^7,8^	[17]
**44d**	Light yellow oil	---	---	−46.6 ^9^	127.8 ^7,8^	[17]
**44e**	Light yellow oil	---	---	−55.5 ^9^	200.6 ^7,8^	[17]
**44f**	Light yellow oil	---	---	−36.1 ^9^	200.6 ^7,8^	[17]
**44g**	Light yellow oil	87.1 ^8^	---	−22.8 ^9^	121.8 ^7,8^	[17]
**44h**	Yellow oil	---	---	−16.2 ^9^	188.5 ^7,8^	[17]
**45**	Light yellow oil	---	---	−3.7 ^9^	197.3 ^7,8^	[17]
**46a**	Light yellow oil	---	---	−16.6 ^10^	168.4 ^7,8^	[48]
**46b**	Yellow oil	---	---	−15.9 ^10^	191.1 ^7,8^	[48]

^1^ Determined by DSC; ^2^ DSC conditions: 2–8 mg of the compound; 40 °C for 10 min, then 10 °C/min to 250 °C, Crimped aluminum pan, N_2_ (50 mL/min); ^3^ The authors reported this interval of decomposition temperatures; no T_d_ is reported for each compound; ^4^ Inflection point of the endothermic peak behavior; ^5^ DSC conditions: From −10 °C to T_max_ (depending on compound stability) then cooling from T_max_ to −10 °C at 10 °C/min, two heating-cooling cycles, hermetically sealed aluminum pan, N_2_. ^6^ These data have been taken from Reference [17]. For DSC conditions see note 9; ^7^ TGA conditions: 5 °C/min to 400–500 °C, Alumina crucibles, N_2_. ^8^ Td_5%_^9^ DSC conditions: From −50 °C to T_max_ (depending on compound stability), 10 °C/min; aluminum hermetically sealed pans, N_2_; ^10^ DSC conditions: From −20 °C to T_max_ (depending on compound stability), two heating-cooling cycles, 5 °C/min, hermetically sealed aluminum pan, N_2_.

**Table 4:**
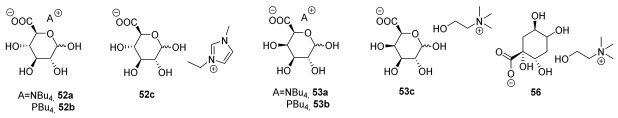
Structures and thermal properties, reported for ILs obtained from d-glucuronic, d-galacturonic, and d-quinic acid.

	Physical State	T_m_(°C)	T_g_(°C)	T_d_(°C)	Ref.
**52a**	Solid	142–143 ^1^		^2,3^ (200–300) ^4^	[54]
**52a**	Solid	95 °C ^1^	−20.5 ^5^	136 ^6,7^	[55]
**52b**	Oil	---	−8.7 ^5^	130 ^7^	[56]
**52c**	Liquid	---	---	^2,3^ (200–300, higher than 52a) ^4^	[54]
**53a**	Solid	98 °C ^1^	18.3 ^5^	n.d.^7^	[55]
**53b**	Wax	---	−22.5 ^5^	142 ^7^	[56]
**53c**	Solid	50.1 ^8^	−63.3 ^8^	245 ^9,10^	[57]
**56**	Liquid	18.8 ^8^	−76.3 ^8^	330 ^9,10^	[57]

^1^ Measured on a digital melting point apparatus; ^2^ TGA conditions: 5–10 mg, from 25 °C to 900 °C at 10 °C/min, Al_2_O_3_ crucibles, N_2_ (80 mL/min) ^3^ No value reported, only the thermograms were depicted; ^4^ Approximative T_d50%_ obtained from the reported thermograms. ^5^ DSC conditions: 10–20 mg, aluminum pan with a pierced lid, 10 °C/min, Ar (200 mL/min); ^6^ Onset temperature; ^7^TGA conditions: 10–20 mg of the compound, from 30 °C to 500 °C, 10 °C/min, dry Ar(50 mL/min), Alumina crucibles; ^8^ DSC conditions: From −100 °C to 100 °C, 2 °C/min, N_2_; ^9^ TGA conditions: From 30 °C to 450 °C, 5 °C/min, N_2_; ^10^ Decomposition temperature.

**Table 5:** Structures and thermal properties of isohexide-based ILs.

	Physical State	T_g_(°C)	T_onset_(°C)	T_d5%_(°C)	T_d10%_(°C)	T_peak_(°C)	Ref.
**78a**	Glass	−21 ^1,2^	---	---	231 ^3^	---	[68]
**78b**	Viscous material	−68 ^1,2^	---	---	232 ^3^	---	[68]
**78c**	Viscous material	−51 ^1,2^	---	---	229 ^3^	---	[68]
**78d**	Viscous material	−60 ^1,2^	---	---	245 ^3^	---	[68]
**80a**	Glass	−8 ^1,2^	---	---	238 ^3^	---	[68]
**80b**	Viscous material	−27 ^1,2^	---	---	250 ^3^	---	[68]
**80c**	Viscous material	−37 ^1,2^	---	---	251 ^3^	---	[68]
**80d**	Viscous material	−58 ^1,2^	---	---	269 ^3^	---	[68]
**83a**	Yellow solid	---	219.6 ^4,5^ 336.5 ^4,6^	218.3 ^4^	226.9 ^4^	238.6 ^4,5^ 363.5 ^4,6^	[70]
**83b**	Orange solid	---	222.3 ^4,5^	223.0 ^4^	228.7 ^4^	277.1 ^4,5^ 349.5 ^4,6^	[70]
**84a**	Yellow solid	---	181.8 ^4,5^ 227.4 ^4,6^	192.2 ^4^	228.4 ^4^	192.2 ^4,5^ 246.1 ^4,6^ 353.1 ^4,7^	[70]
**84b**	Brick red solid	79.9 ^8^	233.7 ^4^	216.1 ^4^	228.9 ^4^	288.8 ^4^	[70]
**85a**	Yellow orange glassy solid	49.3 ^8^	370.7 ^4^	358.3 ^4^	370.8 ^4^	391.7 ^4^	[70]
**85b**	Pale yellow glassy solid	31.4 ^8^	354.3 ^4^	347.8 ^4^	357.03 ^4^	366.4 ^4,5^ 382.4 ^4,6^	[70]
**86a**	Brick red glassy solid	48.1 ^8^	370.1 ^4^	362.5 ^4^	372.2 ^4^	394.8 ^4^	[70]
**86b**	Pale brown glassy solid	28.4 ^8^	370.5 ^4^	356.7 ^4^	376.6 ^4^	387.5 ^4^	[70]

^1^ DSC conditions: 20 °C/min, He; ^2^ Measured during the second heating cycle; ^3^ TGA conditions: 20 °C/min to 600 °C, N_2_; ^4^ TGA conditions: 10 °C/min from 40 °C to 900 °C, N_2_, Pt pan (100 μL). Every sample was dried in the furnace at 60 °C for 10 min under an N_2_ atmosphere prior to measurement; ^5^ Referred to the first degradation event; ^6^ Referred to the second degradation event; ^7^ Referred to the third degradation event; ^8^ DSC conditions: The samples were heated to the maximal temperature (10 °C/min) to remove thermal history, then cooled to −90 °C (5 °C/min) and heated from −90 °C to the maximal temperature (10 °C/min) (the two heating ramps lead to the same results), dry N_2_ (30 cm^3^/min), 1.44–7.21 mg, aluminum pan with a pinhole on the cap, the maximal temperature was 180 °C for **83a,b**, **84a,b**, 300 °C for **83c** and **84c,** 250 °C for **83d** and 210 °C for **84d**.

**Table 6:** Thermal properties, reported for d-glucose-derived ILs.

	Physical State	T_m_ (°C)	T_g_ (°C)	T_d_ (°C)	Ref.
**98b**	Liquid	---	−30 ^1^	433 ^2,3^(454) ^3,4^[≈400–500] ^5,6,7^	[75]
**99b**	Liquid	---	−20 ^1^	435 ^2,3^(456) ^3,4^	[75]
**100b**	Liquid	---	−14 ^1^	446 ^2,3^(460) ^3,4^	[75]
**105a**	Viscous liquid	---	−19 ^8^	198 ^6,9^(≈300) ^6.7^	[81]
**105b**	Viscous liquid	---	−18 ^8^	198 ^6,9^(≈300) ^6.7^	[81]
**105c**	Viscous liquid	---	4 ^8^	208 ^6,9^(≈300) ^6.7^	[81]
**105d**	Viscous liquid	---	−15 ^8^	107 ^6,9^(≈200–300) ^6.7^	[81]
**105e**	Viscous liquid	---	6 ^8^	209 ^6,9^(≈300) ^6.7^	[81]
**105f**	Viscous liquid	---	−9 ^8^	207 ^6,9^(≈300) ^6.7^	[81]
**105g**	Viscous liquid	---	0 ^8^	211 ^6,9^(≈300) ^6.7^	[81]
**106b**	Solid	87–88 ^10^	---	^6,11^ (≈300) ^6,7^	[54]
**111a**	Wax	---	12 ^12^	164 ^13,14^(235) ^2,13^	[74]
**111b**	Liquid	---	20 ^12^	208 ^13,14^(285) ^2,13^	[74]
**111c**	Wax	---	10 ^12^	191 ^13,14^(259) ^2,13^	[74]
**111d**	Liquid	---	13 ^12^	224 ^13,14^(327) ^2,13^	[74]
**111e**	Wax	---	8 ^12^	191 ^13,14^(287) ^2,13^	[74]
**112a**	Solid	121 ^12^	21 ^12^	178 ^13,14^(251) ^2,13^	[74]
**112b**	Solid	134 ^12^	---	202 ^13,14^(247) ^2,13^	[74]
**112c**	Wax	---	22 ^12^	201 ^13,14^(279) ^2,13^	[74]
**112d**	Wax	---	12^12^	221 ^13,14^(340) ^2,13^	[74]
**112e**	Wax	---	15^12^	204 ^13,14^(296) ^2,13^	[74]
**139b**	Solid	124 ^10^	---	^6,11^ (≈400–500, higher than 98b) ^6,7^	[54]
**139c**	Liquid	---	---	^6,11^ (≈300, lower than 106b) ^6,7^	[54]
**135a**	Liquid	---	---	225 ^11,15^	[99]
**135b**	Solid	66–70 ^10^	---	205 ^11,15^	[99]
**135c**	Solid	164–168 ^10^	---	225 ^11,15^	[99]
**135d**	Solid	95–100 ^10^	---	242 ^11,15^	[99]
**135e**	Solid	118–120 ^10^	---	215 ^11,15^	[99]
**137a**	Solid	60–63 ^10^	---	250 ^11,15^	[99]
**137b**	Solid	135–138 ^10^	---	242 ^11,15^	[99]

^1^ DSC conditions: Heating from −65 °C to 250 °C (20 °C/min), held at 250 °C, cooling from 250 °C to −65 °C (20 °C/min), N_2_ (60 mL/min); ^2^ T_d50%_; ^3^ TGA conditions: From 25 °C to 600 °C (20 °C/min), N_2_ (60 mL/min); ^4^ Decomposition temperature; ^5^ These data were taken from Reference [54]; ^6^ TGA conditions: 5–10 mg of sample, from 25 °C to 900 °C, 10 °C/min, 70 μL Al_2_O_3_ crucibles, N_2_ (80 mL/min); ^7^ T_d50%_ interpreted from the reported thermograms; ^8^ DSC conditions: From −65 °C to 150 °C, 10 °C/min, 40 μL aluminum crucibles, N_2_ (80 mL/min); ^9^ Not clearly defined; ^10^ Measured on a digital melting point apparatus; ^11^ No information on the instrumental parameters; ^12^ DSC conditions: 5–15 mg of the compound, heating from 25 °C to 150 °C (10 °C/min), then cooling from 150 °C to −100 °C (10 °C/min), N_2_, aluminum pan; ^13^ TGA conditions: 2–10 mg, from 30 °C to 450 °C (10 °C/min), N_2_; ^14^ T_d5%_ (onset point at 5% weight loss); ^15^ Decomposition point.

**Table 7:**
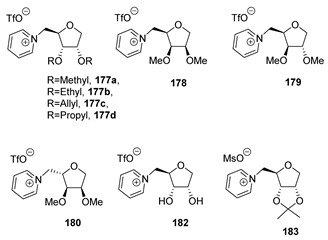
Thermal properties and structures of pentoses-derived ILs.

	Physical State	T_m_ (°C)	T_g_ (°C)	T_d_ (°C)	Ref.
**177a**	Solid	48–51 ^1^	−18 ^2^	345 ^2,3^	[99]
**177b**	Liquid		−26 ^2^	316 ^2,3^	[99]
**177c**	Liquid			301 ^2,3^	[99]
**177d**	Liquid		−27 ^2^	235 ^2,3^	[99]
**178**	Liquid		−41 ^2^	325 ^2,3^	[99]
**179**	Solid	32–36 ^1^	−28 ^2^	345 ^2,3^	[99]
**180**	Liquid		−38 ^2^	340 ^2,3^	[99]
**182**	Liquid		−30 ^2^	297 ^2,3^	[99]
**183**	Solid	92–94 ^1^		296 ^2,3^	[99]

^1^ Measured on a digital melting point apparatus; ^2^ No information on the instrumental apparatus and the experimental procedure for TGA and DSC analysis was reported; ^3^ No definition furnished.

**Table 8:**
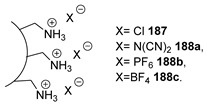
Structures and thermal properties of IL colloidal microgels derived from sucrose.

	Sugar Core	T_d_(°C)	Ref.
**187**	Sucrose microgel	---	[117]
**188a**	Sucrose microgel	≈200 ^1^ (638) ^2,3^	[117]
**188b**	Sucrose microgel	≈200 ^1^ (790) ^2,3^	[117]
**188c**	Sucrose microgel	≈200 ^1^ (840) ^2,3^	[117]

^1^ Onset temperature; ^2^ Temperature of complete decomposition; ^3^ TGA conditions: 10 °C/min to 1000 °C, N_2_ (100 mL/min). (please refer to the Appendix A of the original paper, Table A8).

**Table 9:**
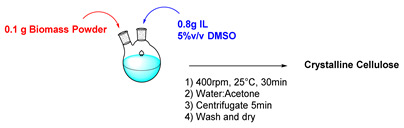
Schematic representation of cellulose extraction and cellulose recovery for the ILs studied by Javed et al. [45].

Ionic Liquid	Cellulose Recovery
**41-As**	48%
**41-Ph**	65%
**41-Ac**	62%
**41-Fo**	58%
**41-Ci**	53%
**41**	52%

**Table 10:** IC_50_ values were determined using a modification of the broth microdilution method and biodegradation determined by a closed bottle test by Ferlin et al. in 2013 [55]. The values of IC_50_ reported in the table are expressed in millimolar; TBABr and TBAOH·30H_2_O were studied as reference classical salts.

Compound	*Escherichia coli*	*Bacillus subtilis*	*Pseudomonas fluorescens*	*Pseudomonas putida* (CP1)	*Pseudomonas putida* (KT2440)	Biodegradation ^1^
**TBABr**	03.13–06.25	25.00–50.00	06.25–12.50	25.00–50.00	12.50–25.00	7.9
**TBAOH·30H_2_O**	06.25–12.50	12.50–25.00	12.50–25.00	12.50–25.00	12.50–25.00	3.8
**TEABr**	---	---	---	---	---	0
**TMABr**	---	---	---	---	---	2.6
**196a**	12.50–25.00	06.25–12.50	12.50–25.00	06.25–12.50	12.50–25.00	15.5
**196b**	06.25–12.50	03.13–06.25	03.13–06.25	03.13–06.25	12.50–25.00	15.5
**196c**	12.50–25.00	06.25–12.50	06.25–12.50	06.25–12.50	12.50–25.00	9.9
**196d**	12.50–25.00	03.13–06.25	12.50–25.00	06.25–12.50	12.50–25.00	Nd ^2^
**196e**	06.25–12.50	03.13–06.25	03.13–06.25	03.13–06.25	03.13–06.25	14.2
**196f**	12.50–25.00	06.25–12.50	06.25–12.50	06.25–12.50	12.50–25.00	12.2
**52a**	06.25–12.50	25.00–50.00	25.00–50.00	25.00–50.00	25.00–50.00	18.6
**53a**	12.50–25.00	25.00–50.00	25.00–50.00	25.00–50.00	25.00–50.00	22.8

^1^ ThOD: Theoretical oxygen demand; ^2^ Not determined due to the failing of the validation requirements for the test.

**Table 11:** Cytotoxicity and biodegradability studies, as reported by Erfurt et al. [72].

IL	EC_50_ (μmol/L) ^1^	EC_50_ (mg/L) ^1^	Biodegradability ^2^
**106a**	>584	>202	74–83% ^3^
**108c**	85.1 (66.1–97.7) ^4^	42.6 (33.1–48.9) ^4^	57% ^5^
**108d**	9.5 (8.9–10.2) ^4^	5.3 (5.0–5.7) ^4^	50–60% ^6^
**108e**	13.5 (12.3–14.5) ^4^	10.2 (9.3–10.9) ^4^	Not tested ^7^
**Carbendazim** ^8^	8.1 (7.4–8.7) ^4^	1.6 (1.4–1.7) ^4^	---

^1^ Evaluated on rat leukemia cell line IPC-81 using a WST-1 assay; ^2^ Two independent biodegradation tests were performed using the ready biodegradability test procedure according to the OECD 301F guideline; ^3^ Readily biodegradable; ^4^ Confidence interval; ^5^ Low persistence potential; ^6^ Longer lag phase (10 days), after which biodegradation started and continued until test termination without reaching a plateau, indicating that biodegradation might have proceeded further as the test continued; ^7^ Test not made; Tf_2_N^−^ anion is known to be stable against degradation [183]; ^8^ Cabendazim, a fungicide compound, was employed as a positive control.

**Table 12:**
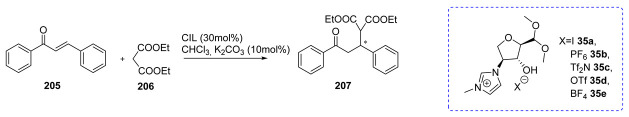
Results obtained by Jayachandra et al. in the asymmetric Michael addition reaction catalyzed by CILs **35** [40].

IL	T (°C)	t (h)	Yield (%)	*ee* (%)
**35a**	rT	7	81	14
**35a**	0 to −5	12	73	33
**35b**	rT	7	84	2
**35b**	0 to −5	12	71	8
**35c**	rT	8	76	3
**35c**	0 to −5	13	69	9
**35d**	rT	8	79	Racemic
**35d**	0 to −5	12	70	4
**35e**	rT	8	78	Racemic
**35e**	0 to −5	13	72	4

**Table 13:**
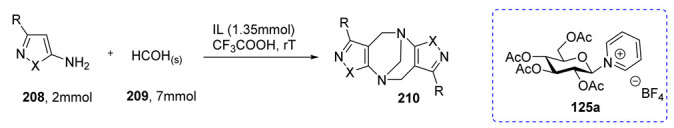
Enantioselective synthesis of chiral Troger’s bases employing a carbohydrate-derived chiral ionic liquid (CIL), as reported by Yuan et al. [83].

Entry	R	X	t (h)	Yield (%) ^1^	ee (%) ^2^
**1**	CH_3_	NPh	5	83	98
**2**	p-MePh	NPh	7	76	>99
**3**	p-OMePh	NPh	7	73	>99
**4**	CH _3_	p-MePhN	6	71	90
**5**	Ph	NPh	7.5	70	84
**6**	H	NPh	9	79	44 ^3^
**7**	p-ClPh	NPh	9	65	Racemic
**8**	CH_3_	O	10	51	Racemic

^1^ Isolated yield; ^2^ Determined by HPLC (Chiralcel OD-H, IPA:Acetonitrile 15:85); ^3^Determined by HPLC (Chiralcel OD-H, IPA:Methanol 15:85).

## Data Availability

Not applicable.

## Appendix A

In this appendix, the physical state of each ionic compound cited before is reported. Every piece of information reported in the following tables was taken from the original paper(s) reported in the last column.

**Table A1:** Physical state and melting point of ionic liquids and salts derived from d-galactose reported in this review.

	Sugar Core	Physical State	Melting Point (°C)	Ref.
**4**	d-galactose	Colorless solid	196–198	[21,22,23]
**5a**	d-galactose	Colorless solid	159–161 (142–145)	[21,23]
**5b**	d-galactose	Colorless solid	83–85	[21,22]
**5c**	d-galactose	Colorless solid	121–123	[21]
**5d**	d-galactose	Colorless solid	177–179	[21]
**6**	d-galactose	White solid	Not reported	[24]
**7a**	d-galactose	Off-white solid	Not reported	[24]
**7b**	d-galactose	White solid	Not reported	[24]
**7c**	d-galactose	Light green solid	Not reported	[24]
**7d**	d-galactose	Off white solid	Not reported	[24]
**7e**	d-galactose	White solid	Not reported	[24]
**12a**	d-galactose	Light yellow viscous fluid		[28]
**12b**	d-galactose	No information		[28]
**13a**	d-galactose	Light yellow solid	Not reported	[28]
**13b**	d-galactose	No information		[28]
**15a**	d-galactose	Oil		[32]
**15b**	d-galactose	Oil		[32]
**16a**	d-galactose	Thick syrup		[32]
**16b**	d-galactose	Thick syrup		[32]
**20**	d-galactose	Solid	62–63	[33]

**Table A2:** Physical state and melting point of ionic liquids and salts derived from d-xylose reported in this review.

	Sugar Core	Physical State	Melting Point (°C)	Ref.
**25a**	d-xylose	White wax		[34]
**25b**	d-xylose	White wax		[34]
**26**	d-xylose	Oily		[38]
**27a**	d-xylose	White solid	59	[38]
**27b**	d-xylose	Oily		[38]
**27c**	d-xylose	White solid	98	[38]
**28a**	d-xylose	White solid	74	[38]
**28b**	d-xylose	Oily		[38]
**28c**	d-xylose	White solid	112	[38]
**29a**	d-xylose	Oily		[38]
**29b**	d-xylose	Oily		[38]
**29c**	d-xylose	Oily		[38]
**35a**	d-xylose	Light brown color liquid		[39,40]
**35b**	d-xylose	Light brown color liquid		[39,40]
**35c**	d-xylose	Light brown color liquid		[39,40]
**35d**	d-xylose	Light brown color liquid		[39,40]
**35e**	d-xylose	Light brown color liquid		[39,40]

**Table A3:** Physical state and melting point of ionic liquids and salts derived from d-gluconic acid reported in this review.

	Sugar Core	Physical State	Melting Point (°C)	Ref.
**39a**	d-gluconic acid	Yellow viscous liquid		[43]
**39b**	d-gluconic acid	Pale yellow gel		[43]
**39c**	d-gluconic acid	Yellow viscous liquid		[43]
**39d**	d-gluconic acid	Yellow gel		[43]
**40a**	d-gluconic acid	Solid	81.5	[17,44]
**40b**	d-gluconic acid	Solid	35.6	[17,44]
**40c**	d-gluconic acid	Solid	134.9	[17,44]
**41**	d-gluconic acid	Liquid	25–30	[45]
**42a**	d-gluconic acid	Classified as IL by the authors. No physical state reported.		[46]
**42b**	d-gluconic acid	Classified as IL by the authors. No physical state reported.		[46]
**42c**	d-gluconic acid	Classified as IL by the authors. No physical state reported.		[46]
**42d**	d-gluconic acid	Classified as IL by the authors. No physical state reported.		[46]
**42e**	d-gluconic acid	Classified as IL by the authors. No physical state reported.		[46]
**42f**	d-gluconic acid	Classified as IL by the authors. No physical state reported.		[46]
**42g**	d-gluconic acid	Classified as IL by the authors. No physical state reported.		[46]
**43**	d-gluconic acid	Yellow viscous oil		[17]
**44a**	d-gluconic acid	Liquid		[17]
**44b**	d-gluconic acid	Light yellow oil		[17]
**44c**	d-gluconic acid	Light yellow oil		[17]
**44d**	d-gluconic acid	Light yellow oil		[17]
**44e**	d-gluconic acid	Light yellow oil		[17]
**44f**	d-gluconic acid	Light yellow oil		[17]
**44g**	d-gluconic acid	Light yellow oil		[17]
**44h**	d-gluconic acid	Light yellow oil		[17]
**45**	d-gluconic acid	Light yellow oil		[17]
**46a**	d-gluconic acid	Light yellow oil		[48]
**46b**	d-gluconic acid	Yellow oil		[48]
**47a**	d-gluconic acid	White solid	88.2–90.1	[47,49]
**47b**	d-gluconic acid	White solid	95.1–96.6	[47,49]
**47c**	d-gluconic acid	White	96.7–98.4	[47,49]
**48a**	d-gluconic acid	Brown-yellow syrup		[47,50]
**48b**	d-gluconic acid	White solid	76.5–78.3	[47,50]
**48c**	d-gluconic acid	White solid	78.9–83.1	[47,50]
**48d**	d-gluconic acid	White solid	93.5–94.6	[47,50]
**49a**	d-gluconic acid	Brown-yellow syrup		[47,51]
**49b**	d-gluconic acid	Brown-yellow syrup		[47,51]
**49c**	d-gluconic acid	Brown-yellow syrup		[47,51]

**Table A4:** Physical state and melting point of ionic liquids and salts derived from d-glucuronic acid, d-galacturonic acid, (−)-quinic acid, clindamycin phosphate, lactobionic acid and d-glucosamine reported in this review.

	Sugar Core	Physical State	Melting Point (°C)	Ref.
**52a**	d-glucuronic acid	White solid	95	[54,55]
**52b**	d-glucuronic acid	Viscous oil		[56]
**52c**	d-glucuronic acid	Viscous liquid		[54]
**53a**	d-galacturonic acid	White solid	98	[55]
**53b**	d-galacturonic acid	Wax		[56]
**53c**	d-galacturonic acid	Solid	50.1	[57]
**56**	(−)-quinic acid	Liquid	18.8	[57]
**57**	Clindamycin phosphate	Pale yellow viscous liquid		[59]
**60a**	Lactobionic acid	Brown-yellow syrup		[50]
**60b**	Lactobionic acid	Light yellow solid	99.5–103.2	[50]
**60c**	Lactobionic acid	Light yellow solid	129.8–134.5	[50]
**63**	Lactobionic acid	Colorless viscous liquid		[60]
**65**	d-glucosamine	Classified as IL by the authors. No physical state reported.		[61]

**Table A5:** Physical state and melting point of ionic liquids and salts derived from isohexides reported in this review.

	Sugar Core	Physical State	Melting Point	Ref.
**69a**	Isoidide	Yellow oil (Foamy material)	(Not reported)	[65,67]
**69b**	Isoidide	Solid	150–152	[65]
**69c**	Isoidide	Solid	Not reported	[65]
**69d**	Isoidide	Solid	118–122	[65]
**69e**	Isoidide	Viscous oil		[65]
**71a**	Isosorbide	Not pure		[65]
**71b**	Isosorbide	Not pure		[65]
**72a**	Isosorbide	Yellow oil		[65]
**72b**	Isosorbide	Solid	130–133	[65]
**69f**	Isoidide	Solid	Not reported	[67]
**74a**	Isosorbide	White solid	118.5–120.5	[66]
**74b**	Isosorbide	Not isolated		[66]
**74c**	Isosorbide	Not Isolated		[66]
**74d**	Isosorbide	Not Isolated		[66]
**76a**	Isoidide	Transparent oil		[66]
**76b**	Isoidide	Transparent oil		[66]
**78a**	Isomannide	Glassy orange material		[68]
**78b**	Isomannide	Transparent viscous material		[68]
**78c**	Isomannide	Orange viscous material		[68]
**78d**	Isomannide	Orange viscous material		[68]
**80a**	Isoidide	Glassy orange material		[68]
**80b**	Isoidide	Orange viscous material		[68]
**80c**	Isoidide	Orange viscous material		[68]
**80d**	Isoidide	Orange viscous material		[68]
**83a**	Isomannide	Yellow solid	Degraded before melting	[70]
**83b**	Isomannide	Orange solid	Degraded before melting	[70]
**85a**	Isomannide	Yellow orange glassy solid		[70]
**85b**	Isomannide	Pale yellow glassy solid		[70]
**84a**	Isosorbide	Yellow solid	Degraded before melting	[70]
**84b**	Isosorbide	Brick red solid	79.9	[70]
**86a**	Isosorbide	Brick red glassy solid		[70]
**86b**	Isosorbide	Pale brown glassy solid		[70]

**Table A6:** Physical state and melting point of ionic liquids and salts derived from d-glucose reported in this review.

	Sugar Core	Physical State	Melting Point (°C)	Ref.
**94**	d-glucose	No information		[80]
**95**	d-glucose	Oil		[80]
**96**	d-glucose	Yellow oily solid		[80]
**97**	d-glucose	No information		[80]
**98a**	d-glucose	White solid	Not reported	[75,76]
**98b**	d-glucose	Liquid		[75,76]
**99a**	d-glucose	White solid	Not reported	[75]
**99b**	d-glucose	Liquid		[75]
**100a**	d-glucose	White solid	Not reported	[75]
**100b**	d-glucose	Liquid		[75]
**101a**	d-glucose	Pale yellow syrup		[78]
**101b**	d-glucose	Pale yellow syrup		[78]
**101c**	d-glucose	Pale yellow syrup		[78]
**102a**	d-glucose	Pale yellow syrup		[78]
**102b**	d-glucose	Pale yellow syrup		[78]
**102c**	d-glucose	Pale yellow syrup		[78]
**103a**	d-glucose	Red brown syrup		[77]
**103b**	d-glucose	Red syrup		[77]
**103c**	d-glucose	Red syrup		[77]
**104a**	d-glucose	Pale yellow syrup		[77]
**104b**	d-glucose	Pale yellow syrup		[77]
**104c**	d-glucose	Pale yellow syrup		[77]
**105a**	d-glucose	Viscous liquid		[81]
**105b**	d-glucose	Viscous liquid		[81]
**105c**	d-glucose	Viscous liquid		[81]
**105d**	d-glucose	Viscous liquid		[81]
**105e**	d-glucose	Viscous liquid		[81]
**105f**	d-glucose	Viscous liquid		[81]
**105g**	d-glucose	Viscous liquid		[81]
**106a**	d-glucose	Colorless oil		[32,54,72,74]
**106b**	d-glucose	White solid	87–88	[54]
**106c**	d-glucose	Thick syrup		[32,54]
**107a**	d-glucose	White solid	124.2–128.6	[32]
**107c**	d-glucose	Thick syrup		[32]
**108a**	d-glucose	White powder		[72,74]
**108b**	d-glucose	White powder		[72,74]
**108c**	d-glucose	White powder		[72,74]
**108d**	d-glucose	White powder		[72,74]
**108e**	d-glucose	Not reported		[72,74]
**109a**	d-glucose	Viscous liquid		[73]
**109b**	d-glucose	Viscous liquid		[73]
**109c**	d-glucose	Viscous liquid		[73]
**109d**	d-glucose	White solid	Not reported	[73]
**109e**	d-glucose	White solid	Not reported	[73]
**109f**	d-glucose	White solid	Not reported	[73]
**109a**	d-glucose	Wax		[74]
**109b**	d-glucose	Liquid		[74]
**111c**	d-glucose	Wax		[74]
**111d**	d-glucose	Liquid		[74]
**111e**	d-glucose	Wax		[74]
**112a**	d-glucose	Solid	121	[74]
**112b**	d-glucose	Solid	134	[74]
**112c**	d-glucose	Wax		[74]
**112d**	d-glucose	Wax		[74]
**112e**	d-glucose	Wax		[74]
**116a**	d-glucose	Yellow syrup		[79]
**116b**	d-glucose	Not pure		[79]
**116c**	d-glucose	Yellow syrup		[79]
**116d**	d-glucose	Not pure		[79]
**116e**	d-glucose	Yellow syrup		[79]
**116f**	d-glucose	Yellow syrup		[79]
**117a**	d-glucose	Yellow syrup		[79]
**117b**	d-glucose	Not pure		[79]
**117c**	d-glucose	Yellow syrup		[79]
**117d**	d-glucose	Not pure		[79]
**117e**	d-glucose	Yellow syrup		[79]
**117f**	d-glucose	Yellow syrup		[79]
**118b**	d-glucose	Not reported, micellar synthesis		[79]
**122**	d-glucose	No information, they only say IL		[82]
**124a**	d-glucose	No information, they only say IL		[82]
**124b**	d-glucose	No information, they only say IL		[82]
**125a**	d-glucose	Yellow solid	94.6–98	[83]
**125b**	d-glucose	Not isolated		[83]
**126a**	d-glucose	Pale yellow viscous compound		[85]
**126b**	d-glucose	Pale yellow viscous compound		[85]
**126c**	d-glucose	Pale yellow viscous compound		[85]
**126d**	d-glucose	Pale yellow viscous compound		[85]
**127a**	d-glucose	Colorless viscous compound		[86,87]
**127b**	d-glucose	Colorless viscous compound		[86]
**127c**	d-glucose	Colorless viscous compound		[86]
**127d**	d-glucose	Colorless viscous compound		[86]
**128a**	d-glucose	Colorless viscous compound		[84]
**128b**	d-glucose	Colorless viscous compound		[84]
**128c**	d-glucose	Not reported		[84]
**128d**	d-glucose	Not reported		[84]
**128e**	d-glucose	Colorless viscous compound		[87]
**128f**	d-glucose	Colorless viscous compound		[87]
**128g**	d-glucose	Solid	Not reported	[87]
**129**	d-glucose	White solid	229–231	[88]
**130**	d-glucose	Oil		[88]
**135a**	d-glucose	Liquid		[99]
**135b**	d-glucose	Solid	66–70	[99]
**135c**	d-glucose	Solid	164–172	[99]
**135d**	d-glucose	Liquid		[99]
**135e**	d-glucose	Solid	114–116	[99]
**137a**	d-glucose	Solid	60–63	[99]
**137b**	d-glucose	Solid	135–137	[99]
**139a**	d-glucose	Not reported		[54]
**139b**	d-glucose	White solid	124	[54]
**139c**	d-glucose	Viscous Liquid		[54]
**144-Br**	d-glucose	Not reported (polymer)		[100]
**144-BF_4_**	d-glucose	Not reported (polymer)		[100]
**144-PF_6_**	d-glucose	Not reported (polymer)		[100]
**144-Tf_2_N**	d-glucose	Not reported (polymer)		[100]

**Table A7:** Physical state and melting point of ionic liquids and salts derived from *N*-methyl-d-glucamine reported in this review.

	Sugar Core	Physical State	Melting Point (°C)	Ref.
**146**	*N*-methyl-d-glucamine	Classified as IL by the authors. No physical state reported.		[109]
**147**	*N*-methyl-d-glucamine	Solid	96–98	[102]
**148**	*N*-methyl-d-glucamine	Not isolated		[108]
**149a**	*N*-methyl-d-glucamine	No information (polymer)		[107]
**149b**	*N*-methyl-d-glucamine	Classified as IL by the authors. No physical state reported.		[107,110]
**149c**	*N*-methyl-d-glucamine	No information (polymer)		[107,110]
**150a**	*N*-methyl-d-glucamine	Classified as IL by the authors. No physical state reported.		[112]
**150b**	*N*-methyl-d-glucamine	Classified as IL by the authors. No physical state reported.		[112]
**149d**	*N*-methyl-d-glucamine	Liquid (the authors reported the melting point for solid compounds)		[103,104]
**152a**	*N*-methyl-d-glucamine	Liquid (the authors reported the melting point for solid compounds)		[103]
**152b**	*N*-methyl-d-glucamine	Liquid (the authors reported the melting point for solid compounds)		[103]
**152b**	*N*-methyl-d-glucamine	Liquid (the authors reported the melting point for solid compounds)		[103]
**150c**	*N*-methyl-d-glucamine	Classified as IL by the authors. No physical state reported.		[105]

**Table A8:** Physical state and melting point of ionic liquids and salts derived from d-mannose reported in this review.

	Sugar Core	Physical state	Melting Point (°C)	Ref.
**153a**	d-mannose	No information		[80]
**154a**	d-mannose	No information		[80]
**154b**	d-mannose	Oil		[80]
**153b**	d-mannose	Yellow oily solid		[80]
**156a**	d-mannose	Oil		[32]
**156b**	d-mannose	Thick oil		[32]
**157a**	d-mannose	Oil (In “Experimental section” the authors reported “Solid, mp 225.1–260.3 °C”)		[32]
**158b**	d-mannose	Yellow oil		[32]
**161-Br**	d-mannose	Not reported (polymer)		[100]
**161-BF4**	d-mannose	Not reported (polymer)		[100]
**161-PF6**	d-mannose	Not reported (polymer)		[100]
**161-Tf2N**	d-mannose	Not reported (polymer)		[100]
**162**	d-mannose	White solid glassy material	49–50	[33]

**Table A9:** Physical state and melting point of ionic liquids and salts derived from pentoses reported in this review.

	Sugar Core	Physical State	Melting Point (°C)	Ref.
**167a**	d-ribose	Light brown color solid	157–160	[21]
**167b**	d-ribose	Yellow color solid	124–126	[21]
**167c**	d-ribose	Oily liquid		[21]
**167d**	d-ribose	Oily liquid		[21]
**167e**	d-ribose	Colorless solid	96–98	[21]
**167f**	d-ribose	Oily liquid		[21]
**168**	d-ribose	Solid	75–78	[115]
**177a**	1-deoxyribose	Solid	48–51	[99]
**178**	1-deoxylyxose	Liquid		[99]
**179**	1-deoxyxylose	Solid	32–36	[99]
**180**	1-deoxyarabinose	Liquid		[99]
**177b**	1-deoxyribose	Liquid		[99]
**177c**	1-deoxyribose	Liquid		[99]
**177d**	1-deoxyribose	Liquid		[99]
**182**	1-deoxyribose	Liquid		[99]
**183**	1-deoxyribose	Solid	92–94	[99]

**Table A10:** Physical state and melting point of ionic liquids and salts derived from disaccharides reported in this review.

	Sugar Core	Physical State	Melting Point (°C)	Ref.
**187**	Sucrose	Microgel		[117]
**188a**	Sucrose	Microgel		[117]
**188b**	Sucrose	Microgel		[117]
**188c**	Sucrose	Microgel		[117]
**192a**	Lactose	Red syrup		[77]
**192b**	Lactose	Red syrup		[77]
**192c**	Lactose	Red syrup		[77]
**193a**	Lactose	Red syrup		[77]
**193b**	Lactose	Red syrup		[77]
**193c**	Lactose	Red syrup		[77]
**195a**	Xyloside	White solid	Not reported	[38]
**195b**	Xyloside	White solid	93	[38]
**195c**	Xyloside	Oily		[38]
